# Analysis of transcriptional response to heat stress in *Rhazya stricta*

**DOI:** 10.1186/s12870-016-0938-6

**Published:** 2016-11-14

**Authors:** Abdullah Y. Obaid, Jamal S. M. Sabir, Ahmed Atef, Xuan Liu, Sherif Edris, Fotouh M. El-Domyati, Mohammed Z. Mutwakil, Nour O. Gadalla, Nahid H. Hajrah, Magdy A. Al-Kordy, Neil Hall, Ahmed Bahieldin, Robert K. Jansen

**Affiliations:** 1 Department of Biological Sciences, Faculty of Science, King Abdulaziz University (KAU), P.O. Box 80141, Jeddah, 21589 Saudi Arabia; 2 Institute of Integrative Biology, University of Liverpool, Liverpool, L69 7ZB UK; 3 Department of Genetics, Faculty of Agriculture, Ain Shams University, Cairo, Egypt; 4 Princess Al-Jawhara Al-Brahim Centre of Excellence in Research of Hereditary Disorders (PACER-HD), Faculty of Medicine, King Abdulaziz University (KAU), Jeddah, Saudi Arabia; 5 Genetics and Cytology Department, Genetic Engineering and Biotechnology Division, National Research Center, Dokki, Egypt; 6 Department of Integrative Biology, University of Texas at Austin, Austin, TX 78712 USA

**Keywords:** Thermotolerance, HSP, Chaperones, HSF, Cyclin, U-box, Aquaporine, Protein transparent testa 12, AP2-EREBP, WRKY27

## Abstract

**Background:**

Climate change is predicted to be a serious threat to agriculture due to the need for crops to be able to tolerate increased heat stress. Desert plants have already adapted to high levels of heat stress so they make excellent systems for identifying genes involved in thermotolerance. *Rhazya stricta* is an evergreen shrub that is native to extremely hot regions across Western and South Asia, making it an excellent system for examining plant responses to heat stress. Transcriptomes of apical and mature leaves of *R. stricta* were analyzed at different temperatures during several time points of the day to detect heat response mechanisms that might confer thermotolerance and protection of the plant photosynthetic apparatus.

**Results:**

Biological pathways that were crosstalking during the day involved the biosynthesis of several heat stress-related compounds, including soluble sugars, polyols, secondary metabolites, phenolics and methionine. Highly downregulated leaf transcripts at the hottest time of the day (40–42.4 °C) included genes encoding cyclin, cytochrome p450/secologanin synthase and U-box containing proteins, while upregulated, abundant transcripts included genes encoding heat shock proteins (HSPs), chaperones, UDP-glycosyltransferase, aquaporins and protein transparent testa 12. The upregulation of transcripts encoding HSPs, chaperones and UDP-glucosyltransferase and downregulation of transcripts encoding U-box containing proteins likely contributed to thermotolerance in *R. stricta* leaf by correcting protein folding and preventing protein degradation. Transcription factors that may regulate expression of genes encoding HSPs and chaperones under heat stress included HSFA2 to 4, AP2-EREBP and WRKY27.

**Conclusion:**

This study contributed new insights into the regulatory mechanisms of thermotolerance in the wild plant species *R. stricta*, an arid land, perennial evergreen shrub common in the Arabian Peninsula and Indian subcontinent. Enzymes from several pathways are interacting in the biosynthesis of soluble sugars, polyols, secondary metabolites, phenolics and methionine and are the primary contributors to thermotolerance in this species.

**Electronic supplementary material:**

The online version of this article (doi:10.1186/s12870-016-0938-6) contains supplementary material, which is available to authorized users.

## Background

Predicted climate changes are likely to represent serious threats to agriculture and food safety [1–3]. By the end of this century maximum temperature is expected to increase by more than 2 °C depending on industrial emmissions [4]. Heat stress poses one of the greatest detrimental effects on the growth and productivity of crop plants [5]. These effects include some physiological alterations in the leaf such as low photosynthetic rate and changes in metabolite accumulation [6–8]. The overall effect of the combined stress is altered protein homeostasis [9], including the control of protein synthesis, intracellular sorting, folding and degradation [10].

Tolerance to heat stress is a multigenic process with many regulatory mechanisms acting during plant development [11, 12]. Heat stress injury and response are more evident in plant leaves [1] and pollen [13] during sexual reproduction than other tissues. Plants respond to heat stress by synthesizing heat shock proteins [14] (HSPs). Transcript abundance of HSPs along with chaperones has been shown to be involved in heat stress tolerance [15, 16]. Heat shock proteins are considered molecular chaperones (e.g*.*, HSP90, HSP70 and HSP60) that control stability and folding of other proteins to protect misfolded proteins from irreversible aggregation [17–20].

In general, plant cells tolerate heat stress by orchestrating energy metabolism between dissimilation and assimilation [21], by scavenging antioxidant enzymes [22] and by reducting detoxification of reactive oxygen species (ROS) responsible for the peroxidation of membrane lipids and pigments, which causes loss of membrane permeability [23, 24]. The latter action requires high levels of expression of antioxidant genes to help confer heat tolerance in plants.

Omics has been used extensively to provide valuable information for breeding programs to improve plant thermotolerance. In recent reports, ~5% of plant transcripts were highly upregulated due to heat stress [25–27]. Upregulated transcripts include those encoding chaperones [7, 25], while others are involved in calcium/phytohormone/lipid signaling, phosphorylation, sugar accumulation, secondary metabolism and many other biological processes [28].

Transcription factors (TFs) represent key proteins required for the regulation of almost all biosynthetic pathways in life [29]. They are important for the development of organisms and for all cellular functions and responses to biotic and abiotic stresses [30]. In a previous study [31], a number of important TF families were identified in the perennial evergreen C_3_ desert shrub *Rhazya stricta* by Mapman analysis. This shrub grows well in its arid environment under high temperatures and vapor pressure deficits. The expression of gene families encoding the basic helix-loop-helix (bHLH), homeobox domain (HB), MYB as well as AP2-ERF significantly decreased at midday [31].

In the present study, we extended previous efforts of Yates et al. [31] by studying the transcriptomes of the apical and mature leaves of *Rhazya stricta* at differenent day time temperatures to gain new insights into heat response mechanisms that are involved in thermotolerance and protection of the plant photosynthetic apparatus. Such mechanisms might be a target for improving thermotolerance of economically important crop plants via transgenesis.

## Results and discussion

### Clusters of gene expression at different temperatures across times of the day

**Fig. 1:**
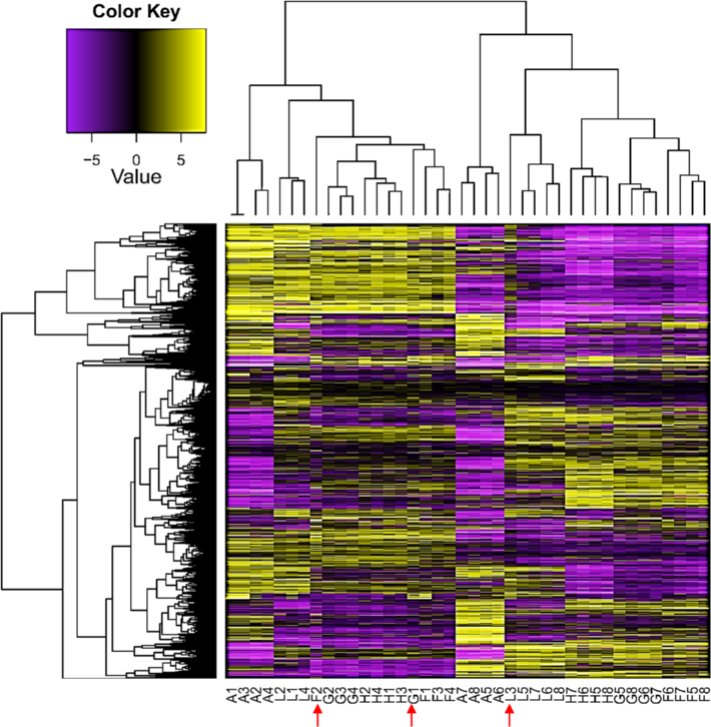
Hierarchical cluster analysis of gene expression based on log ratio RPKM data for transcriptome of *R. stricta* SRA database in the apical (A1-L4) and mature leaves (A5-L8) at different time points of the day (A, morning; F-H, midday & L, dusk). Red arrows indicate the misplaced samples in the cluster analysis

**Fig. 2:**
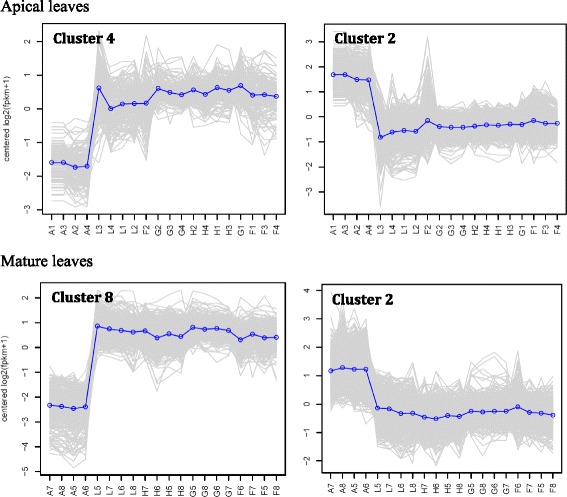
Selected clusters of up or downregulated genes of *R. stricta* from apical (A1-L4) and mature (A5-L8) leaves at different time points of the day (A, morning; F-H, midday and L, dusk). Clusters 4 and 2 of apical leaves = up and downregulation starting midday, respectively. Clusters 8 and 2 of mature leaves = up and downregulation starting midday, respectively. Blue lines indicate overall expression pattern across different transcripts of a given cluster

RNA-Seq analysis was used to analyse apical and mature leaves to test if heat responsive genes are expressed similarly in the two different leaf types. These two types of leaves differ in their developmental stages and status of cell division, which might affect heat-responsive genes differently. We speculated that this plant organ would provide a wealth of information in terms of the responsive gene families and biological pathways under heat stress. Temperatures (40–42.4 °C) at the three midday time points (13:25, 14:05, 14:30) were 12.6–15 °C higher than the morning time point (07:10) temperatures (27.4 °C), confirming that *Rhazya stricta* was experiencing heat stress during midday time points as compared to the morning. We speculate that more accurate results will be gained when comparing transcriptomes of the same plant across different time points, e.g., dawn (non-stressed) vs midday (stressed), rather than comparing transcriptomes of stressed vs. non-stressed plants at a given time point, e.g., midday. Furthermore, it is difficult to control environmental conditions for plants growing in the field. Hierarchical cluster analysis of gene expression based on log ratio RPKM data for transcripts of *R. stricta* SRA database in the apical and mature leaves at different time points of the day indicated the high quality of sampling and RNA-Seq analysis as evidenced by within timepoint clustering of replicates in 37 of the 40 samples (Fig. 1). Similar conclusions were reached when studying the genes with different expression patterns in the apical and mature leaves (Additional file 1: Table S1 & Additional file 2: Table S2 and Additional file 3: Figure S1 & Additional file 4: Figure S2, respectively). The only non-concordant samples (Fig. 1, red arrows) were the apical leaf samples F2, G1 and L3; F2 clustered with the apical leaf samples at dusk (L), G1 with apical leaf samples at time point F at midday and L3 with mature leaf samples at dusk. In general, the sampling of mature leaves resulted in more homogenous data than the apical leaves. The number of DE transcripts resulting from the RNA-Seq analysis of apical leaves across different time points was 2507 in 32 clusters (Additional file 1: Table S1). The number of DE transcripts across time points in mature leaves was 4853 in 38 clusters (Additional file 2: Table S2). We can infer that a key reason for the larger number of genes enriched in the mature leaves across the day compared to apical meristimic leaves is that the latter is more active in cell division and cell differentiation [32]. Clusters with up or downregulation starting at midday that were utilized frequently for both leaf types are shown in Fig. 2.

Semi-quantitative RT-PCR of 10 randomly selected genes was used to validate the RNA-Seq data with three replicates of both types of leaves across the three time points, e.g., morning (A), midday (F-H) and dusk (L) (Additional file 5: Figure S3). Expression patterns of these 10 genes included upregulation starting at midday and gradual downregulation (Additional file 6: Table S3). The results of semi-quantitative RT-PCR for the selected genes confirmed the fold change in the RNA-Seq data across the two types of leaves and three time points.

### Analysis of differentially expressed genes

#### KEGG analysis

**Fig. 3:**
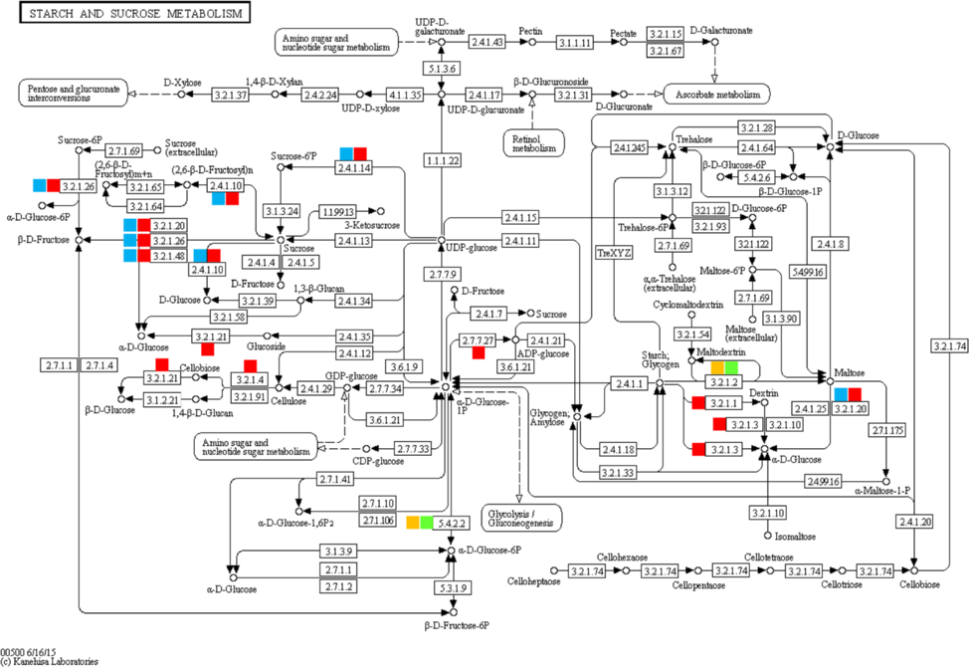
Enzymes in the starch and sucrose metabolic pathway in apical and mature leaves responded differentially to changing environment at two time points (morning, A and midday. G). Upregulated (activated) in apical leaves (*blue*), upregulated in mature leaves (red), downregulated (repressed) in apical leaves (orange box), downregulated in mature leaves (*green box*)

**Fig. 4:**
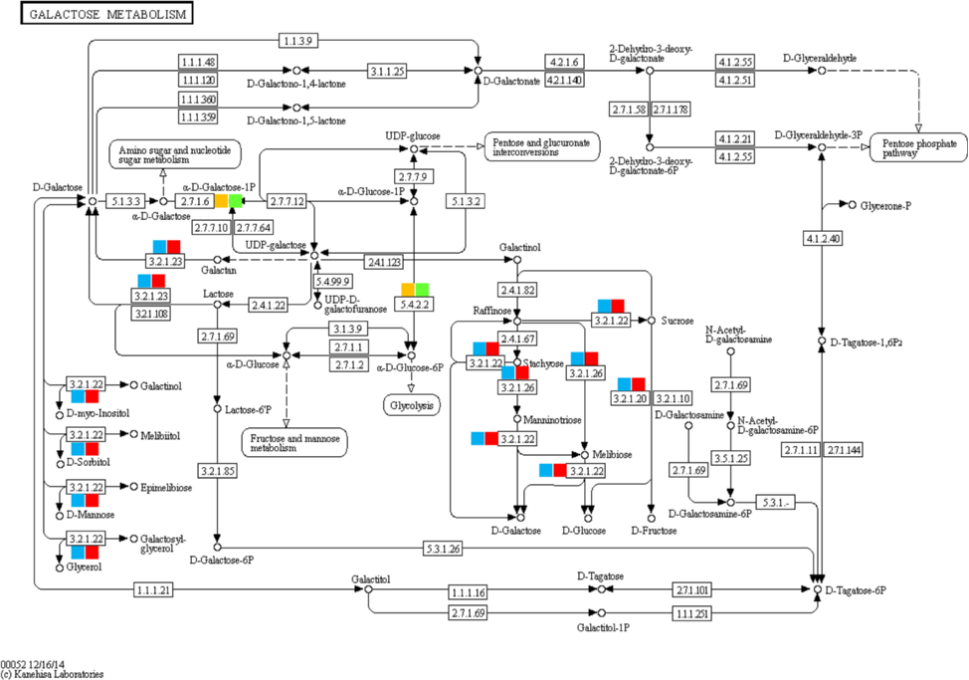
Enzymes in the galactose metabolic pathway in apical and mature leaves responded differentially to changing environments at two time points (morning, A and midday, G). Upregulated (activated) in apical leaves (blue), upregulated in mature leaves (*red*), downregulated (repressed) in apical leaves (*orange box*), downregulated in mature leaves (*green box*)

To identify the biological pathways that are active in the apical and mature leaves of *R. stricta* during the day, we mapped the detected genes to reference canonical pathways in the Kyoto Encyclopedia of Genes and Genomes (KEGG) (http://www.genome.ad.jp/kegg/). Heat tolerance is a multigenic process with different metabolic pathways affecting plant growth [12]. Enzymes with roles in the pathways that showed regulation during the day under heat stress were examined in apical and mature leaves (Table 1 and Figs. 3 and 4 and Additional file 7: Figure S4, Additional file 8: Figure S5, Additional file 9: Figure S6, Additional file 10: Figure S7 and Additional file 11: Figure S8).

**Table 1:**
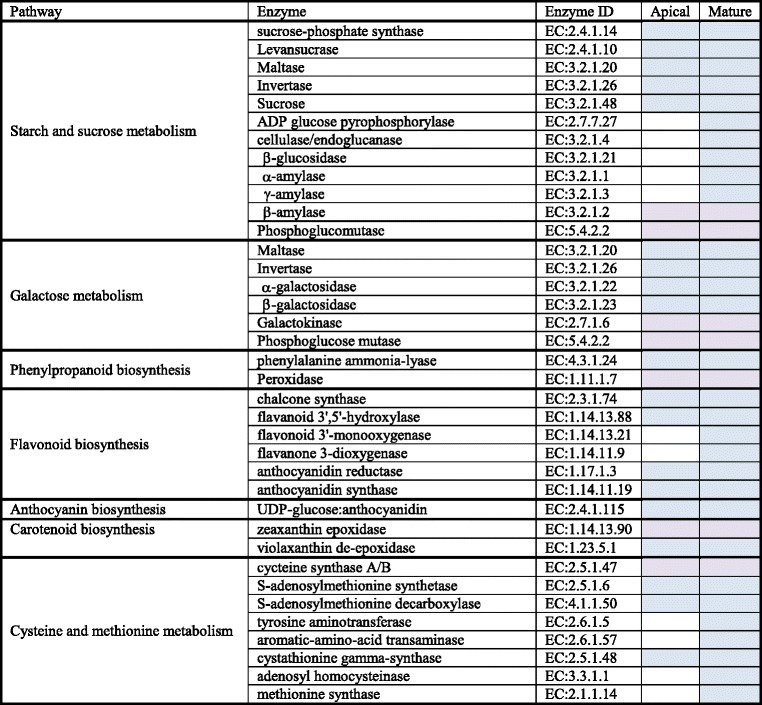
Description of the differentially responding enzymes in apical and mature leaves to changing environments at two time points (e.g., A, morning and G, midday). Activated (blue), repressed (orange)

In general, KEGG analysis indicated that the biosynthesis of soluble sugars, polyols, secondary metabolites, phenolics and methionine are involved in conferring thermotolerance in *R. stricta*. The results of starch and sucrose metabolism pathway indicated the involvement of 12 enzymes in the response to heat stress (Table 1 and Fig. 3). The most evident responses are the synthesis of several soluble sugars, e.g., sucrose, fructose and glucose, and the depletion of starch and maltose mostly by the action of sucrose-phosphate synthase, levansucrase, maltase, sucrase and invertase. Earlier reports on sucrose phosphate synthase and invertase in mulberry and soybean documented their repression under heat stress [33, 34]. Hence, soluble sugars were not accumulated as a response to heat stress during the day in these two plant species. Depletion of starch in *R. stricta* during the day towards the production of soluble sugars can be considered a favorable action only at night. Therefore, the activation of ADP glucose pyrophosphorylase (AGPase) under heat stress during the day in leaves of *R. stricta* leads to the synthesis of glycogen, which provides a continuous supply of starch during the day. This transition maximizes cytosolic carbon-sink strength in the cell [35]. Krasensky and Jonak [36] also indicated an active role of AGPase and other enzymes in starch production in the plastid during photosynthesis. It is unlikely that glycogen is converted to maltose during the day in leaves of *R. stricta* as the enzyme responsible for this action, β-amylase [37, 38], was repressed. Therefore, we can conclude that both soluble sugars and starch are favorably accumulated in *R. stricta* during the day.

**Fig. 5:**
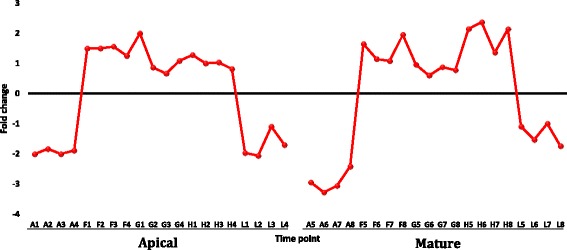
Fold change values for the gene encoding Rubisco in apical (A1-L4) and mature (A5-L8) leaves during the day (A, morning; F-H, midday & L, dusk) in *R. stricta*

**Fig. 6:**
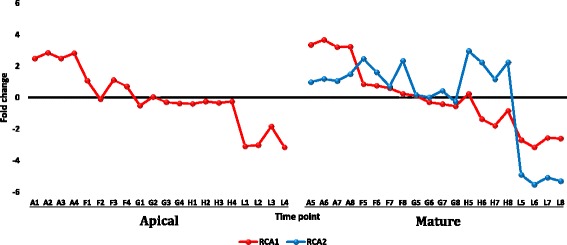
Fold change values for the genes encoding the two Rubisco activase isoforms (RCA1 and RCA2) in apical (A1-L4) and mature (A5-L8) leaves during the day (A, morning; F-H, midday & L, dusk) in *R. stricta*

Lawson et al. [39] found evidence for thermotolerance while studying photosynthetic capacity in *R. stricta* at the same time period and under the same field conditions as our study. The evidence involved the occurrence of a maximum *in vivo* carboxylation capacity of the thermostable Rubisco [40] (up to 50 °C). The recorded temperature during Lawson’s and our experiments was 43 °C. Salvucci and Crafts-Brandner [41] indicated that the thermal instability of the two Rubisco activase (RCA) isoforms at such high temperatures is a major limitation to photosynthetic capacity. The enzyme plays an important regulatory role in photosynthesis as it catalyses the removal of the sugar phosphates from the Rubisco catalytic sites [42]. Sugar phosphates are known for their action in retarding photosynthesis as they bind to Rubisco and prevent the carbamylation process [42]. The results of the present study support the results of Lawson et al. [39] because we detected a gene encoding rubisco subunit binding-protein alpha that was upregulated in the two leaf types during midday (Fig. 5). The encoded protein binds Rubisco small and large subunits and is implicated in the assembly of the enzyme oligomer. Upregulation of this gene during midday secures the continuous supply of the thermostable Rubisco during photosynthesis. In addition, the two RCA forms (RCA1 and RCA2), which represent the weak link to appropriate photosynthetic capacity under heat stress, were detected in the mature leaf of *R. stricta*, while only one form was detected in the apical leaf. These enzyme isoforms were downregulated in the present study only at dusk (Fig. 6). The continuous expression of the two RCA genes during the day secures the biosynthesis of the enzyme isoforms under heat stress, thus promoting photosynthesis. These results add to the understading of the mechanisms of thermotolerance in *R. stricta*.

Results of the enzyme activity in the galactose metabolism pathway under heat stress in leaves of *R. stricta* support the accumulation of soluble sugars (e.g., sucrose, glucose and galactose), as well as in the synthesis of several polyols (e.g., myo-inositol, sorbitol, mannose, glycerol) due to the activity of α-galactosidase (or melibiase) (Table 1 and Fig. 4). The analogue of this enzyme, i.e., β-galactosidase (or lactase), is involved in the synthesis of galactose via the conversion of galactan and lactose. Starch and glycogen are known for their sensitivity to changing environments [43–46]. The metabolism of either compound is important for the storage of carbon and energy in the cell [47]. Activities of enzymes involved in starch and sucrose metabolism and galactose metabolism during the day in leaves of *R. stricta* resulted in the accumulation of soluble sugars that can act as osmolytes to maintain cell turgor and protect membranes and proteins from damage caused by different abiotic stresses. Polyols are compatible solutes with the ability to stabilize proteins and scavenge hydroxyl radicals towards the prevention of oxidative damage of membranes and enzymes under abiotic stresses, including heat stress [48]. In agreement with our results, many reports indicated that stress tolerant plants accumulate larger amounts of protective metabolites, such as soluble sugars and polyols, under adverse conditions [40, 44]. Rosa et al. [49] also found that sucrose and hexoses upregulate growth-related genes, while downregulating stress-related genes. This dual response likely assures proper growth under unfavorable conditions in *R. stricta*.

Many secondary metabolites are synthesized from the intermediates of primary carbon metabolism [50]. Important enzymes in the phenylpropanoid metabolic pathway crosstalk with many downstream secondary metabolite pathways such as flavonoid and anthocyanin biosynthesis. It is well known that high temperature stress induces the production of phenolic compounds such as flavonoids and phenylpropanoids for thermotolerance [9]. The key enzyme in the phenylpropanoid metabolism pathway, phenylalanine ammonia-lyase (PAL), was activated during the day in leaves of *R. stricta* (Table 1 and Additional file 7: Figure S4). Activity of PAL in response to heat stress was reported earlier as the main acclimatory response [9] where the enzyme induces the biosynthesis of other phenolics in the pathway. Phenolics, including flavonoids and anthocyanins, were reported earlier as the key secondary metabolites in abiotic stress tolerance [50, 51]. In contrast, peroxidase enzyme was repressed in the phenylpropanoid metabolism pathway indicating the suppression of oxidation of phenolics in apical and mature leaves of *R. stricta* during the day. This action can help reduce detoxification of ROS to maintain cell membrane permeability [24].

The enzyme chalcone synthase, the first enzyme in flavonoid biosynthesis pathway, was activated during the day in leaves of *R. stricta* (Table 1 and Additional file 8: Figure S5). This enzyme is also important in the orchestration of several other pathways, including flavone and flavonol biosynthesis and anthocyanin biosynthesis. Three other enzymes in the flavonoid biosynthesis pathway involved in the synthesis of several important intermediate flavonoids, flavonoid 3′,5′-hydroxylase, flavonoid 3′-monooxygenase and naringenin 3-dioxygenase, were also activated in leaves of *R. stricta* under heat stress. Two other key enzymes, leucocyanidin reductase (LAR) and leucocyanidin oxygenase, were activated in leaves of *R. stricta* towards the production of important phenolics. The first enzyme acts in the formation of proanthocyanidins (PAs), polymers of flavan-3-ol subunits, while the action of the second enzyme is linked directly through many avenues to the anthocyanin biosynthesis pathway (Table 1 and Additional file 9: Figure S6). Earlier reports in grape indicated that increased temperature enhances the production of PAs [52], which act in protecting plants against herbivores and UV radiation during the day [53]. The KEGG analysis in the anthocyanin biosynthesis pathway indicated the activation of only one enzyme, UDP-glucose:anthocyanidin (Table 1 and Additional file 9: Figure S6). This key enzyme catalyzes the first step of the pathway towards the eventual synthesis of many anthocyanins in the cell.

Two light-responsive enzymes in the carotenoid biosynthesis pathway were also regulated in leaves of *R. stricta* (Table 1 and Additional file 10: Figure S7). The first, zeaxanthin epoxidase, was repressed under heat stress, while the second, violaxanthin de-epoxidase, was activated. The two enzymes act as a shuttle for the reversible interconversion of the two carotenoids zeaxanthin and violaxanthin and their activities are light regulated [9]. It is evident that zeaxanthin biosynthesis was enhanced, while violaxanthin biosynthesis was repressed. Zeaxanthin is known for its role in photoprotection in the cells as it also acts to prevent peroxidative damage to the membrane lipids triggered by ROS under abiotic stresses [24, 54].

The pathway of cysteine and methionine metabolism is regulated in mature leaf cells of *R. stricta* under heat stress towards the oversynthesis of methionine (Table 1 and Additional file 11: Figure S8) due to the activation of three enzymes, methionine synthase, tyrosine aminotransferase and aromatic-amino-acid transaminase. Two other enzymes, S-adenosylmethionine synthetase and S-adenosylmethionine decarboxylase, were also activated in both apical and mature leaves towards the depletion of methionine. However, this can be compensated for in mature leaves by the action of the three enzymes indicated earlier for oversynthesis of methionine. Cysteine seems negatively regulated in both apical and mature leaves due to the possible repression of cycteine synthase A/B enzyme in the cell under heat stress. Methionine is a major amino acid in chloroplast small heat shock proteins (sHSPs), which act in plant adaptation to severe heat stress by protecting the process of photosystem II electron transport [55]. Gustavsson et al. [56] also reported that methionine residues in HSP21 mediate protein repair under heat stress.

#### Regulated gene families under heat stress with ≥ 5 fold change

**Fig. 7:**
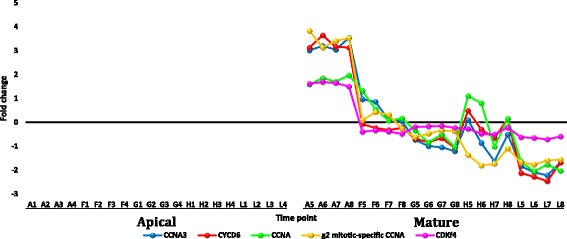
Fold change values for the downregulated genes encoding cyclin in apical (A1-L4) and mature (A5-L8) leaves during the day (A, morning; F-H, midday & L, dusk) in *R. stricta*

**Fig. 8:**
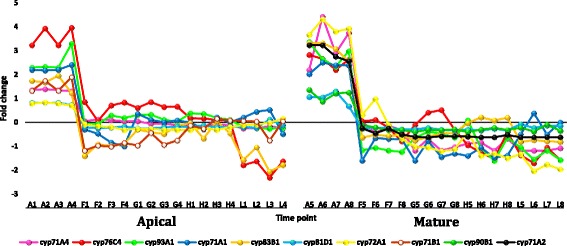
Fold change values for the downregulated genes encoding cytochrome P-450 in apical (A1-L4) and mature (A5-L8) leaves during the day (A, morning; F-H, midday & L, dusk) in *R. stricta*

**Fig. 9:**
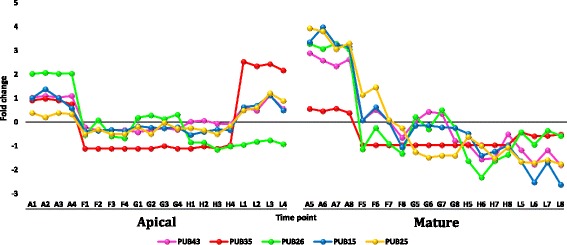
Fold change values for the downregulated genes encoding U-box containing proteins in apical (A1-L4) and mature (A5-L8) leaves during the day (A, morning; F-H, midday & L, dusk) in *R. stricta*

**Fig. 10:**
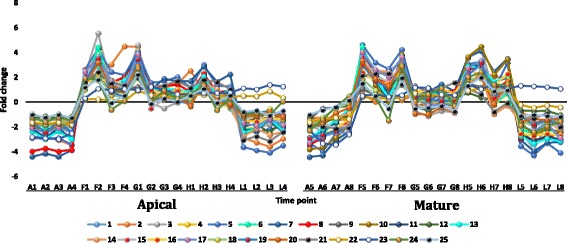
Fold change values for the upregulated genes encoding HSPs in apical (A1-L4) and mature (A5-L8) leaves during the day (A, morning; F-H, midday & L, dusk) in *R. stricta*. Numbers refer to those in Additional file 13: Table S5

**Fig. 11:**
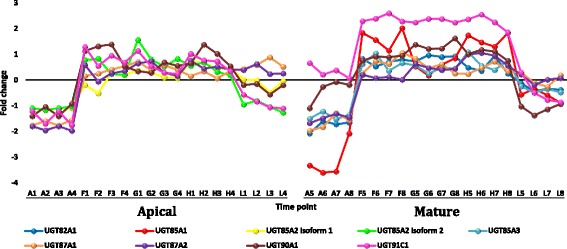
Fold change values for the upregulated genes encoding UDP-glycosyltransferase in apical (A1-L4) and mature (A5-L8) leaves during the day (A, morning; F-H, midday & L, dusk) in *R. stricta*

**Fig. 12:**
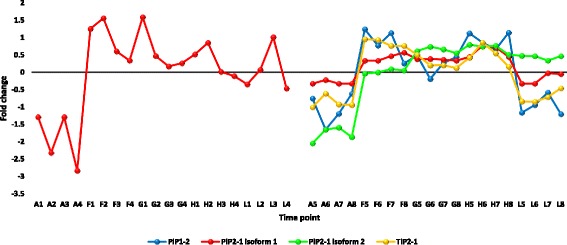
Fold change values for the upregulated genes encoding aquaporins (AQPs) or major intrinsic proteins (MIPs) in apical (A1-L4) and mature (A5-L8) leaves during the day (A, morning; F-H, midday & L, dusk) in *R. stricta*

**Fig. 13:**
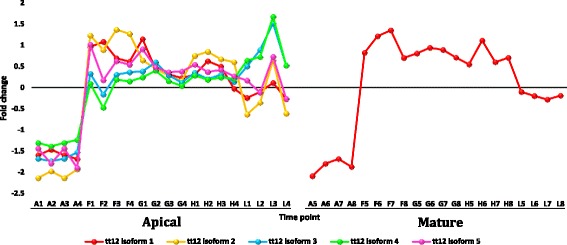
Fold change values for the upregulated genes encoding protein transparent testa 12 (tt12) in apical (A1-L4) and mature (A5-L8) leaves during the day (A, morning; F-H, midday & L, dusk) in *R. stricta*

Transcripts selected from the datasets of apical and mature leaves of *R. stricta* that showed down or upregulation with fold change (FC) of ≥ 5 are shown in Additional file 12: Table S4. Analysis was selectively done for gene families whose members were frequently up or down regulated in leaves of *R. stricta* or those with prior information on their response to heat stress. The selected highly downregulated transcripts at highest midday temperatures in leaves of *R. stricta* included genes encoding cyclin, cytochrome p450/secologanin synthase and U-box containing proteins (Additional file 12: Table S4 and Figs. 7, 8 and 9, respectively). Upregulated, abundant transcripts included genes encoding HSPs/chaperones, UDP-glycosyltransferase, aquaporins and protein transparent testa 12 (Additional file 12: Table S4 and Figs. 10, 11, 12 and 13, respectively). Some upregulated transcripts showed extreme downregulation at dusk, while none of them showed downregulation at midday with no differential regulation among the three time points of the midday (e.g., F, G & H).

Transcripts encoding cyclin proteins A (CCNA), A3 (CCNA3) and D6 (CYCD6) were downregulated only in mature leaves of *R. stricta* (Additional file 12: Table S4). The plant *cyclin* gene family has 10 types (A, B, C, D, H, L, T, U, SDS and J18; Zhang et al. [57]). The A and D types are involved in regulation of cell division during phases S to M and G1 to S, respectively [58]. Thus, it is likely that mature leaf cells of *R. stricta* were arrested at G1-S phases due to heat stress. In agreement with these findings, transcripts encoding cyclin-dependent kinase (CDK) class f4-like, a regulator of cell cycle progression through the binding to cyclin, were also highly downregulated at midday only in mature leaves, while upregulated in apical leaves of *R. stricta* (Additional file 12: Table S4). This should result in prompt inhibition of cell division in mature leaves only, which may be a mechanism of tolerance by avoiding or escaping heat stress. Based on these results, the stress avoidance mechanism is not likely applicable to apical leaves of *R. stricta* whose major process is cell division. Recent studies indicated the indirect role of CDKs in plant tolerance to heat stress via a sophisticated mechanism of stress avoidance [59].

Highly downregulated transcripts encoding cytochrome P450 (cyt P450) in response to heat stress were identified in leaves of *R. stricta* (Additional file 12: Table S4). This involved 10 genes belonging to seven gene families, *cyp71A1, cyp71A2, cyp71A4, cyp71B1, cyp72A1* (encoding secologanin synthase)*, cyp76C4, cyp81D1, cyp83B1, cyp90b1 and cyp93A1*. There are no previous reports implicating cyt P450 genes in thermotolerance, however, Larkindale and Vierling [60] indicated the downregulation of 18 different *cyp* genes in *Arabidopsis* under high temperature stress. Other reports indicated the involvement of some of these genes in other biological processes. For example, *cyp71A1* and *cyp72A1* (encoding secologanin synthase) genes are involved in the synthesis of indole alkaloid secologanin, which is important in mevalonate pathway for the production of the two anticancer bisindole alkaloids vinblastine and vincristine [61]. *cyp83B1* is involved in the biosynthesis of glucosinolates, which have anticancer and flavoring functions [62]. *cyp71A4* is involved in the defense response to pathogen attacks [63]. In conclusion, the high levels of downregulation of a large number of *cyp* genes in response to heat stress in leaves of *R. stricta* is not fully understood.

Large numbers of upregulated, abundant transcripts of genes encoding HSPs and chaperones (or chaperonin) were detected in leaves of *R. stricta* (Additional file 12: Table S4 & Additional file 13: Table S5). These genes are frequently reported as being involved in plant thermotolerance (e.g., Hu et al. [58]). HSPs are protective proteins acting as molecular chaperones that prevent protein misfolding and aggregation or denaturation during heat stress [64]. Recent reports indicated that ATP-independent chaperones act with sHSPs as “holdases” to suppress the aggregation of proteins and delay their folding under heat stress [65]. ATP-independent chaperones also assist with protein refolding under heat stress to recover original protein structures [66]. There are two major groups of HSPs, high molecular mass or HMM-HSPs ranging from 60 to 100 KDa and small sHSPs ranging from 15 to 30 kDa [64]. Genes within these two groups were classified into five gene families based on intracellular localization. Classes I and II are cytosolic, while classes III, IV and V are localized in the chloroplast, mitochondrion or endoplasmic reticulum [64, 67]. In the present study, upregulated, abundant transcripts encoding HMM-HSPs in leaves of *R. stricta* during the day were cytosolic of class I, while those encoding sHSPs were either cytosolic of class II or chloroplastic of classes III or IV (Additional file 12: Table S4). Transgenics of different plant species with *hsp* genes, especially chloroplastic (CP), showed enhanced tolerance to heat stress [68–72] mainly via the protection of Photosystem II (PSIIHSP) [55]. The amount of CP-sHSPs in per unit protein correlated positively with the level of tolerance in *Chenopodium album* and *Lycopersicum esculentum* [73]. Upregulated, abundant transcripts encoding UDP-glycosyltransferase also occurred in leaves of *R. stricta* (Additional file 12: Table S4). They involve eight genes belonging to five gene families, *ugt82A1*, *ugt85A1*, *ugt85A2*, *ugt85A3*, *ugt87A1*, *ugt87A2*, *ugt90A1* and *ugt91C1*. The enzyme is a key player in the quality control mechanism for newly synthesized glycoproteins in the endoplasmic reticulum (ER). This organelle hosts the synthesis/folding of proteins secreted extracellularly or delivered to endomembrane system [74]. Quality control includes the calnexin (CNX)/calreticulin (CRT) cycle, which involves lectin-chaperones retaining N-glycosylated proteins at the ER while they undergo the folding process. UDP-glucosyltransferase acts on unglycosylated proteins during folding and catalyzes reglucosylation to allow protein binding to CNX/CRT and retention in the ER to continue the folding process [75]. In contrast, several highly downregulated transcripts encoding U-box containing proteins occurred in leaves of *R. stricta*, such as E3 ubiquitin-protein ligase atl42. The genes include *pub15*, *25*, *26*, *35* and *43* (Additional file 12: Table S4). The U-box protein family represents a class of E3 enzymes (e.g., CHIP) acting as a degradatory co-chaperone of HSP70 and HSP90 [76]. This is another mechanism of protein quality control under heat stress where the interaction of HSPs with co-chaperones that have either folding or degradatory activity determines the fate of HSP proteins. In transgenic *Arabidopsis* with overexpressed *pub22* and *23* genes, two homologous U-box E3 ubiquitin ligases caused hypersensitivity to drought stress [77, 78]. In contrast, loss-of-function of these two genes resulted in the recovery of drought-tolerant plants. In our analysis, the high levels of downregulation of *pub23* as well as the other genes encoding U-box containing proteins in leaves of *R. stricta* indicates that the degradatory activity of these enzymes on target proteins under heat stress is unlikely (Additional file 12: Table S4). In conclusion, the occurrence of upregulated, abundant transcripts encoding HSPs, chaperones and UDP-glucosyltransferase and downregulated transcripts encoding U-box containing proteins likely contribute to thermotolerance in leaf cells of *R. stricta* by maintaining proper protein folding and preventing protein degradation.

Upregulated, abundant transcripts encoding aquaporins (AQPs) or major intrinsic proteins (MIPs) also occurred in leaves of *R. stricta* (Additional file 12: Table S4). The main types of MIPs include plasma membrane intrinsic proteins (PIPs) and tonoplast intrinsic proteins (TIPs). In *R. stricta*, three MIP genes, *pip2-1*, *pip1-2* and *tip2-1*, were detected and may be involved in thermotolerance in leaves. AQPs are classes of membrane proteins that facilitate water diffusion across cell membranes during the response/tolerance to adverse environmental stimuli [79, 80] including heat stress [81]. They are also involved in the opening and closing of cellular gates and in the physiology of water balance and water use efficiency under abiotic stresses [82]. We propose that the upregulation of the three genes encoding AQPs may contribute to thermotolerance of *R. stricta* leaves. Finally, upregulated, abundant transcripts encoding protein transparent testa 12 (tt12) occurred in leaves of *R. stricta* (Additional file 12: Table S4). Debeaujon et al. [83] indicated that flavonoids were sequestered in seed coat endothelium due to the action of the *tt12* gene but our results suggest that upregulation of this gene in leaves is a response to heat stress. This conclusion complements the results of KEGG analysis of flavonoid biosynthesis under heat stress because this process is likely followed by sequestering of accumulated flavonoids in the vacuole of leaf cells. As indicated earlier, flavonoids are key secondary metabolites in abiotic stress tolerance [50].

#### Transcription factors co-expressed with *Hsp* or chaperone genes

**Fig. 14:**
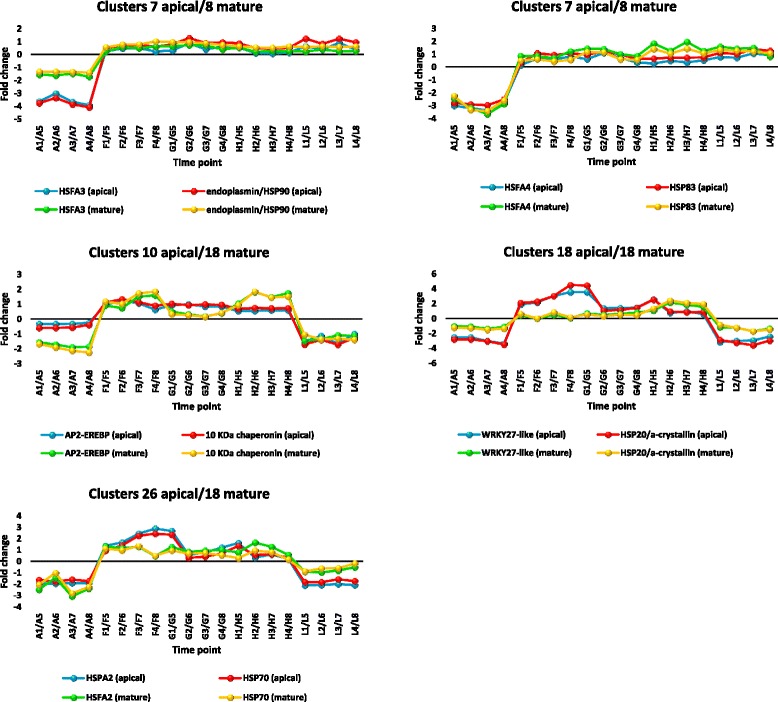
Selected transcription factors with very similar co-expression patterns with HSP or chaperone genes in apical (A1-L4) and mature leaves (A5-L8) of *R. stricta* at different time points of the day (A, morning; F-H, midday & L, dusk). Expression patterns involved upregulation starting midday (clusters 7 (apical)/8 (mature)) and upregulation at midday (clusters 10, 18 or 26 (apical)/18 (mature)). Transcript fold change refers to that described in Additional file 14: Table S6

Co-expression analysis was done to investigate the transcription factors that may regulate genes encoding HSPs or chaperones in apical and mature leaves of *R. stricta* with special emphasis on those upregulated at the sampling times with the highest temperatures at midday (Additional file 13: Table S5). The clusters of DE transcripts selected for co-expression analysis in either leaf type were those with the highest number of the target genes (Additional file 14: Table S6). They are clusters 4, 7, 10, 18 and 26 for apical leaves (Additional file 1: Table S1), and 8 and 18 for mature leaves (Additional file 2: Table S2). Selected TFs co-expressed with genes encoding HSPs and chaperones are shown in Additional file 14: Table S6. These TFs are known for their response to abiotic stresses, including HSFAs, AP2/ERF (or AP2-EREBP), WRKY, bHLH, zinc finger and MYB (Additional file 14: Table S6). Co-expressed *hsp* genes encode HSP10, HSP20, HSP60, HSP70, HSP83 and HSP90. The results indicated several highly concordant co-expressions of TFs and genes encoding either HSPs or chaperones during the day. Five pairs of these co-expressing genes in both apical and mature leaves were selected for further analysis (Fig. 14). The expression pattern of each gene pair was the same in both types of leaves. The highly similar co-expression across the day in each of the five gene pairs is unlikely to be coincidental. Expression patterns of these five pairs included upregulation starting midday (clusters 7 of apical leaves and 8 of mature leaves) and upregulation at midday (clusters 10, 18 and 26 of apical leaves and 18 of mature leaves). The first pattern indicated co-expression of genes encoding HSFA3 and endoplasmin/HSP90, and co-expression of genes encoding HSFA4 and HSP83 (Additional file 14: Table S6 and Fig. 14). The second expression pattern involved co-expression of genes encoding AP2/ERF (or AP2-EREBP) and 10 KDa chaperonin (clusters 10 of apical leaves and 18 of mature leaves, Additional file 14: Table S6 and Fig. 14), co-expression of genes encoding WRKY27-like and HSP20/α-crystallin (clusters 18 of both apical and mature leaves, Additional file 14: Table S6 and Fig. 14) and co-expression of genes encoding HSFA2 and HSP70 (clusters 26 of apical leaves and 18 of mature leaves, Additional file 14: Table S6 and Fig. 14).

The expression of *hsp* genes in response to various stimuli is regulated by HSFs [84]. Our results indicated the co-expression of the upregulated, abundant transcripts encoding HSP70, HSP90 and HSP83 with the upregulated, abundant transcripts encoding HSFA2, HSFA3 and HSFA4, respectively (Additional file 14: Table S6 and Fig. 14). There are three clases of HSFs in *Arabidopsis*, A, B and C [85]. Only class HSF A harbors an AHA motif essential for transcription activation of *hsp* genes [86, 87]. We observed five upregulated, abundant HSF transcripts in leaves of *R. sticta* that encode HSFA1, HSFA2, HSFA3, HSFA4 and HSFB1 (Additional file 12: Table S4). Earlier reports on the gene encoding HSFA1 indicated that it functions as a master regulator of early heat stress response in tomato [88] and plays an important role in the induction of several *hsp* genes in *Arabidopsis* [89]. The gene encoding HSFA2 is a heat-inducible transactivator sustaining the expression of *hsp* genes and promoting an extended duration of acquired thermotolerance in *Arabidopsis* [90]. Disruption of the gene encoding HSFA2 resulted in a reduction in expression of *shsp* genes *Hsp18.1*, *Hsp25.3-P* and *Hsa32* under stress, while overexpression resulted in enhanced thermotolerance [91]. However, our results indicated the co-expression of the transcript encoding HSFA2 with a *HMM-hsp* transcript encoding HSP70 in leaves of *R. stricta*. As for the gene encoding HSFA3, Yoshida et al. [92] found that it is highly upregulated under heat stress in transgenic plants overexpressing *dreb2A* gene. This indicates that *dreb2A* acts upstream of the gene encoding HSFA3*.* In turn, *hsfA3* was reported to regulate expression of many heat-inducible genes acting downstream in the transcriptional cascade as this gene acts as a potent activator on the *hsp* gene promoters [93]. *hsfA3*-knockout mutant lines of *Arabidopsis* showed reduced levels of *hsp101* and *shsps* under heat stress. We have no evidence for the expression of *hsp101* in leaves of *R. stricta*. Rather, our results indicated the co-expression of genes encoding HSFA3 and the HMM-*hsp* gene encoding a HSP90. The gene encoding HSFA4 in leaves of *R. stricta* co-expressed with the *hsp83* gene (Fig. 14). This TF was reported recently to enhance transient transactivation of *hsp17.6A* transcription under heat stress [94]. It was also reported to confer tolerance to salt and oxidative stresses. Our results contradict those of Pérez-Salamó et al. [94] as this TF co-expressed with a gene encoding a HMM-HSP rather than the sHSP17.6A protein. In general, we can conclude that the HSF machinery in *R. stricta* basically acts on *HMM-hsp* genes. None of the detected HSFAs involved in co-expression in the present study drives expression of *shsp* genes as reported in other plant species, such as *Arabidopsis*.

Our results indicate that one upregulated, abundant transcript encoding AP2-EREBP was co-expressed with an upregulated, abundant transcript encoding 10 KDa chaperonin (Additional file 14: Table S6 and Fig. 14) in leaves of *R. stricta*. A large number of transcripts encoding AP2/ERFs or AP2-EREBP were also upregulated during the day in *R. stricta* leaves (Additional file 14: Table S6). They include genes encoding AP2-ERF17, AP2-ERF34, AP2-ERF98, AP2-ERF109 and AP2-EREBP (AT2G41710 in *Arabidopsis*). Two more genes encoding AP2-ERF were differentially expressed in *R. stricta* including the upregulated gene encoding AP2-ERF23 and the downregulated gene encoding AP2-ERF10 (Additional file 2: Table S2 & Additional file 6: Table S3)*.* Yates et al. [31] indicated that the gene family encoding AP2-EREBP showed a decrease in expression at midday. These results apply in the present study to genes encoding AP2-ERF04, AP2-ERF5, AP2-ERF12, AP2-ERF13, AP2-ERF1b, AP2-ERFabr1 and AP2-ERFwin1 (Additional file 2: Table S2 & Additional file 6: Table S3)*.* In general, ERFs were thought to act upstream of HSFs in the response to heat stress especially at night [95], but no previous reports indicate that ERF drives expression of genes encoding chaperonin. AP2/ERFs contain at least one DNA binding domain, AP2 domain [96]. They were induced by biotic and abiotic stresses including drought, heat, salt, osmotic, wounding, etc. [97]. The most extensively studied ERFs in abiotic stress responses were the DREB proteins. Among them, DREB2A in *Arabidopsis* was induced by heat in an ABA-ethylene-independent manner and plants overexpressing it exhibited improved tolerance to heat stress [98].

We also observed the co-expression of the upregulated genes encoding WRKY27 and HSP20/α-crystallin in apical and mature leaves of *R. stricta* (Fig. 14). This TF was reported in *Arabidopsis* to influence wilt disease symptom caused by *Ralstonia solanacearum* [99], but there are no reports supporting its involvement in heat stress. Three other genes encoding WRKY28, WRKY35 and WRKY56 were also upregulated in leaves of *R. stricta* but with lower rates. Previous reports indicated that one WRKY transcription factor and four HSFs were induced by heat stress in switchgrass [100]. Yates et al. [31] indicated that the gene family encoding WRKY showed decreased expression at midday. We confirmed these results for genes encoding WRKY6, WRKY7, WRKY12, WRKY33, WRKY40, WRKY44, WRKY46, WRKY48, WRKY49, WRKY50, WRKY53 and WRKY55 (Additional file 2: Table S2 & Additional file 6: Table S3). There are reports indicating that the constitutive expression of genes encoding WRKY25, WRKY26 or WRKY33 enhanced tolerance to heat stress [101]. No previous reports have indicated the direct involvement of WRKY in driving *hsp* genes, including the *shsp20*. Calmodulin-dependent protein kinases were induced in switchgrass by heat stress [100]. A number of transcripts encoding calmodulin-dependent protein kinase were detected in leaves of *R. stricta* at different rates across the day (Additional file 2: Table S2 & Additional file 6: Table S3). Investigations of the role of calmodulin in cells of *Arabidopsis* indicated that it was required for heat stress signaling and can serve as an activator of WRKY39 and a number of HSFs [102]. It seems that this is not the case in leaves of *R. stricta*.

## Conclusion

In general, we conclude that enzymes in several pathways are interacting in the biosynthesis of soluble sugars, polyols, secondary metabolites, phenolics and methionine. Genes encoding these enzymes can be primary contributors to thermotolerance in the wild plant species *R. stricta*. A number of heat-responsive genes are regulated under heat stress and can add to the thermotolerance of this plant species. Our analyses also indicate the relationship of individual genes and transcription factors during heat stress. Overall, our results contribute to the knowledge of the regulatory mechanisms underlying heat stress response at the molecular level in *R. stricta*. This is particularly important because the native habitat of this species is extremely hot, making it an excellent system for examining plant responses to heat stress. In the future, manipulating the expression of genes affecting protein folding and degradation, such as those encoding HSPs, chaperones, UDP-glucosyltransferase and U-box, holds great potential for improving thermotolerance of economically important crop plants.

## Methods

### Plant sampling and RNA-Seq data

Apical and mature leaves of *R. stricta* were collected from the Bahrah region, Jeddah, Saudi Arabia. The voucher specimen was deposited in the Department of Biological Sciences Herbarium at King Abdulaziz University (Number 1150/M/75 collected by N. Baeshen, M. Baeshen and J. Sabir). Samples of the two leaf types were taken in four replicates at seven time points of the day from which the following five time points of the day were selected; morning (A, 07:10), midday (F, 13:25; G, 14:05 & H, 14:30) and dusk (L, 18:27). The temperatures at the five time points were 27.4 (A), 42.4 (F), 42.2 (G), 40.0 (H) and 33.5 °C (L). The total number of samples across the two leaf types was 40 (2 leaf types X 5 time points X 4 replicates). The morning and dusk samples were taken immediately after sunrise and immediately before sunset, respectively. The first time point is considered as the control condition for heat stress, while time points F-H were collectively designated midday. RNA samples were isolated [31] and sequenced by Genome Enterprise Limited (GEL) at The Genome Analysis Centre (Norwich, UK) and the resulting raw reads from the RNA-Seq data were deposited in the short read archive (SRA) of the NCBI (study SRP028238).

### Bioinformatics analysis

Relative abundance of reads was calculated by RSEM v1.1.6 with the *R. stricta* nuclear genome [103] used as a reference. By default, RSEM uses the Bowtie aligner [104] (Bowtie v0.12.1) to map the reads against the transcripts. Expected read counts were used as input to DE analysis by EdgeR (version 3.0.0, R version 2.1.5). The median of these values was used as the common dispersion factor for DE analyses. DE transcripts were annotated and KEGG pathway analyses were performed using Blast2GO software [105] (version 2.3.5, https://www.blast2go.com). Further, Blastx was performed for DE transcripts in selected clusters against the NCBI non-redundant protein database with an E value cut-off of 1e^−5^. HMMER v3.1b2 was used to identify protein domains common in TFs to detect TF genes possibly involved in regulating expression of DE transcripts encoding HSPs and chaperones in the selected clusters. Differential expression data were introduced in fold change of transcript levels of either leaf type under heat stress at midday as compared to those of the control condition at the morning.

### Validation of RNA-Seq datasets

An experiment was conducted to validate the RNA-Seq data for selected genes whose expression pattern in apical leaves was similar to that in mature leaves, and whose expression rate was mostly consistent within the four replicates of each time point. Expression patterns suitable for the validation experiment across the two types of leaves included upregulation starting at midday and gradual downregulation across the day in which 10 transcripts were randomly selected and *actin* was used as the unregulated housekeeping gene (Additional file 6: Table S3). Three out of the four replicates of RNA samples for the original RNA-Seq study [31] were utilized in the experiment to validate expression patterns of the selected genes across the different time points of the day (A, morning; F-H, midday; L, dusk) via semi-quantitative RT-PCR. Primers were designed using Netprimer software (http://www.premierbiosoft.com/netprimer/index.html) with the following criteria: length ~20 bases, GC content ~50%, minimal secondary structure, comparable annealing temperatures (55 °C) of the primer pairs, and PCR products of ~350–450 bp (Additional file 6: Table S3).

## Additional files

Additional file 1: Table S1. Fold change values of assembled transcripts of *R. stricta* SRA database in different clusters in the apical leaves (A1-L4) at different time points of the day (A, morning; F-H, midday & L, dusk). (XLSX 775 kb)
Additional file 2: Table S2. Fold change values of assembled transcripts of *R. stricta* SRA database in different clusters in the mature leaves (A5-L8) at different time points of the day (A, morning; F-H, midday & L, dusk). (XLSX 1523 kb)
Additional file 3: Figure S1. Clusters of assembled transcripts of *R. stricta* in apical leaves (A1-L4) at different time points of the day (A, morning; F-H, midday & L, dusk). Grey lines indicate expression patterns of individual transcripts in a given cluster. Blue lines indicate overall expression pattern across different transcripts of a given cluster. (PDF 223 kb)
Additional file 4: Figure S2. Clusters of assembled transcripts of *R. stricta* SRA in mature leaves (A5-L8) at different time points of the day (A, morning; F-H, midday & L, dusk). Grey lines indicate expression patterns of individual transcripts in a given cluster. Blue lines indicate overall expression pattern across different transcripts of a given cluster. (PDF 397 kb)
Additional file 5: Figure S3. Semi-quantitative RT-PCR and profiles of fold change values resulting from RNA-Seq analysis for selected upregulated genes starting midday (1–5) and gradually downregulated genes (6-10) used for validating RNA-Seq data of apical (A1-L4) and mature (A5-L7) leaves of *R. stricta* collected at different time points of the day. Serial numbers 1–10 refer to genes described in Additional file 6: Table S3. The “*actin*” gene was used as the unregulated house-keeping gene. (DOCX 9867 kb)
Additional file 6: Table S3. Fold change values of transcripts in two expression patterns (e.g., upregulation starting midday and gradual downregulation) in the apical (A1-L4) and mature (A5-L8) leaves of *R. stricta* at different time points of the day (A, morning; F-H, midday & L, dusk) selected for validation experiment along with primer information for semi-quantitative RT-PCR. The “actin” was used as the unregulated house-keeping gene. (XLSX 35 kb)
Additional file 7: Figure S4. Enzymes in the phenylpropanoid metabolic pathway in apical and mature leaves responded differentially to changing environment at two time points (morning, A and midday, G). Upregulated (activated) in apical leaves (blue), upregulated in mature leaves (red), downregulated (repressed) in apical leaves (orange box), downregulated in mature leaves (green box). (DOCX 174 kb)
Additional file 8: Figure S5. Enzymes in the flavonoid metabolic pathway in apical and mature leaves responded differentially to changing environments at two time points (morning, A and midday, G). Upregulated (activated) in apical leaves (blue), upregulated in mature leaves (red). (DOCX 193 kb)
Additional file 9: Figure S6. Enzymes in the anthocyanin metabolic pathway in apical and mature leaves responded differentially to changing environment at two time points (morning, A and midday, G). Upregulated (activated) in apical leaves (blue), upregulated in mature leaves (red). (DOCX 210 kb)
Additional file 10: Figure S7. Enzymes in the carotenoid metabolic pathway in apical and mature leaves responded differentially to changing environment at two time points (morning, A and midday, G). Upregulated (activated) in apical leaves (blue), upregulated in mature leaves (red), downregulated (repressed) in apical leaves (orange box), downregulated in mature leaves (green box). (DOCX 242 kb)
Additional file 11: Figure S8. Enzymes in the cysteine and methionine metabolic pathways in apical and mature leaves responded differentially to changing environment at two time points (morning, A and midday, G). Upregulated (activated) in apical leaves (blue), upregulated in mature leaves (red), downregulated (repressed) in apical leaves (orange box), downregulated in mature leaves (green box). (DOCX 201 kb)
Additional file 12: Table S4. Comparative differential expression of genes of *R. stricta* transcriptomes of apical (a) and mature (b) leaves with fold change of ≥ 5 in different time points (A, morning; F-H, midday & L, dusk). Blue box = upregulation, orange box = downregulation. Numbers between parentheses represent no. analogs of a given gene. Green boxes indicate genes selected for further analysis. (DOCX 80 kb)
Additional file 13: Table S5. Fold change values of selected upregulated genes encoding HSPs in apical (A1-L4) and mature (A5-L8) leaves during the day (A, morning; F-H, midday & L, dusk) in *R. stricta* (XLSX 25 kb)
Additional file 14: Table S6. Selected differentially expressed genes in selected clusters of apical (a, A1-L4) and mature (b, A5-L8) leaves indicating the TFs co-expressed with Hsp and chaperone genes during the day (A, morning; F-H, midday & L, dusk) in *R. stricta*. (XLSX 42 kb)

## Abbreviations

AQP: Aquaporin
bHLH: Basic helix-loop-helix
CDK: Cyclin-dependent kinase
CNX: Calnexin
CRT: Calreticulin
Cyt 450: Cytochromome P450
DE: Differential expression
ER: Endoplasmic reticulum
FC: Fold change
HB: Homeobox domain
HSP: Heat shock protein
KDa: Kilodalton
KEGG: Kyoto encyclopedia of genes and genomes
LAR: Leucocyanidin reductase
MIP: Major intrinsic protein
PA: Proanthocyanidin
PAL: Phenylalanine ammonia-lyase
PIP: Plasma membrane intrinsic protein
PSII: Photosystem II
ROS: Reactive oxygen species
sHSP: Small heat shock protein
SRA: Short read archive
TF: Transcription factor
TIP: Tonoplast intrinsic protein
