# Supplementary material for: Analysis of transcriptional response to heat stress in Rhazya stricta
Source: BMC Plant Biol. 2016 Nov 14;16:252. doi: 10.1186/s12870-016-0938-6 (PMC5109689; doi:10.1186/s12870-016-0938-6)

subcluster_10_log2_medianCentered_fpkm.matrix, 61 tra

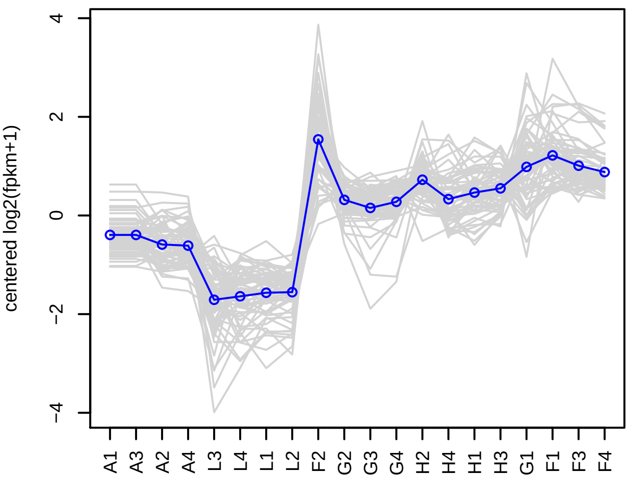

subcluster_11_log2_medianCentered_fpkm.matrix, 32 tra

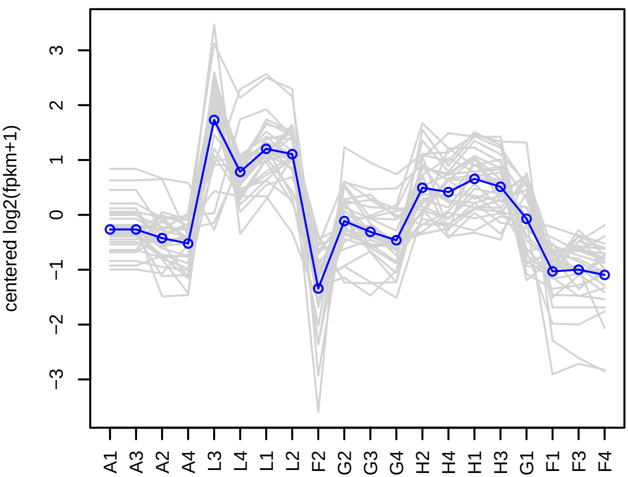

subcluster_12_log2_medianCentered_fpkm.matrix, 158 tra

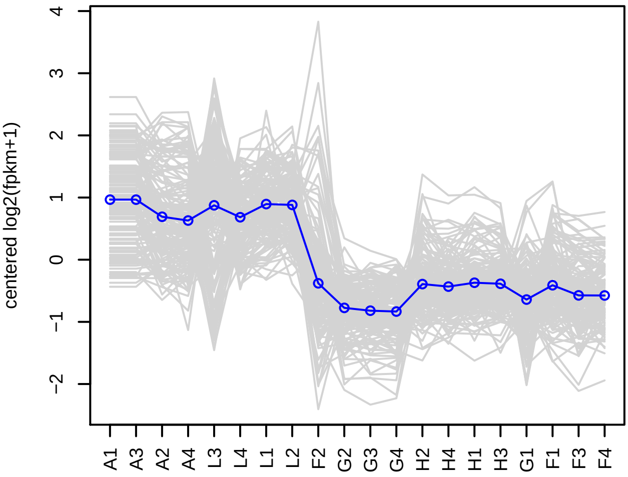

subcluster_13_log2_medianCentered_fpkm.matrix, 34 tra

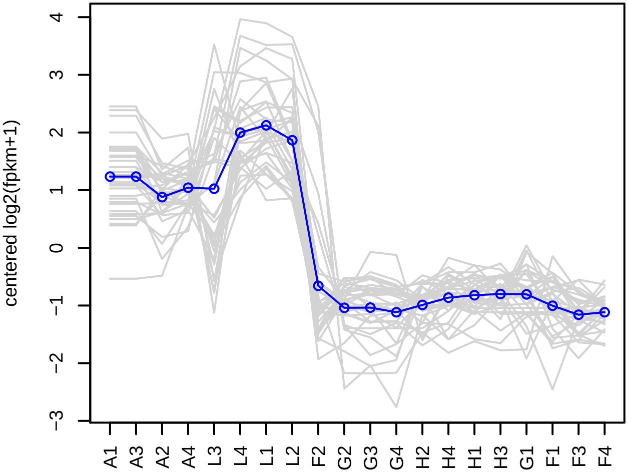

subcluster_14_log2_medianCentered_fpkm.matrix, 129 tra

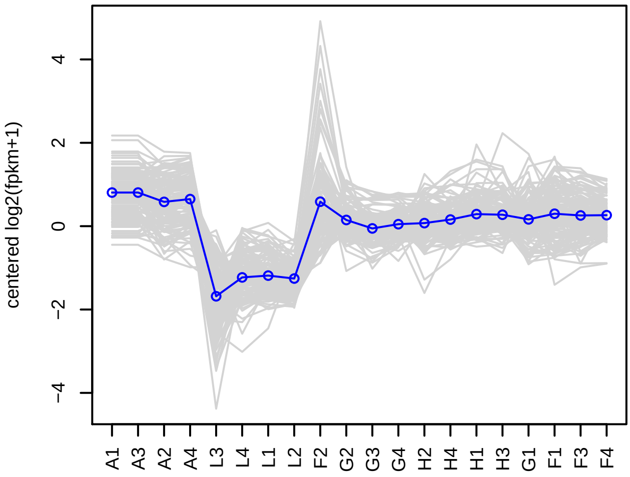

subcluster_15_log2_medianCentered_fpkm.matrix, 10 tra

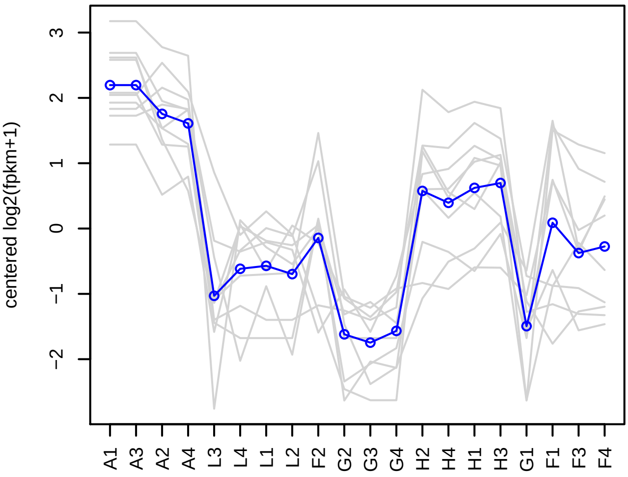

subcluster_16_log2_medianCentered_fpkm.matrix, 11 tra

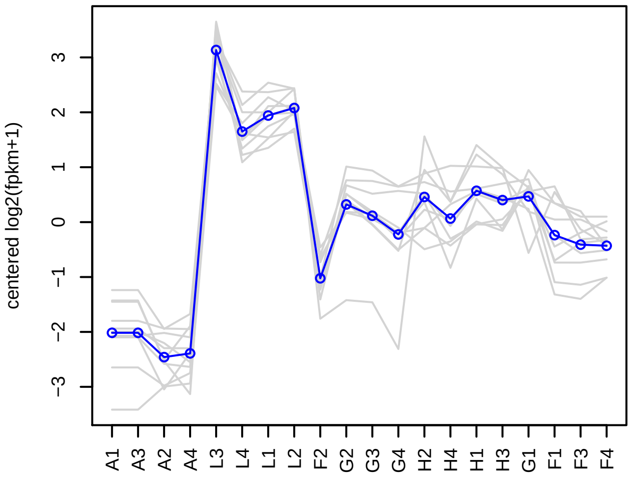

subcluster_17_log2_medianCentered_fpkm.matrix, 27 tra

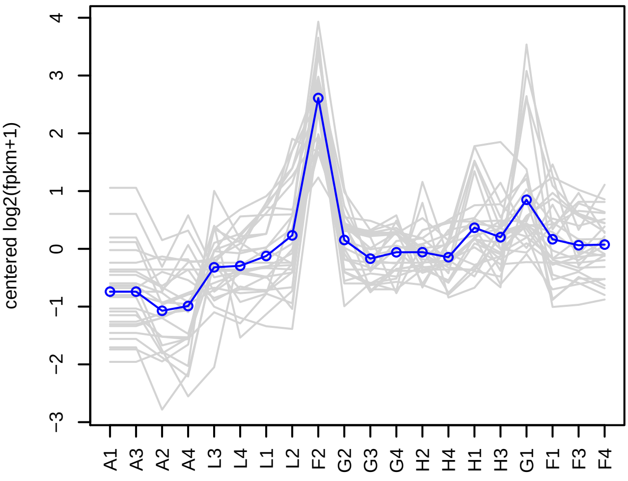

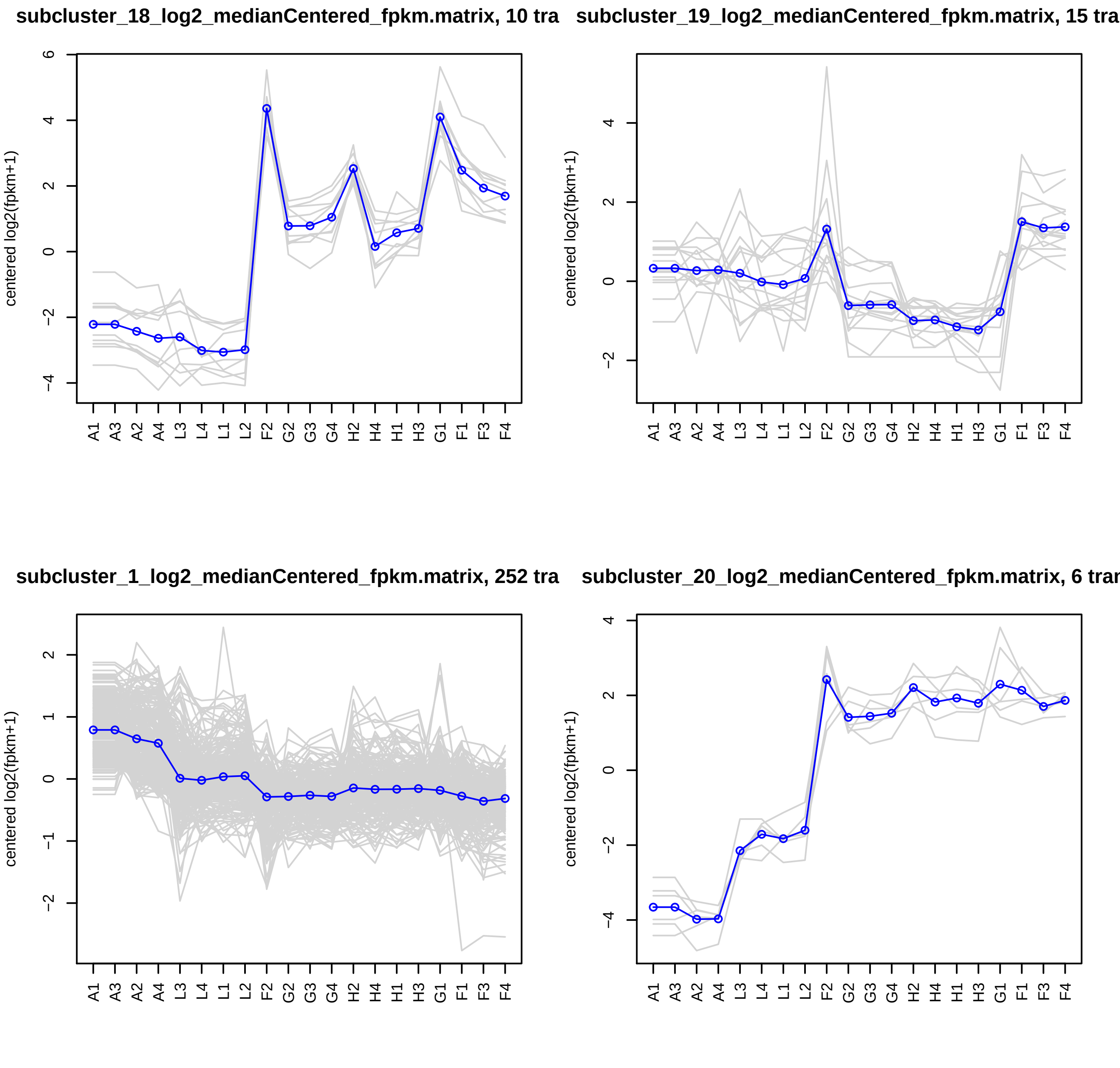

subcluster_18_log2_medianCentered_fpkm.matrix, 10 tra
subcluster_19_log2_medianCentered_fpkm.matrix, 15 tra
subcluster_1_log2_medianCentered_fpkm.matrix, 252 tra
subcluster_20_log2_medianCentered_fpkm.matrix, 6 tran
centered log2(fpkm+1)
A1 A3 A2 A4 L3 L4 L1 L2 F2 G2 G3 G4 H2 H4 H1 H3 G1 F1 F3 F4

subcluster_21_log2_medianCentered_fpkm.matrix, 10 tra

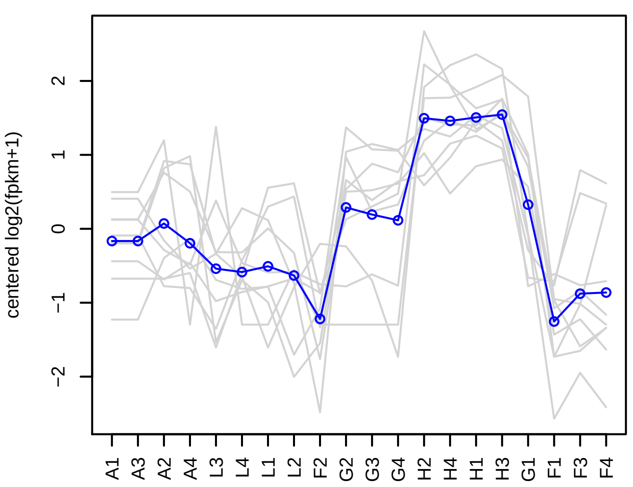

subcluster_22_log2_medianCentered_fpkm.matrix, 20 tra

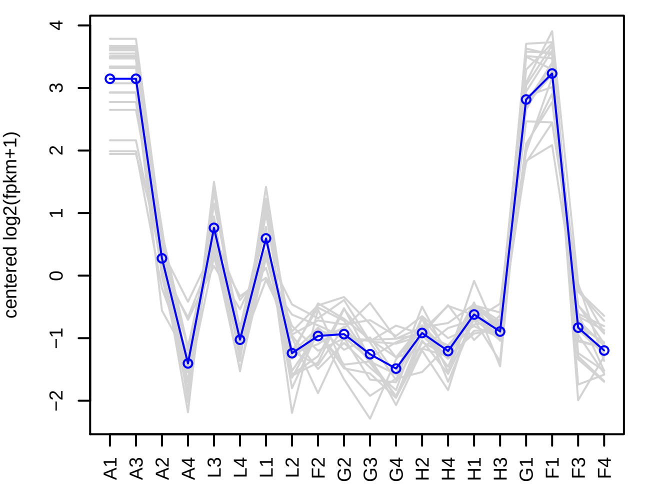

subcluster_23_log2_medianCentered_fpkm.matrix, 17 tra

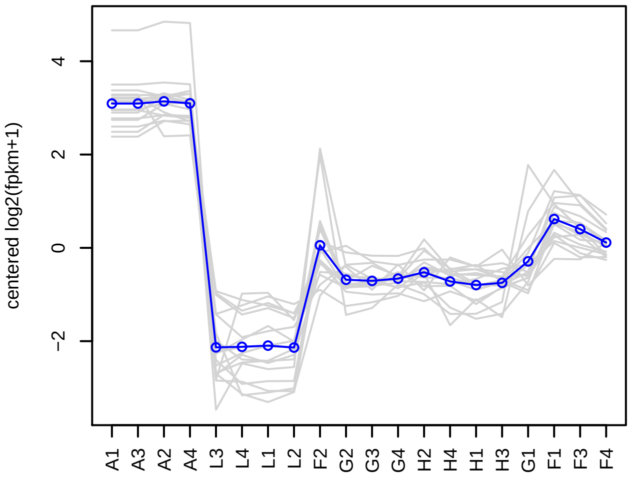

subcluster_24_log2_medianCentered_fpkm.matrix, 7 trar

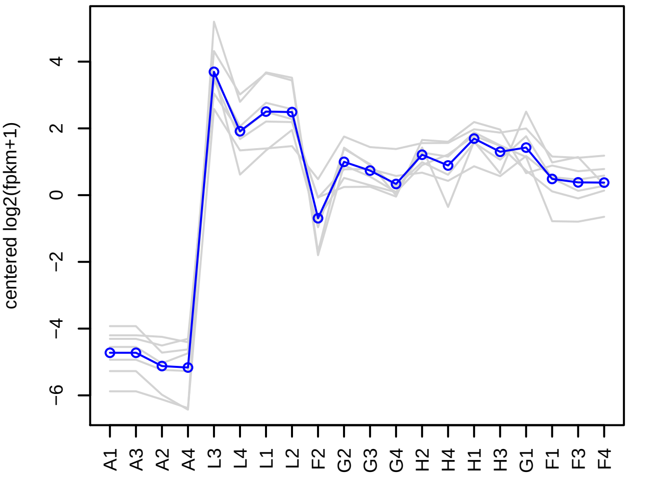

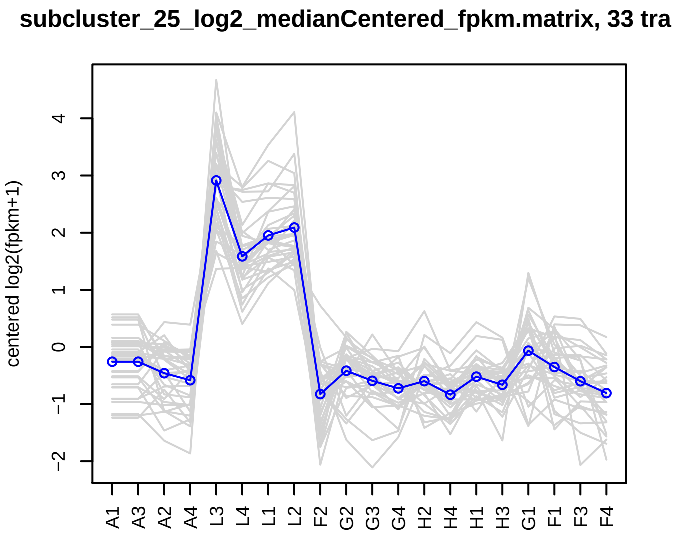
subcluster_25_log2_medianCentered_fpkm.matrix, 33 tra
centered log2(fpkm+1)
A1 A3 A2 A4 L3 L4 L1 L2 F2 G2 G3 G4 H2 H4 H1 H3 G1 F1 F3 F4

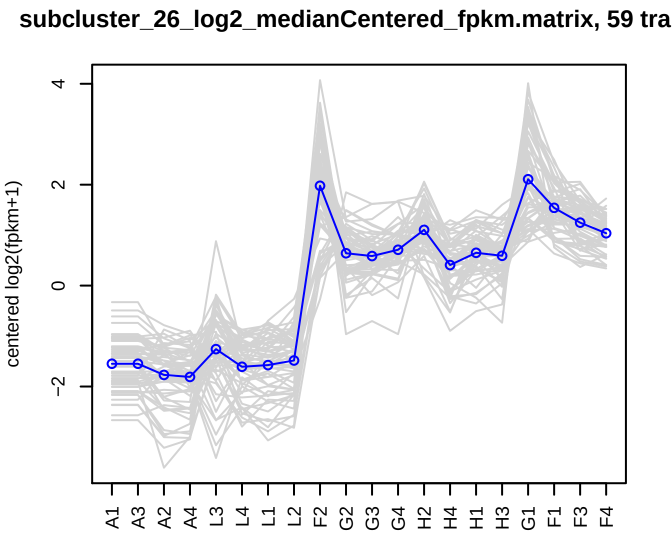
subcluster_26_log2_medianCentered_fpkm.matrix, 59 tra
centered log2(fpkm+1)
A1 A3 A2 A4 L3 L4 L1 L2 F2 G2 G3 G4 H2 H4 H1 H3 G1 F1 F3 F4

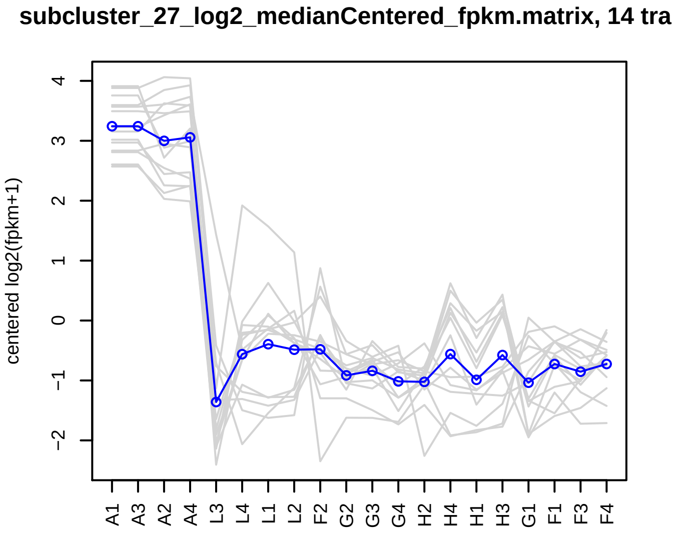
subcluster_27_log2_medianCentered_fpkm.matrix, 14 tra
centered log2(fpkm+1)
A1 A3 A2 A4 L3 L4 L1 L2 F2 G2 G3 G4 H2 H4 H1 H3 G1 F1 F3 F4

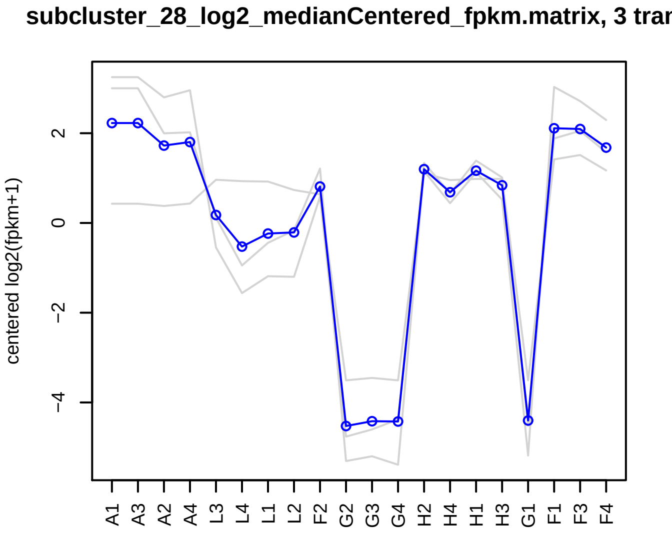
subcluster_28_log2_medianCentered_fpkm.matrix, 3 trar
centered log2(fpkm+1)
A1 A3 A2 A4 L3 L4 L1 L2 F2 G2 G3 G4 H2 H4 H1 H3 G1 F1 F3 F4

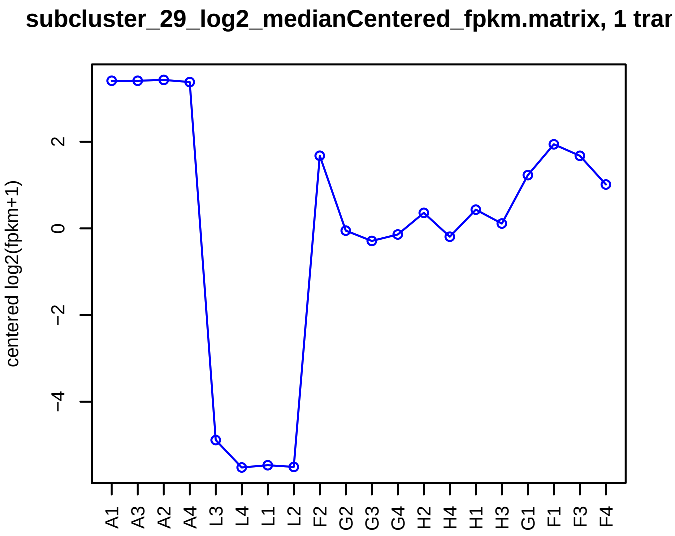
subcluster_29_log2_medianCentered_fpkm.matrix, 1 trar
centered log2(fpkm+1)
2
0
−2
−4
A1 A3 A2 A4 L3 L4 L1 L2 F2 G2 G3 G4 H2 H4 H1 H3 G1 F1 F3 F4

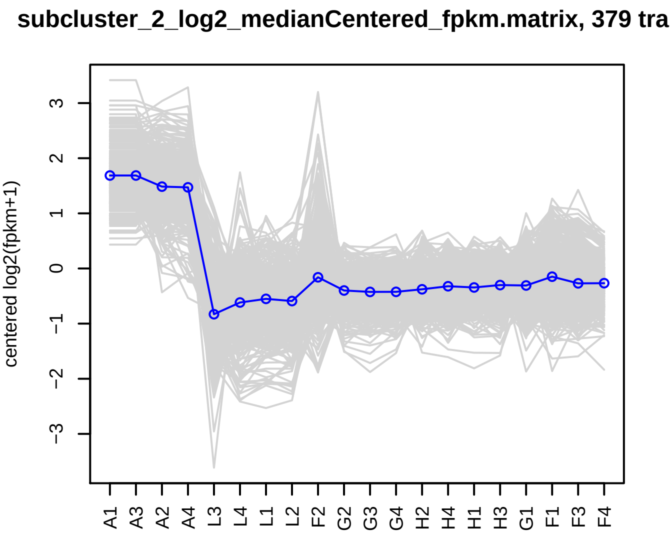
subcluster_2_log2_medianCentered_fpkm.matrix, 379 tra
centered log2(fpkm+1)
3
2
1
0
−1
−2
−3
A1 A3 A2 A4 L3 L4 L1 L2 F2 G2 G3 G4 H2 H4 H1 H3 G1 F1 F3 F4

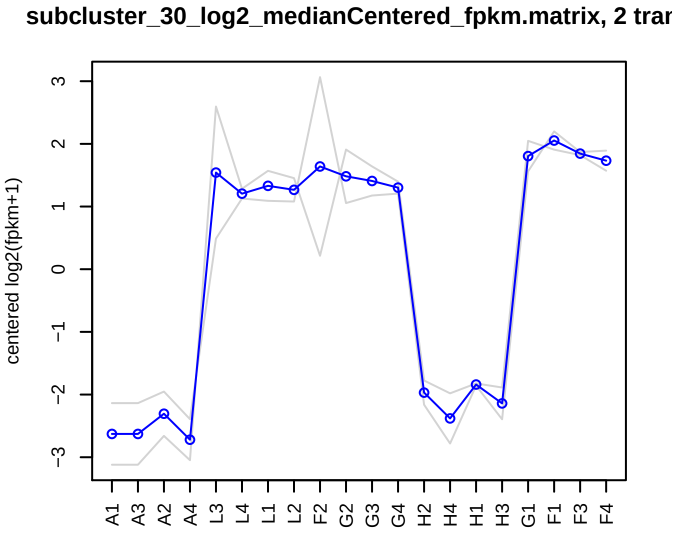
subcluster_30_log2_medianCentered_fpkm.matrix, 2 trar
centered log2(fpkm+1)
3
2
1
0
−1
−2
−3
A1 A3 A2 A4 L3 L4 L1 L2 F2 G2 G3 G4 H2 H4 H1 H3 G1 F1 F3 F4

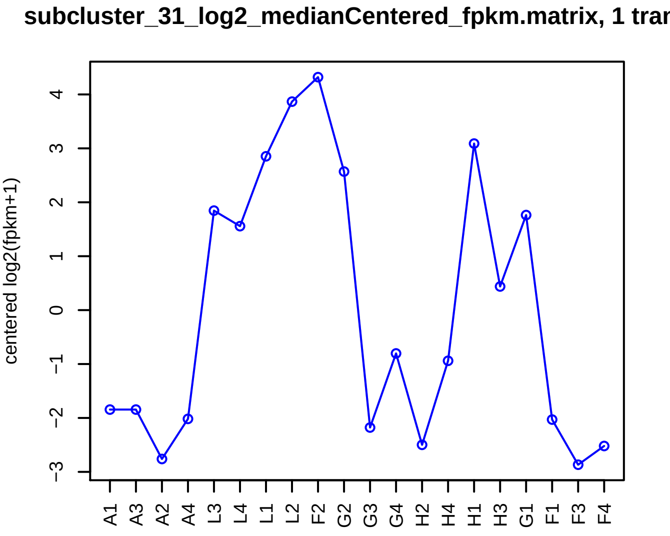
subcluster_31_log2_medianCentered_fpkm.matrix, 1 trar
centered log2(fpkm+1)
4
3
2
1
0
−1
−2
−3
A1 A3 A2 A4 L3 L4 L1 L2 F2 G2 G3 G4 H2 H4 H1 H3 G1 F1 F3 F4

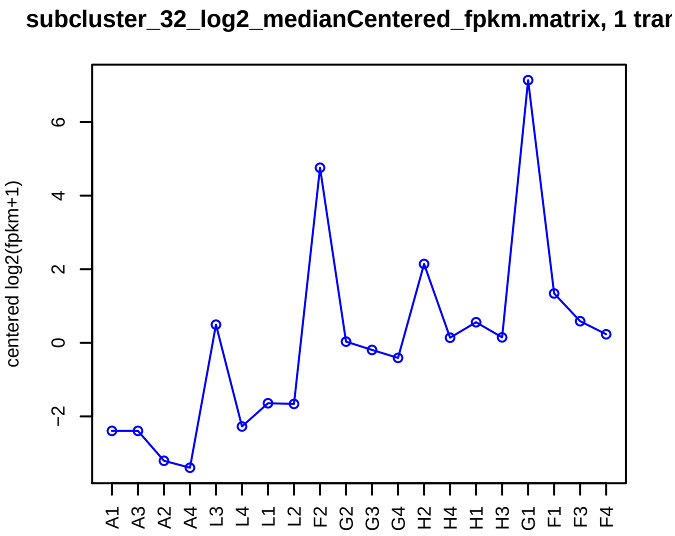
subcluster_32_log2_medianCentered_fpkm.matrix, 1 trar
centered log2(fpkm+1)
−2
0
2
4
6
A1 A3 A2 A4 L3 L4 L1 L2 F2 G2 G3 G4 H2 H4 H1 H3 G1 F1 F3 F4

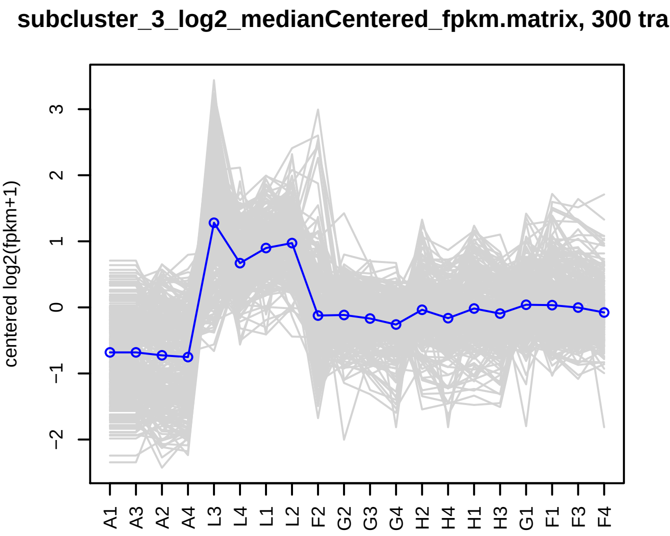
subcluster_3_log2_medianCentered_fpkm.matrix, 300 tra
centered log2(fpkm+1)
−2
−1
0
1
2
3
A1 A3 A2 A4 L3 L4 L1 L2 F2 G2 G3 G4 H2 H4 H1 H3 G1 F1 F3 F4

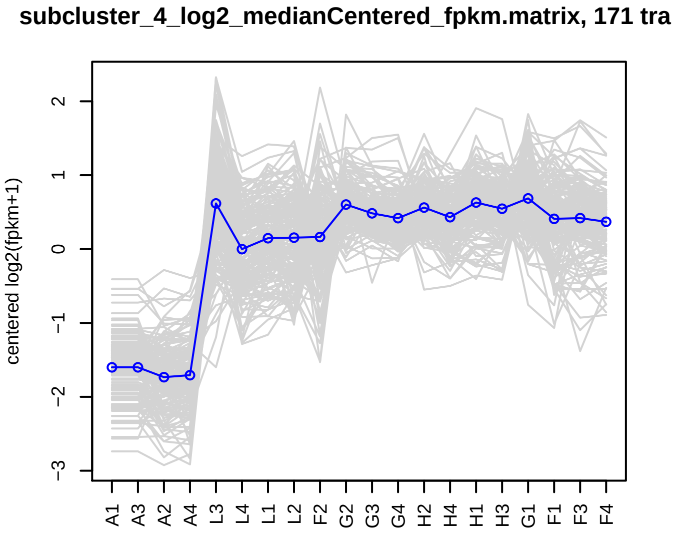
subcluster_4_log2_medianCentered_fpkm.matrix, 171 tra
centered log2(fpkm+1)
−3
−2
−1
0
1
2
A1 A3 A2 A4 L3 L4 L1 L2 F2 G2 G3 G4 H2 H4 H1 H3 G1 F1 F3 F4

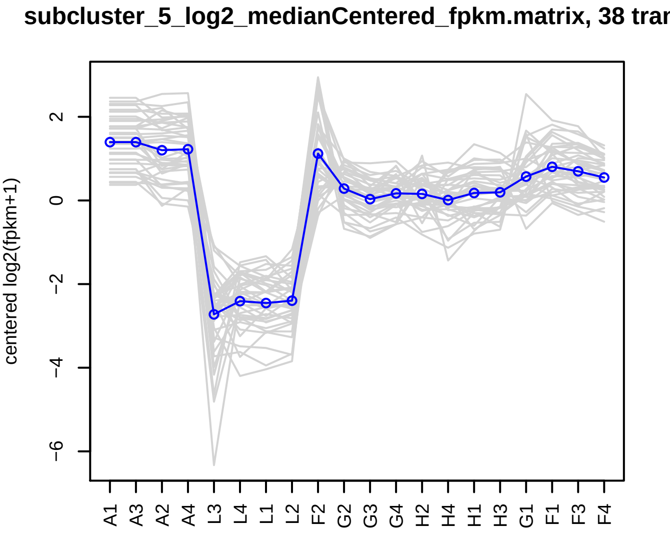
subcluster_5_log2_medianCentered_fpkm.matrix, 38 trar
centered log2(fpkm+1)
−6
−4
−2
0
2
A1 A3 A2 A4 L3 L4 L1 L2 F2 G2 G3 G4 H2 H4 H1 H3 G1 F1 F3 F4

subcluster_6_log2_medianCentered_fpkm.matrix, 308 tra

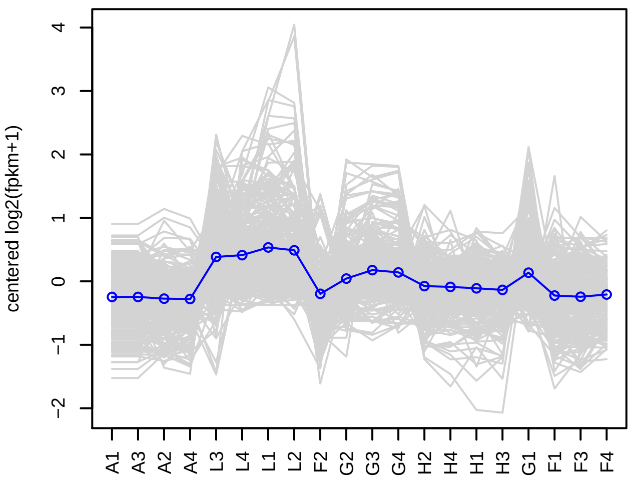

subcluster_7_log2_medianCentered_fpkm.matrix, 50 trar

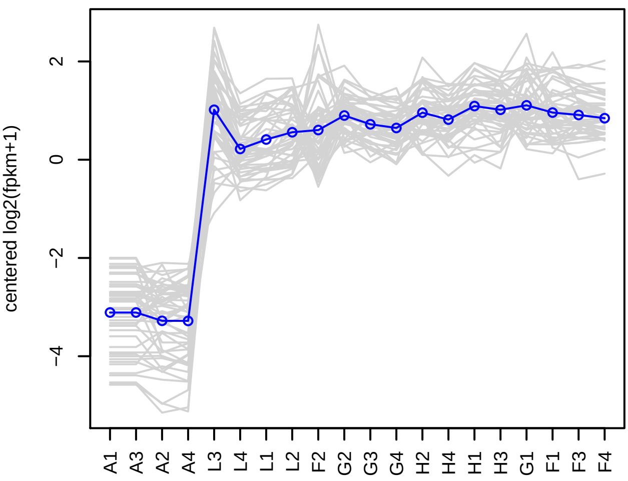

subcluster_8_log2_medianCentered_fpkm.matrix, 81 trar

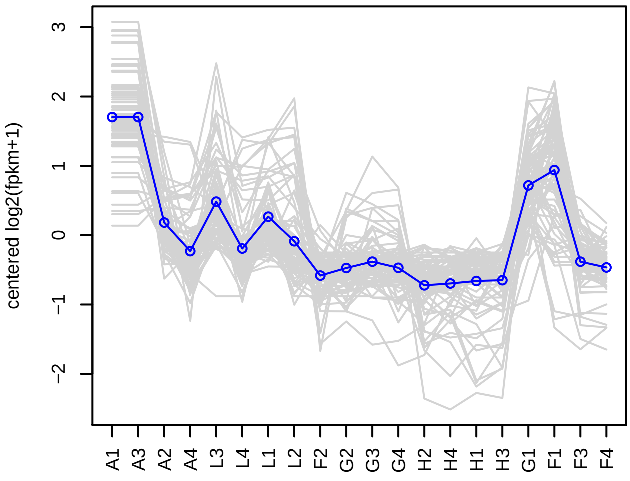

subcluster_9_log2_medianCentered_fpkm.matrix, 267 tra

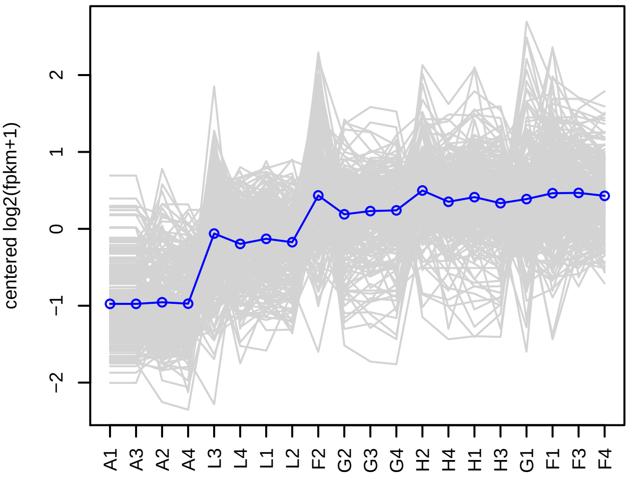

Supplement: Additional file 3: Figure S1. — Clusters of assembled transcripts of R. stricta in apical leaves (A1-L4) at different time points of the day (A, morning; F-H, midday & L, dusk). Grey lines indicate expression patterns of individual transcripts in a given cluster. Blue lines indicate overall expression pattern across different transcripts of a given cluster. (PDF 223 kb) [file 12870_2016_938_MOESM3_ESM.pdf]
