# Supplementary material for: Analysis of transcriptional response to heat stress in Rhazya stricta
Source: BMC Plant Biol. 2016 Nov 14;16:252. doi: 10.1186/s12870-016-0938-6 (PMC5109689; doi:10.1186/s12870-016-0938-6)

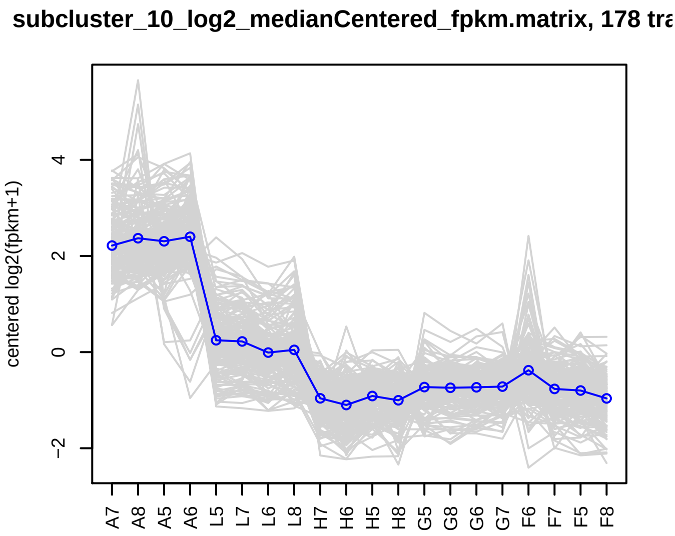
subcluster_10_log2_medianCentered_fpkm.matrix, 178 tra
centered log2(fpkm+1)
−2
0
2
4
A7 A8 A5 A6 L5 L7 L6 L8 H7 H6 H5 H8 G5 G8 G6 G7 F6 F7 F5 F8

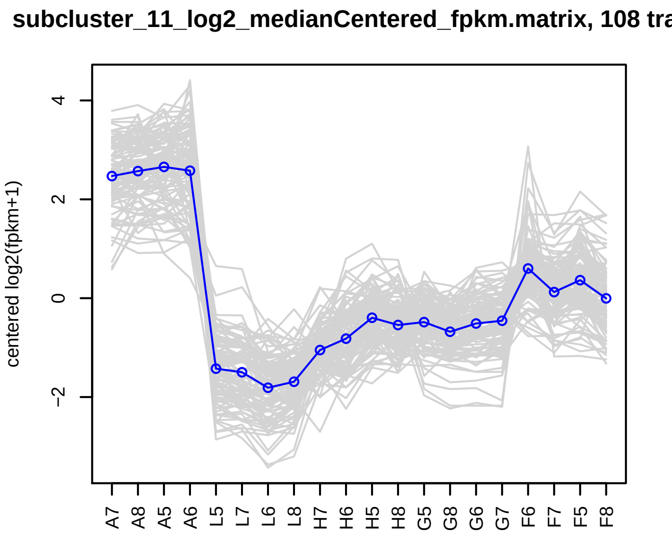
subcluster_11_log2_medianCentered_fpkm.matrix, 108 tra
centered log2(fpkm+1)
−2
0
2
4
A7 A8 A5 A6 L5 L7 L6 L8 H7 H6 H5 H8 G5 G8 G6 G7 F6 F7 F5 F8

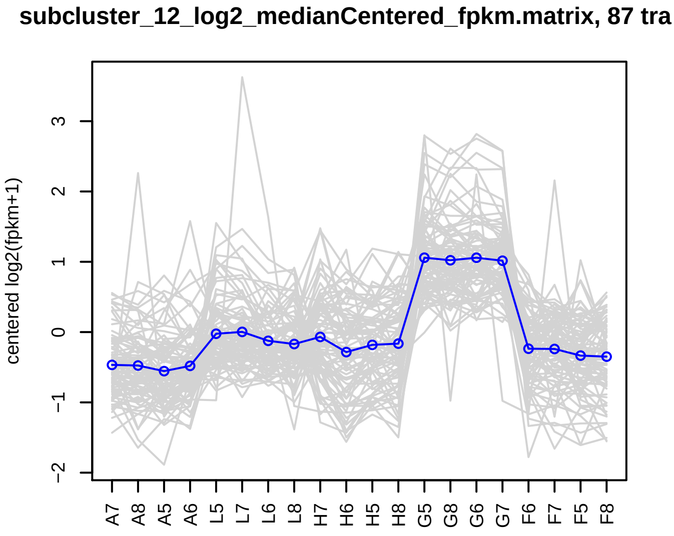
subcluster_12_log2_medianCentered_fpkm.matrix, 87 tra
centered log2(fpkm+1)
−2
−1
0
1
2
3
A7 A8 A5 A6 L5 L7 L6 L8 H7 H6 H5 H8 G5 G8 G6 G7 F6 F7 F5 F8

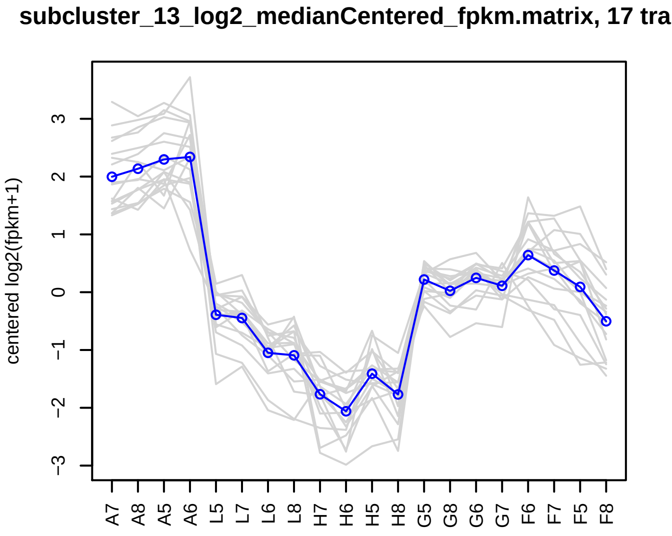
subcluster_13_log2_medianCentered_fpkm.matrix, 17 tra
centered log2(fpkm+1)
−3
−2
−1
0
1
2
3
A7 A8 A5 A6 L5 L7 L6 L8 H7 H6 H5 H8 G5 G8 G6 G7 F6 F7 F5 F8

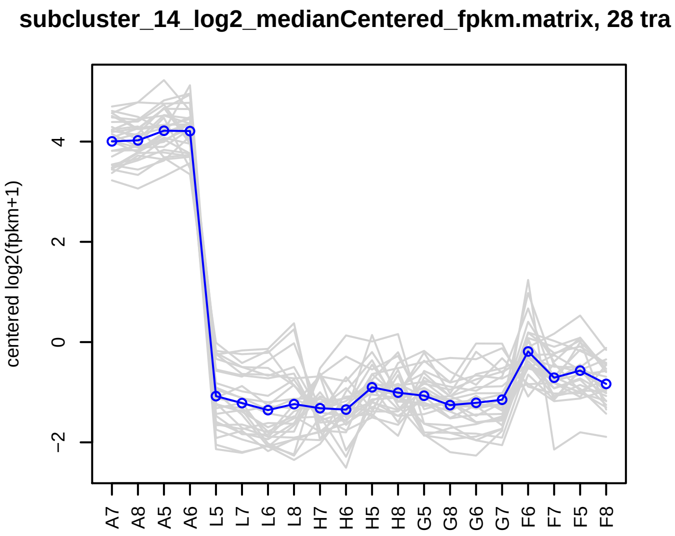
subcluster_14_log2_medianCentered_fpkm.matrix, 28 tra
centered log2(fpkm+1)
A7 A8 A5 A6 L5 L7 L6 L8 H7 H6 H5 H8 G5 G8 G6 G7 F6 F7 F5 F8

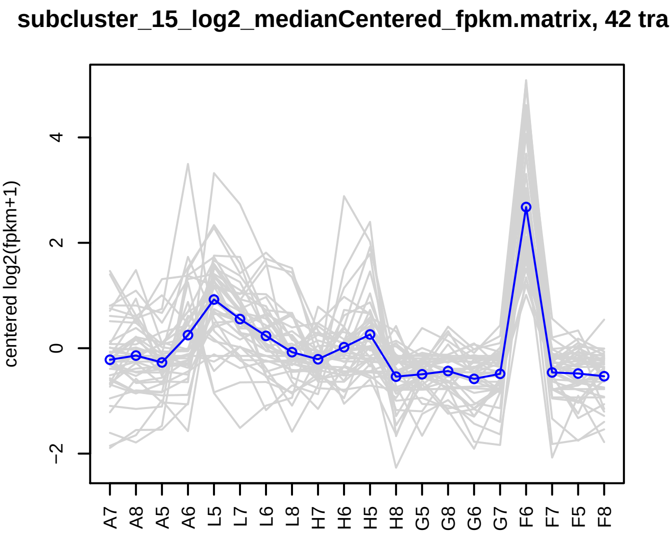
subcluster_15_log2_medianCentered_fpkm.matrix, 42 tra
centered log2(fpkm+1)
A7 A8 A5 A6 L5 L7 L6 L8 H7 H6 H5 H8 G5 G8 G6 G7 F6 F7 F5 F8

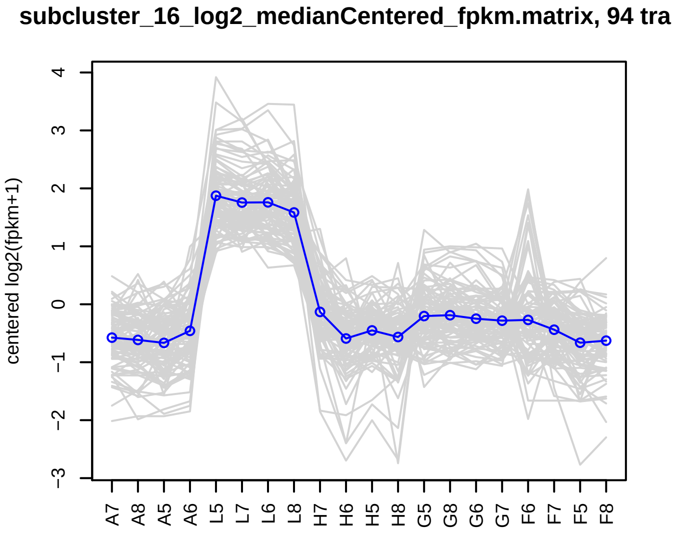
subcluster_16_log2_medianCentered_fpkm.matrix, 94 tra
centered log2(fpkm+1)
A7 A8 A5 A6 L5 L7 L6 L8 H7 H6 H5 H8 G5 G8 G6 G7 F6 F7 F5 F8

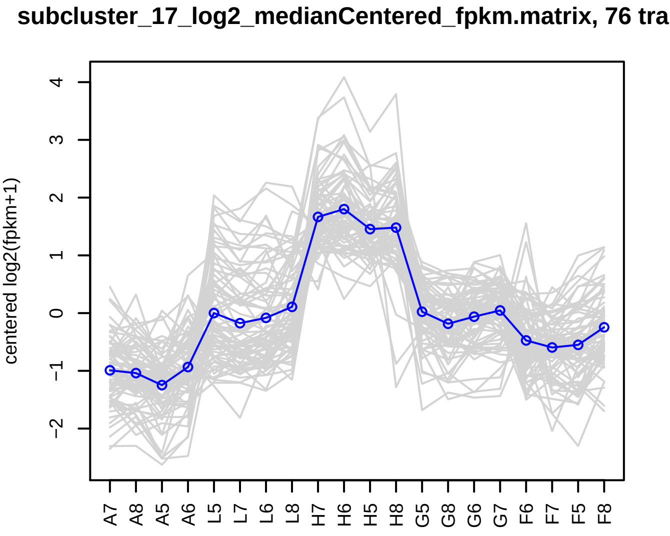
subcluster_17_log2_medianCentered_fpkm.matrix, 76 tra
centered log2(fpkm+1)
A7 A8 A5 A6 L5 L7 L6 L8 H7 H6 H5 H8 G5 G8 G6 G7 F6 F7 F5 F8

subcluster_18_log2_medianCentered_fpkm.matrix, 91 tra

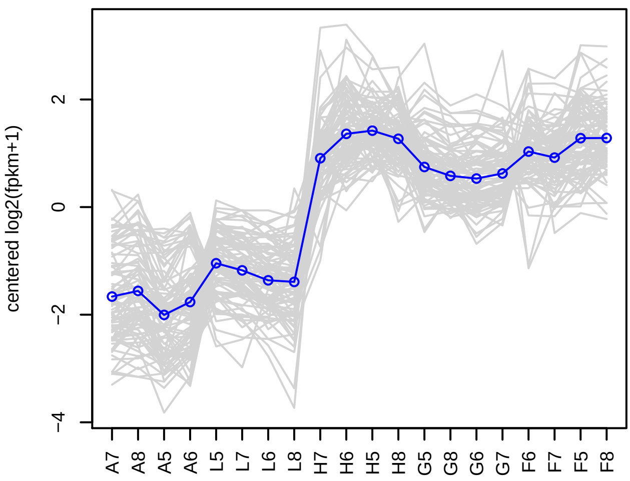

subcluster_19_log2_medianCentered_fpkm.matrix, 14 tra

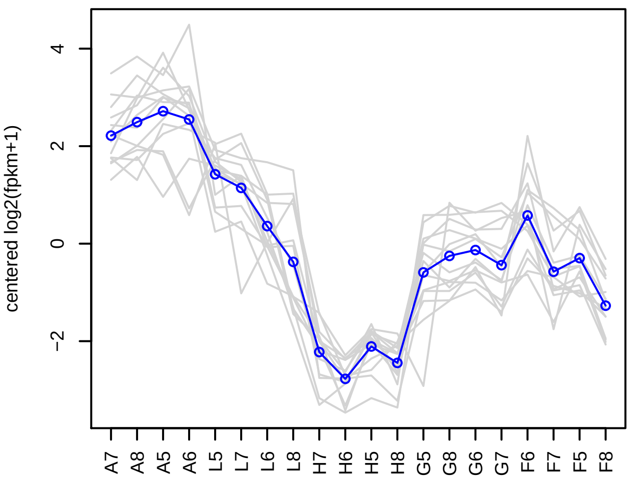

subcluster_1_log2_medianCentered_fpkm.matrix, 27 trar

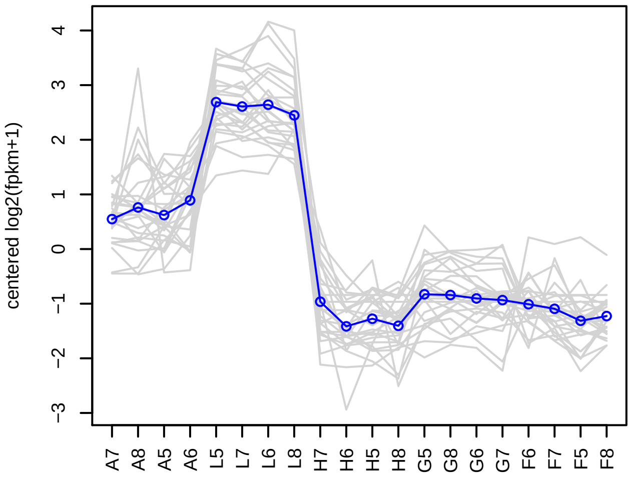

subcluster_20_log2_medianCentered_fpkm.matrix, 33 tra

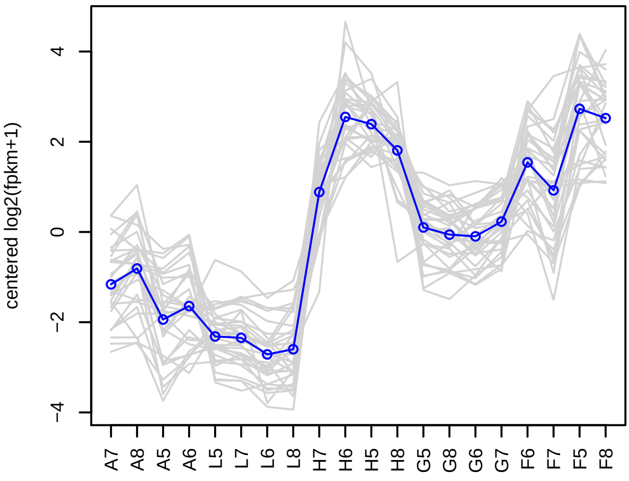

subcluster_21_log2_medianCentered_fpkm.matrix, 7 trar

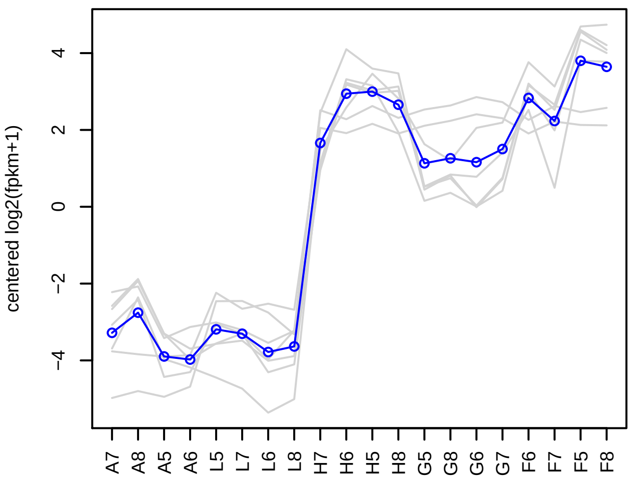

subcluster_22_log2_medianCentered_fpkm.matrix, 18 tra

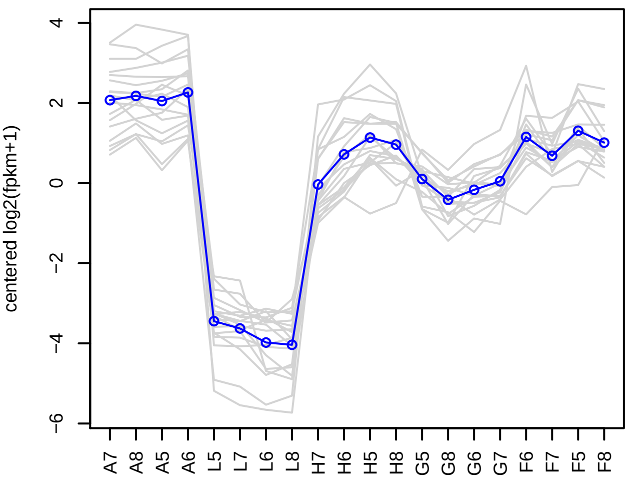

subcluster_23_log2_medianCentered_fpkm.matrix, 2 trar

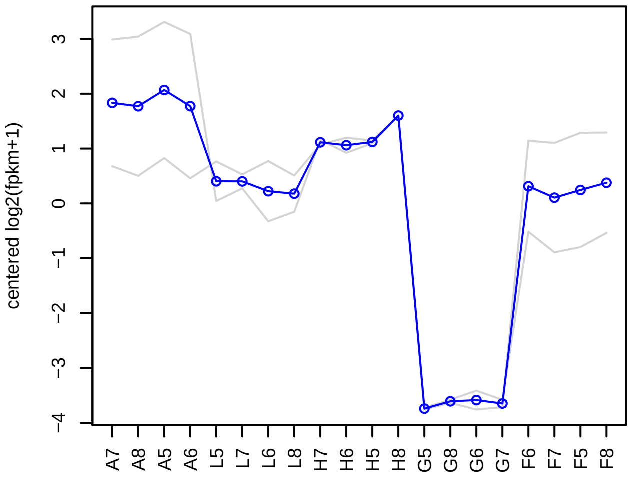

subcluster_24_log2_medianCentered_fpkm.matrix, 6 trar

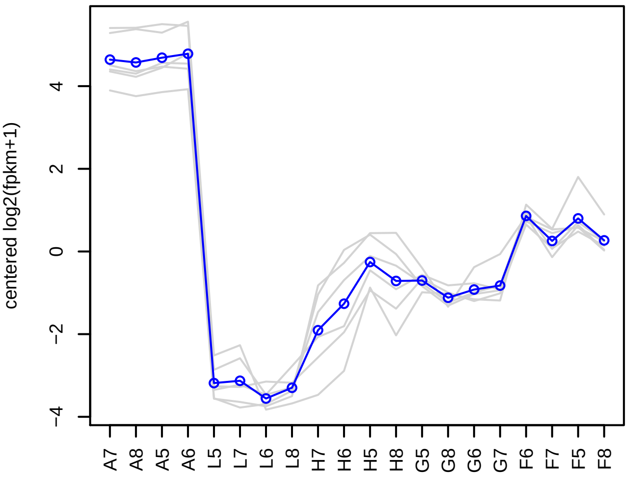

subcluster_25_log2_medianCentered_fpkm.matrix, 19 tra

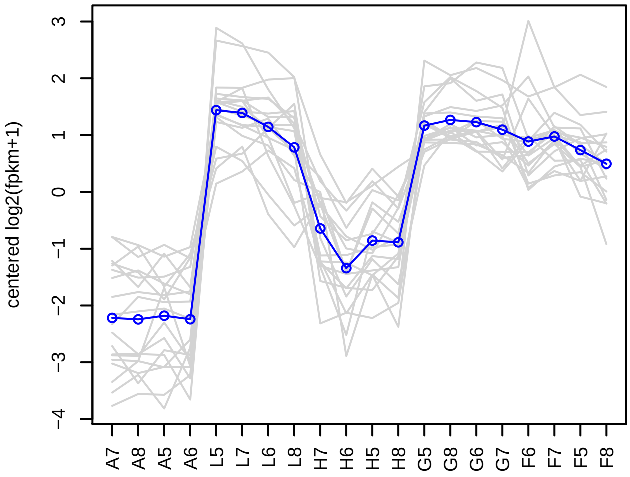

subcluster_26_log2_medianCentered_fpkm.matrix, 47 tra

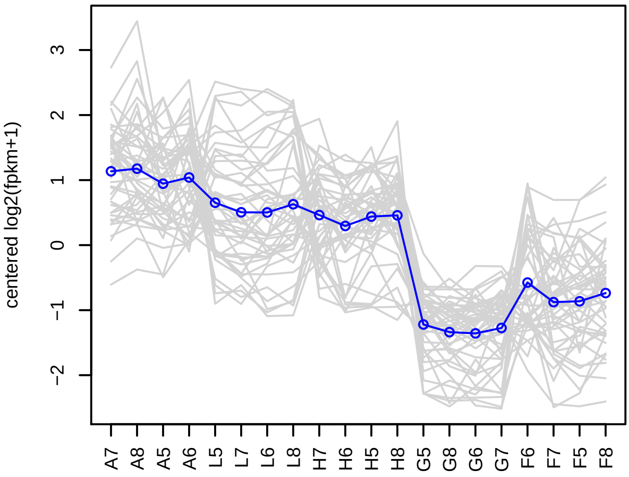

subcluster_27_log2_medianCentered_fpkm.matrix, 30 tra

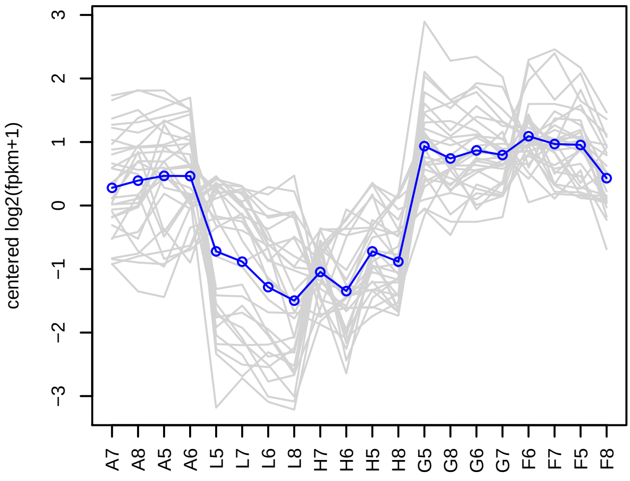

subcluster_28_log2_medianCentered_fpkm.matrix, 13 tra

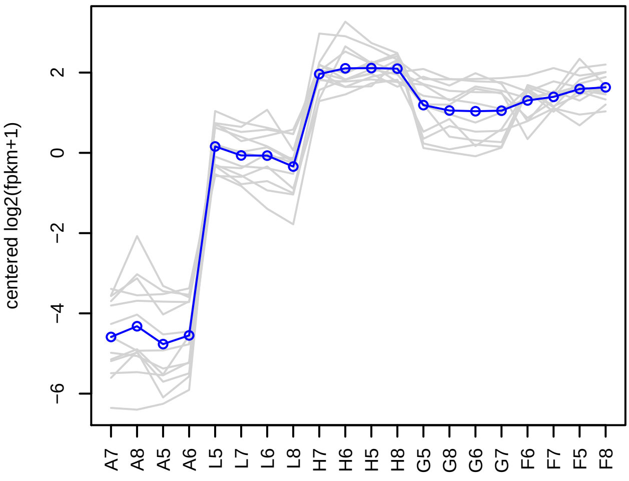

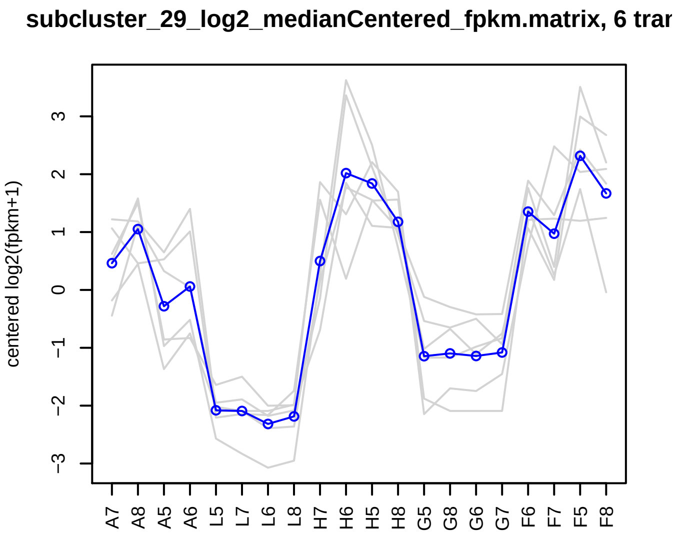
subcluster_29_log2_medianCentered_fpkm.matrix, 6 trar
centered log2(fpkm+1)
−3 −2 −1 0 1 2 3
A7 A8 A5 A6 L5 L7 L6 L8 H7 H6 H5 H8 G5 G8 G6 G7 F6 F7 F5 F8

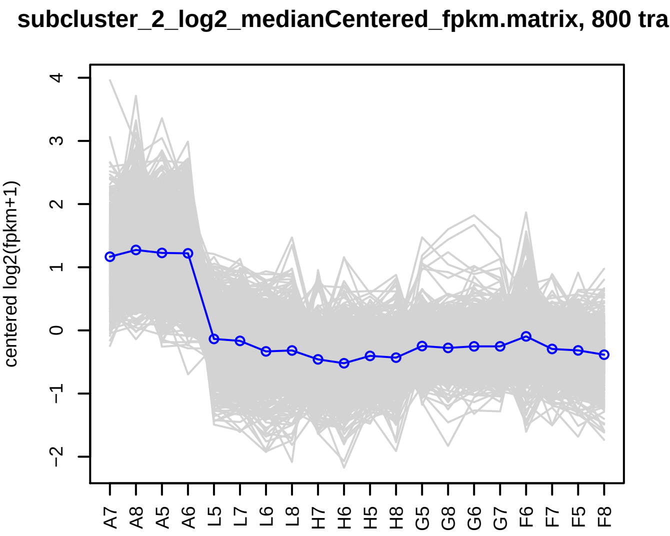
subcluster_2_log2_medianCentered_fpkm.matrix, 800 tra
centered log2(fpkm+1)
−2 −1 0 1 2 3 4
A7 A8 A5 A6 L5 L7 L6 L8 H7 H6 H5 H8 G5 G8 G6 G7 F6 F7 F5 F8

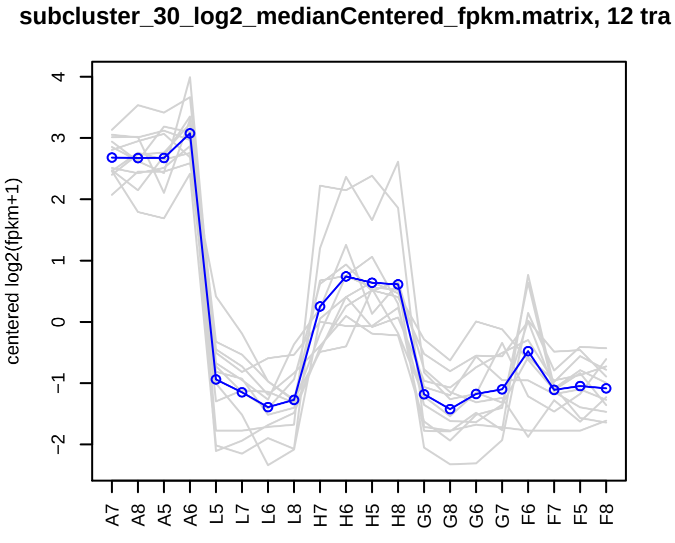
subcluster_30_log2_medianCentered_fpkm.matrix, 12 tra
centered log2(fpkm+1)
−2 −1 0 1 2 3 4
A7 A8 A5 A6 L5 L7 L6 L8 H7 H6 H5 H8 G5 G8 G6 G7 F6 F7 F5 F8

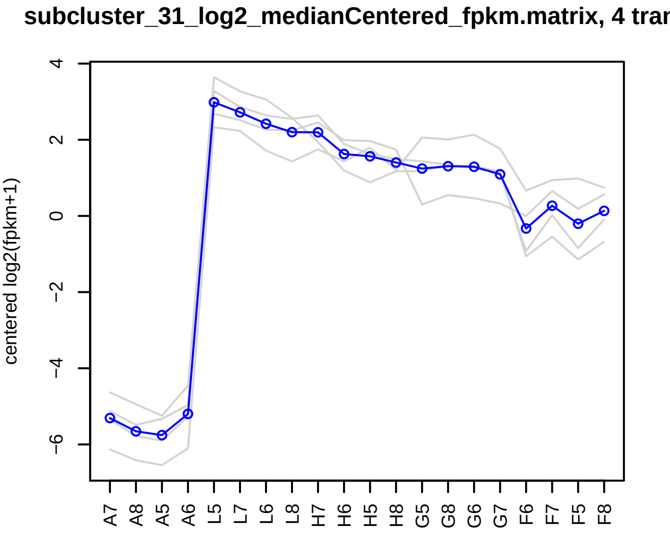
subcluster_31_log2_medianCentered_fpkm.matrix, 4 trar
centered log2(fpkm+1)
−6 −4 −2 0 2 4
A7 A8 A5 A6 L5 L7 L6 L8 H7 H6 H5 H8 G5 G8 G6 G7 F6 F7 F5 F8

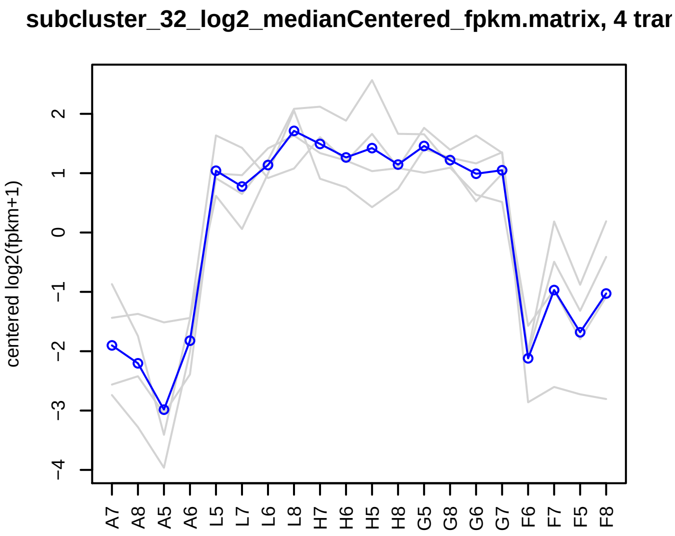
subcluster_32_log2_medianCentered_fpkm.matrix, 4 trar
centered log2(fpkm+1)
A7 A8 A5 A6 L5 L7 L6 L8 H7 H6 H5 H8 G5 G8 G6 G7 F6 F7 F5 F8

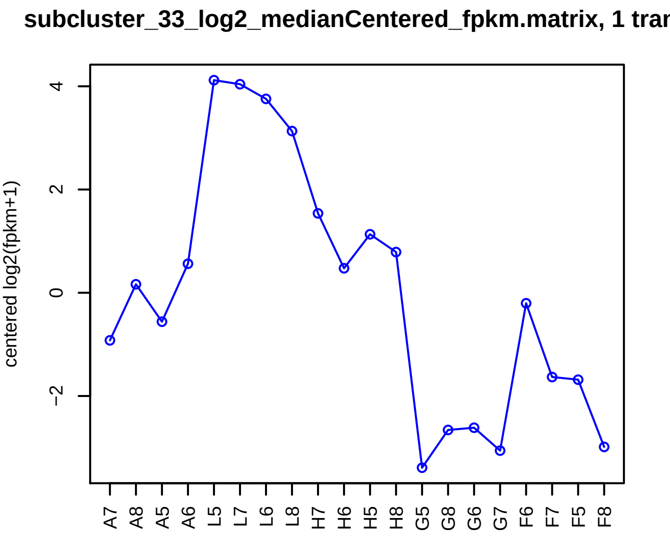
subcluster_33_log2_medianCentered_fpkm.matrix, 1 trar
centered log2(fpkm+1)
A7 A8 A5 A6 L5 L7 L6 L8 H7 H6 H5 H8 G5 G8 G6 G7 F6 F7 F5 F8

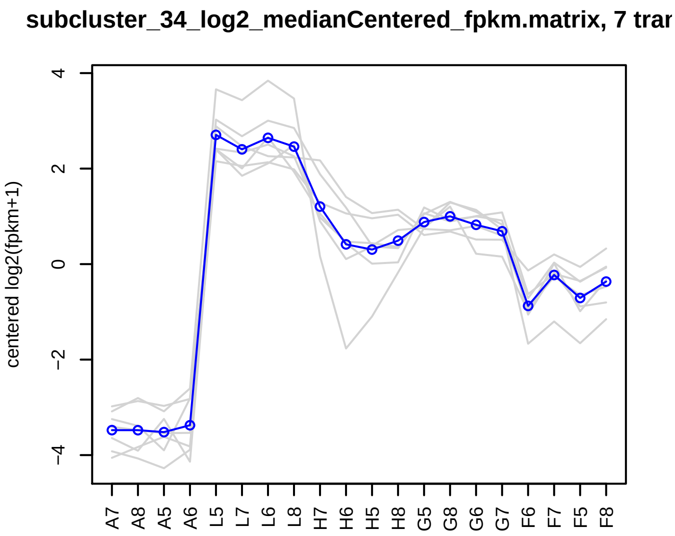
subcluster_34_log2_medianCentered_fpkm.matrix, 7 trar
centered log2(fpkm+1)
A7 A8 A5 A6 L5 L7 L6 L8 H7 H6 H5 H8 G5 G8 G6 G7 F6 F7 F5 F8

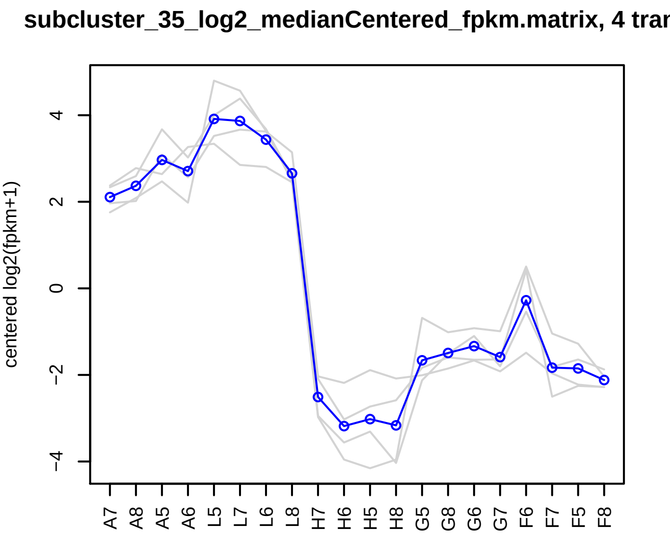
subcluster_35_log2_medianCentered_fpkm.matrix, 4 trar
centered log2(fpkm+1)
A7 A8 A5 A6 L5 L7 L6 L8 H7 H6 H5 H8 G5 G8 G6 G7 F6 F7 F5 F8

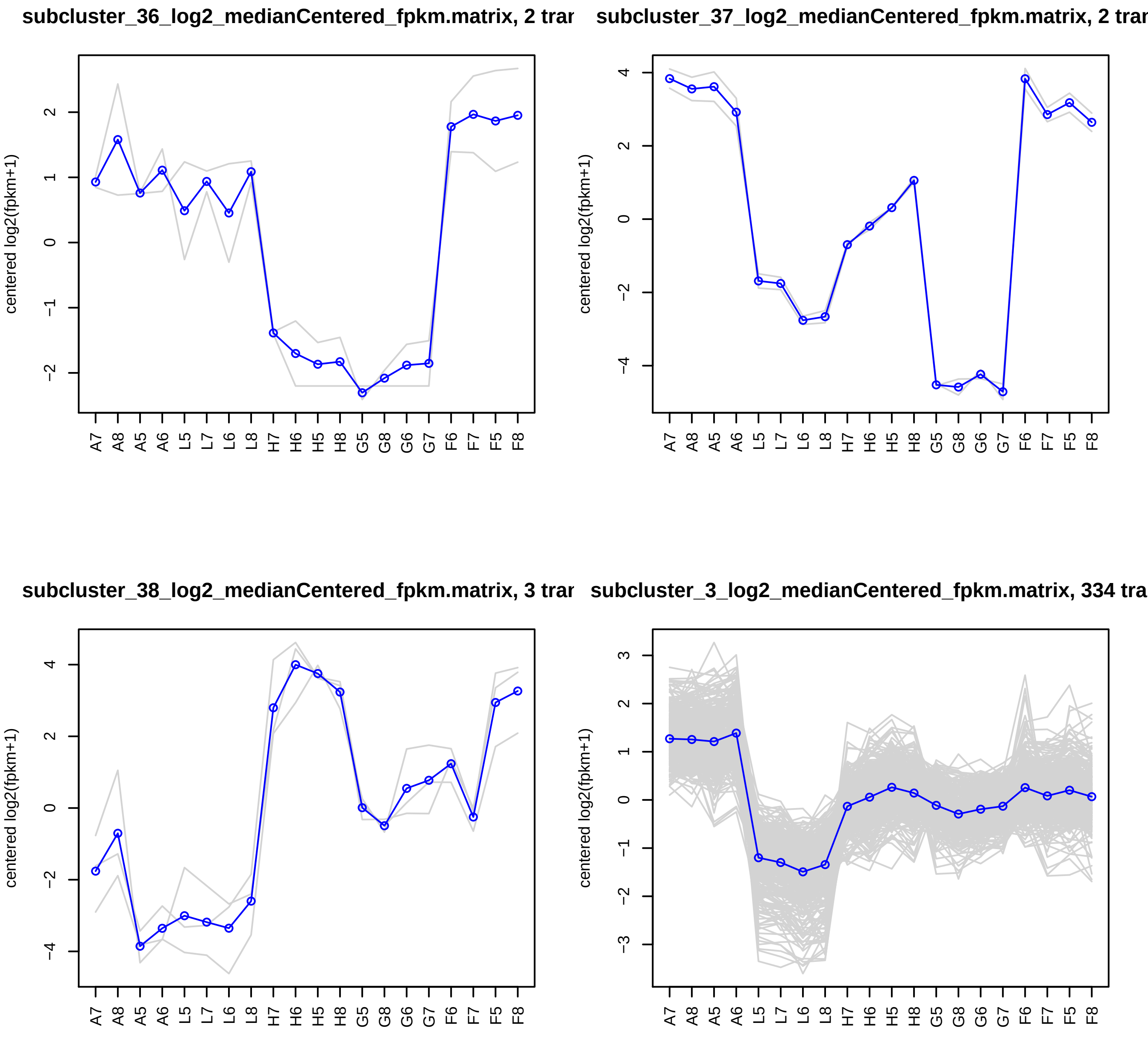

subcluster_36_log2_medianCentered_fpkm.matrix, 2 trar
subcluster_37_log2_medianCentered_fpkm.matrix, 2 trar
subcluster_38_log2_medianCentered_fpkm.matrix, 3 trar
subcluster_3_log2_medianCentered_fpkm.matrix, 334 tra
centered log2(fpkm+1)
A7 A8 A5 A6 L5 L7 L6 L8 H7 H6 H5 H8 G5 G8 G6 G7 F6 F7 F5 F8

subcluster_4_log2_medianCentered_fpkm.matrix, 886 tra

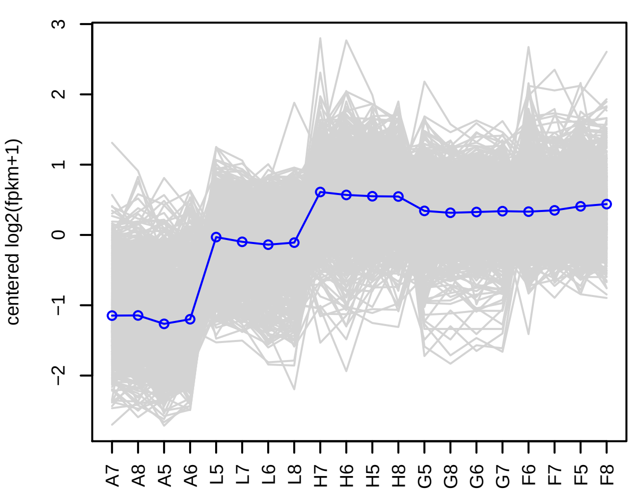

subcluster_5_log2_medianCentered_fpkm.matrix, 878 tra

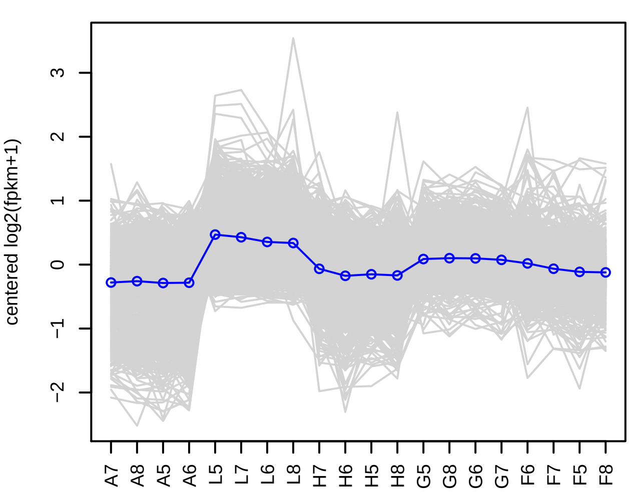

subcluster_6_log2_medianCentered_fpkm.matrix, 495 tra

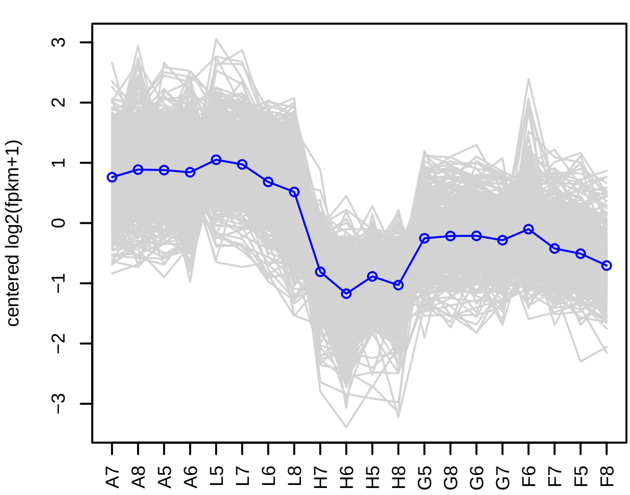

subcluster_7_log2_medianCentered_fpkm.matrix, 175 tra

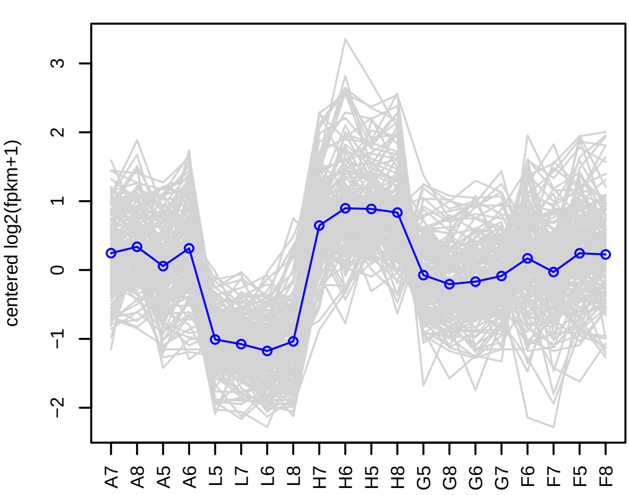

subcluster_8_log2_medianCentered_fpkm.matrix, 288 tra

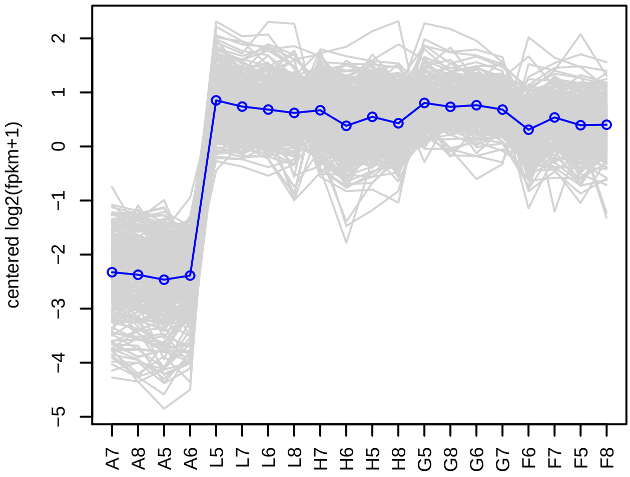

subcluster_9_log2_medianCentered_fpkm.matrix, 15 tran

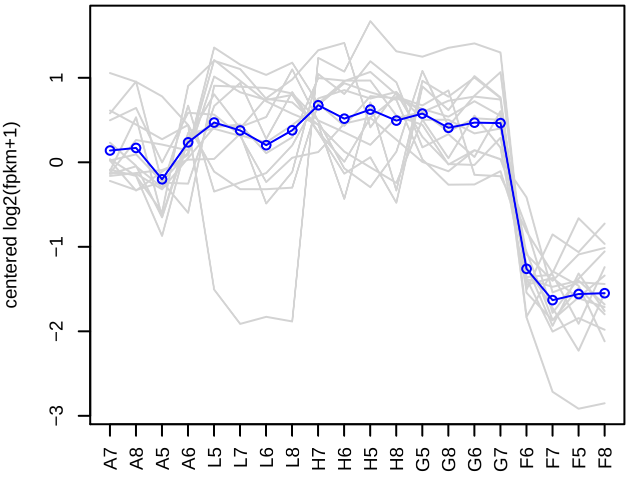

Supplement: Additional file 4: Figure S2. — Clusters of assembled transcripts of R. stricta SRA in mature leaves (A5-L8) at different time points of the day (A, morning; F-H, midday & L, dusk). Grey lines indicate expression patterns of individual transcripts in a given cluster. Blue lines indicate overall expression pattern across different transcripts of a given cluster. (PDF 397 kb) [file 12870_2016_938_MOESM4_ESM.pdf]
