# Supplementary material for: Analysis of transcriptional response to heat stress in Rhazya stricta
Source: BMC Plant Biol. 2016 Nov 14;16:252. doi: 10.1186/s12870-016-0938-6 (PMC5109689; doi:10.1186/s12870-016-0938-6)
Supplement: Additional file 5: Figure S3. — Semi-quantitative RT-PCR and profiles of fold change values resulting from RNA-Seq analysis for selected upregulated genes starting midday (1–5) and gradually downregulated genes (6-10) used for validating RNA-Seq data of apical (A1-L4) and mature (A5-L7) leaves of R. stricta collected at different time points of the day. Serial numbers 1–10 refer to genes described in Additional file 6: Table S3. The “actin” gene was used as the unregulated house-keeping gene. (DOCX 9867 kb) [file 12870_2016_938_MOESM5_ESM.docx]

a. *actin* gene


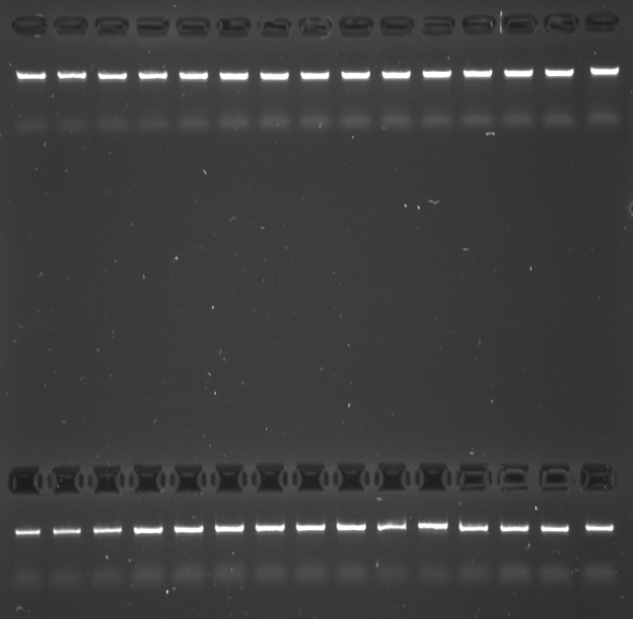

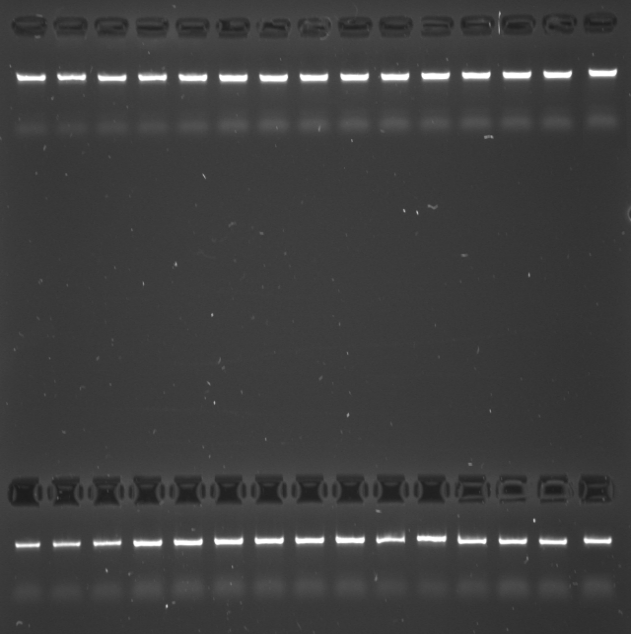

b. Selected upregulated genes starting midday

c. Selected gradually downregulated genes

Figure S3.
