# Supplementary figures and images for: Analysis of transcriptional response to heat stress in Rhazya stricta
Source: BMC Plant Biol. 2016 Nov 14;16:252. doi: 10.1186/s12870-016-0938-6 (PMC5109689; doi:10.1186/s12870-016-0938-6)

Figure S4.


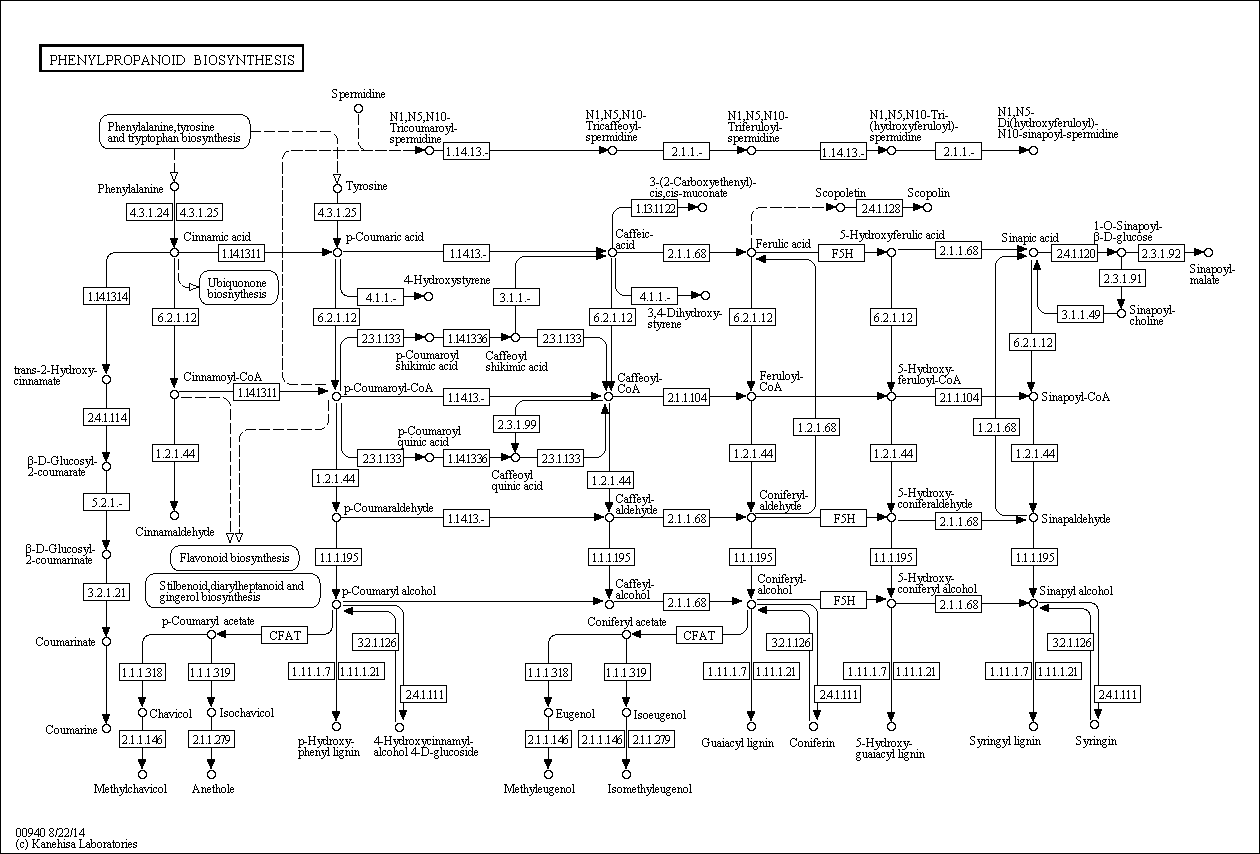

Supplement: Additional file 7: Figure S4. — Enzymes in the phenylpropanoid metabolic pathway in apical and mature leaves responded differentially to changing environment at two time points (morning, A and midday, G). Upregulated (activated) in apical leaves (blue), upregulated in mature leaves (red), downregulated (repressed) in apical leaves (orange box), downregulated in mature leaves (green box). (DOCX 174 kb) [file 12870_2016_938_MOESM7_ESM.docx]

Figure S5.


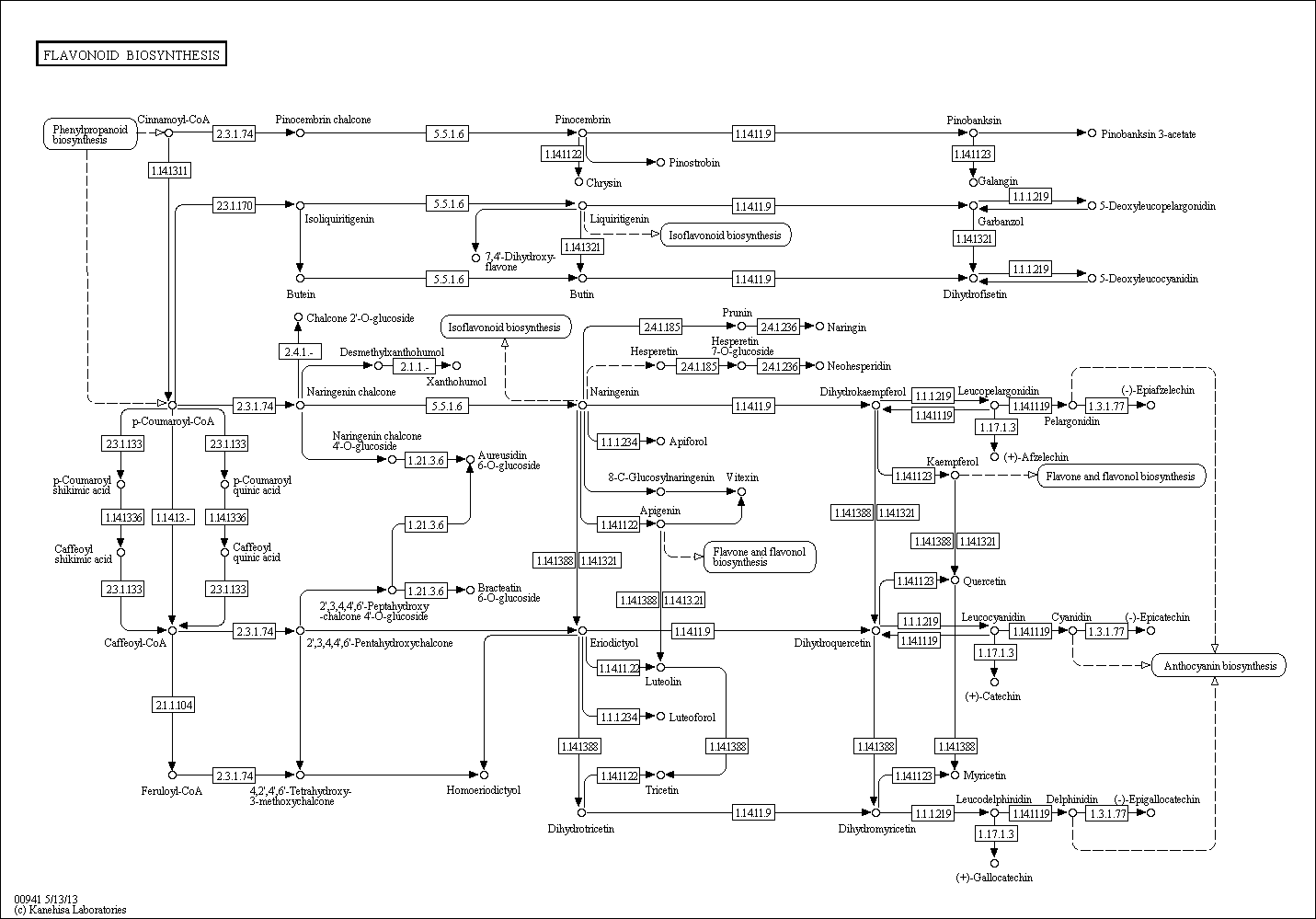

Supplement: Additional file 8: Figure S5. — Enzymes in the flavonoid metabolic pathway in apical and mature leaves responded differentially to changing environments at two time points (morning, A and midday, G). Upregulated (activated) in apical leaves (blue), upregulated in mature leaves (red). (DOCX 193 kb) [file 12870_2016_938_MOESM8_ESM.docx]

Figure S6.


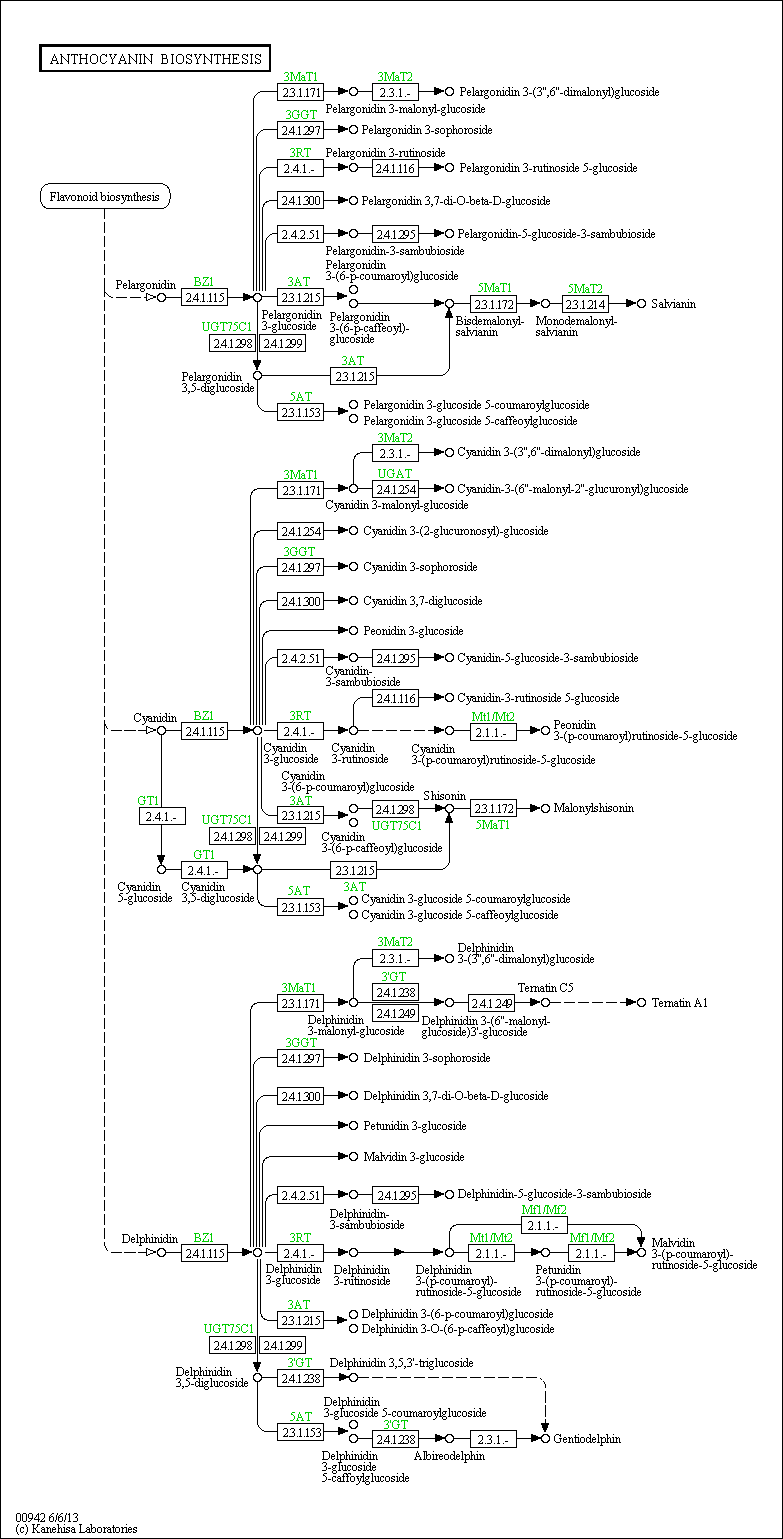

Supplement: Additional file 9: Figure S6. — Enzymes in the anthocyanin metabolic pathway in apical and mature leaves responded differentially to changing environment at two time points (morning, A and midday, G). Upregulated (activated) in apical leaves (blue), upregulated in mature leaves (red). (DOCX 210 kb) [file 12870_2016_938_MOESM9_ESM.docx]

Figure S7.


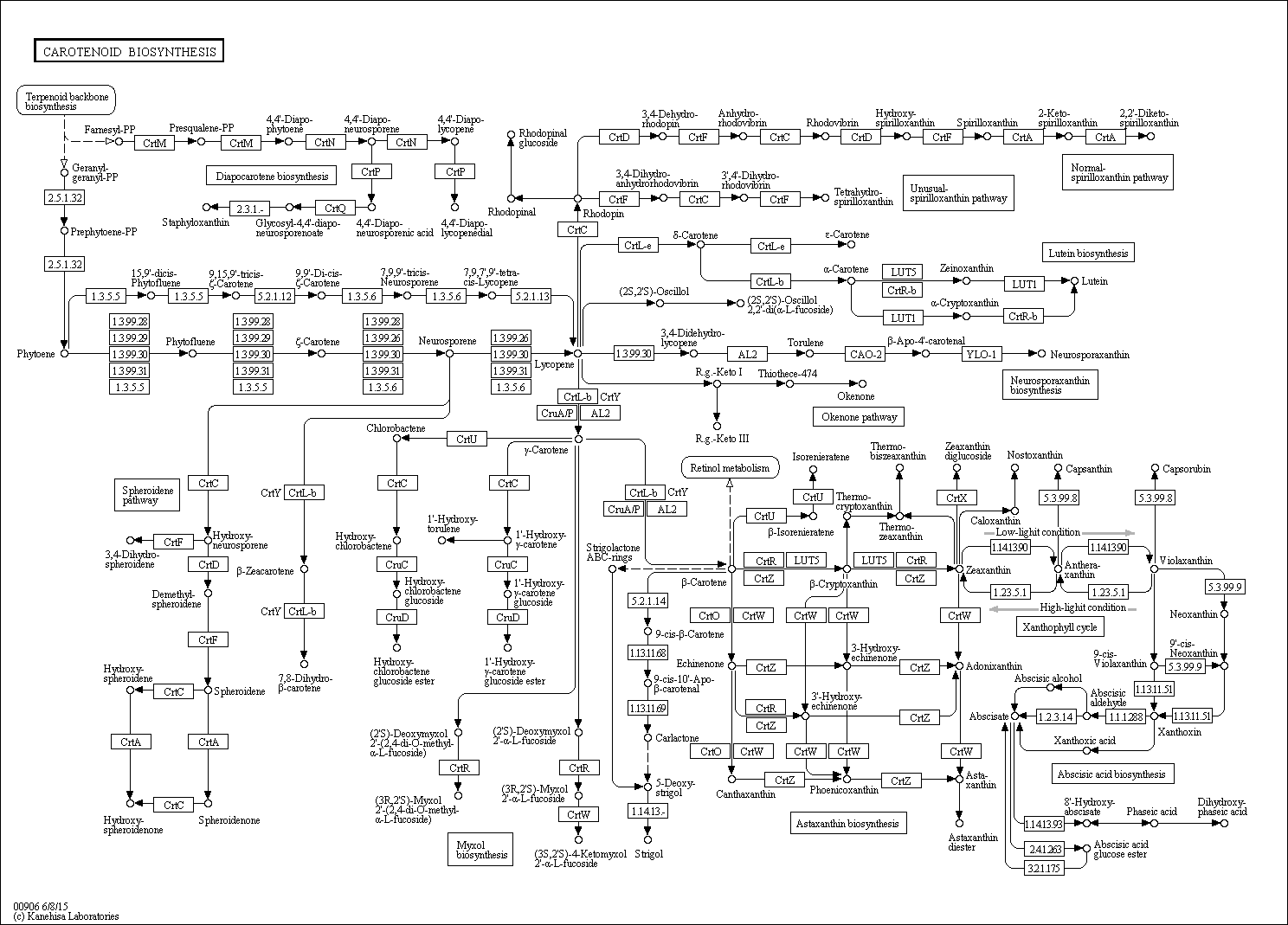

Supplement: Additional file 10: Figure S7. — Enzymes in the carotenoid metabolic pathway in apical and mature leaves responded differentially to changing environment at two time points (morning, A and midday, G). Upregulated (activated) in apical leaves (blue), upregulated in mature leaves (red), downregulated (repressed) in apical leaves (orange box), downregulated in mature leaves (green box). (DOCX 242 kb) [file 12870_2016_938_MOESM10_ESM.docx]

Figure S8.


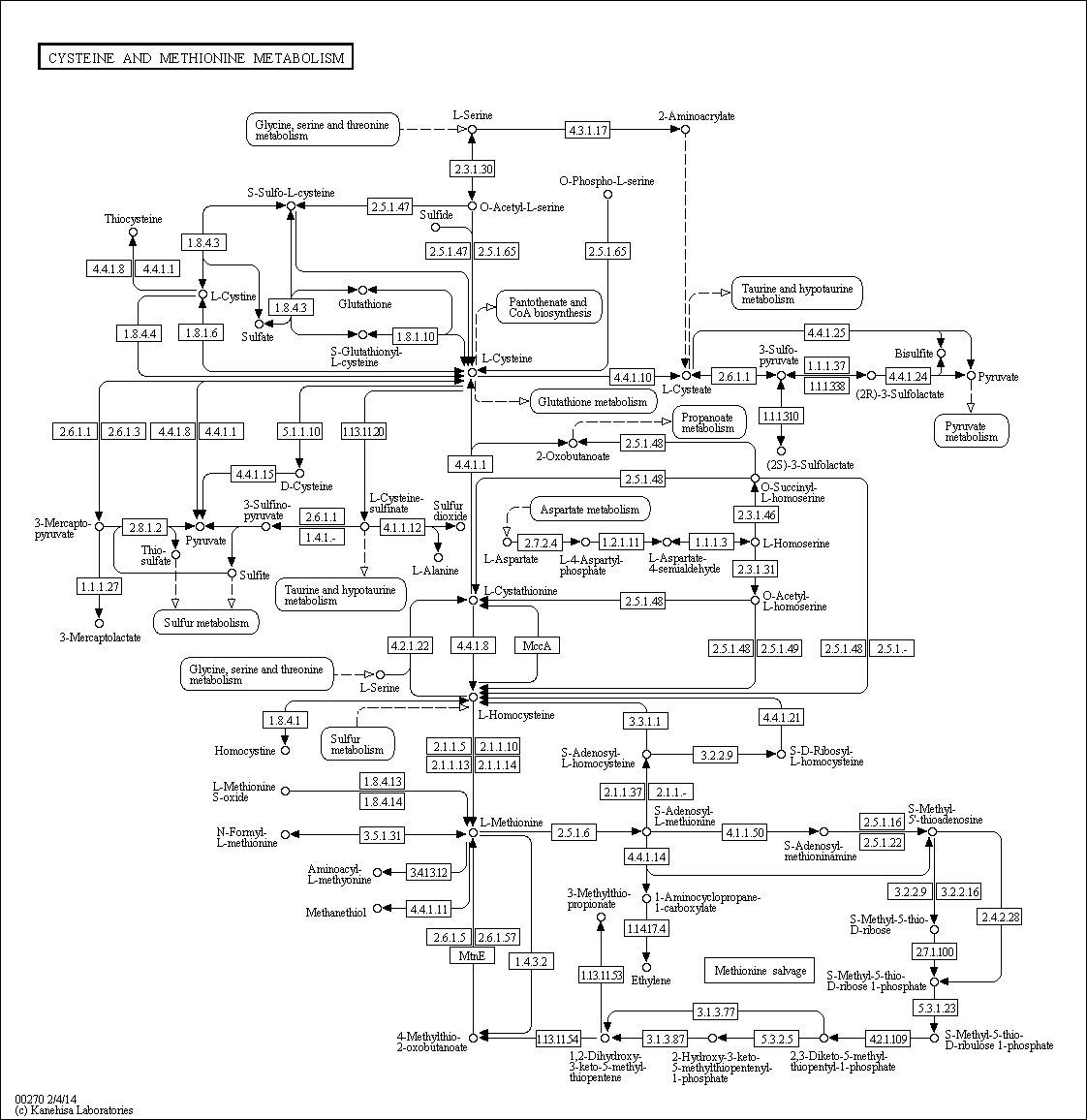

Supplement: Additional file 11: Figure S8. — Enzymes in the cysteine and methionine metabolic pathways in apical and mature leaves responded differentially to changing environment at two time points (morning, A and midday, G). Upregulated (activated) in apical leaves (blue), upregulated in mature leaves (red), downregulated (repressed) in apical leaves (orange box), downregulated in mature leaves (green box). (DOCX 201 kb) [file 12870_2016_938_MOESM11_ESM.docx]
