# Supplementary material for: Analysis of transcriptional response to heat stress in Rhazya stricta
Source: BMC Plant Biol. 2016 Nov 14;16:252. doi: 10.1186/s12870-016-0938-6 (PMC5109689; doi:10.1186/s12870-016-0938-6)
Supplement: Additional file 12: Table S4. — Comparative differential expression of genes of R. stricta transcriptomes of apical (a) and mature (b) leaves with fold change of ≥ 5 in different time points (A, morning; F-H, midday & L, dusk). Blue box = upregulation, orange box = downregulation. Numbers between parentheses represent no. analogs of a given gene. Green boxes indicate genes selected for further analysis. (DOCX 80 kb) [file 12870_2016_938_MOESM12_ESM.docx]

Table S4. Comparative differential expression of genes of *R.* *stricta* transcriptomes of apical (a) and mature (b) leaves with fold change of ≥ 5 in different time points (A, morning; F-H, midday & L, dusk). Blue box = upregulation, orange box = downregulation. Numbers between parentheses represent no. analogs of a given gene. Green boxes indicate genes selected for further analysis.

| (a) Apical leaves | | | | | | | | | | | |  |
| --- | --- | --- | --- | --- | --- | --- | --- | --- | --- | --- | --- | --- |
| Gene description | Time point with fold change of ≥ 5 | F | G | H | L | F | | G | F | G | H |  |
|  | Time point in comparison | A | | | | G | H | | L | | |  |
| alpha-crystallin domain isoform 1 | |  |  |  |  |  |  |  |  |  |  |  |
| secologanin synthase-like (cyp71A1) | |  |  |  |  |  |  |  |  |  |  |  |
| lrr receptor-like serine threonine-protein kinase at3g47570-like | |  |  |  |  |  |  |  |  |  |  |  |
| homeodomain-like superfamily isoform 1 | |  |  |  |  |  |  |  |  |  |  |  |
| wall-associated receptor kinase-like 1-like | |  |  |  |  |  |  |  |  |  |  |  |
| lrr receptor-like serine threonine-protein kinase at3g47570-like | |  |  |  |  |  |  |  |  |  |  |  |
| late embryogenesis abundant protein d-34-like | |  |  |  |  |  |  |  |  |  |  |  |
| xyloglucan endotransglucosylase hydrolase protein 23-like | |  |  |  |  |  |  |  |  |  |  |  |
| peptide transporter ptr1 | |  |  |  |  |  |  |  |  |  |  |  |
| ammonium transporter 3 member 1-like | |  |  |  |  |  |  |  |  |  |  |  |
| zinc finger family protein | |  |  |  |  |  |  |  |  |  |  |  |
| cytochrome p450 71A1-like | |  |  |  |  |  |  |  |  |  |  |  |
| cytochrome p450 | |  |  |  |  |  |  |  |  |  |  |  |
| dicer-like protein isoform 3 | |  |  |  |  |  |  |  |  |  |  |  |
| early nodulin 70 family protein | |  |  |  |  |  |  |  |  |  |  |  |
| ethylene-responsive transcription factor abr1-like | |  |  |  |  |  |  |  |  |  |  |  |
| monothiol glutaredoxin-s1-like | |  |  |  |  |  |  |  |  |  |  |  |
| xyloglucan endotransglucosylase hydrolase protein 33-like | |  |  |  |  |  |  |  |  |  |  |  |
| u-box domain-containing protein 26-like | |  |  |  |  |  |  |  |  |  |  |  |
| protein crabs claw-like | |  |  |  |  |  |  |  |  |  |  |  |
| protein transparent testa 12-like isoform 1 | |  |  |  |  |  |  |  |  |  |  |  |
| 90 KDa class I heat shock protein | |  |  |  |  |  |  |  |  |  |  |  |
| heat shock transcription factor a-1 isoform 1 | |  |  |  |  |  |  |  |  |  |  | |
| zinc finger ccch domain-containing protein 48-like | |  |  |  |  |  |  |  |  |  |  | |
| lrr receptor-like serine threonine-protein kinase at3g47570-like | |  |  |  |  |  |  |  |  |  |  | |
| mitochondrial import inner membrane translocase subunit tim14-1-like | |  |  |  |  |  |  |  |  |  |  | |
| pectin lyase-like superfamily protein isoform 1 | |  |  |  |  |  |  |  |  |  |  | |
| agamous-like mads-box protein agl8 homolog | |  |  |  |  |  |  |  |  |  |  | |
| gibberellin 20 oxidase 1-a-like | |  |  |  |  |  |  |  |  |  |  | |
| heat shock transcription factor a-3 isoform 1 | |  |  |  |  |  |  |  |  |  |  | |
| light-regulated protein | |  |  |  |  |  |  |  |  |  |  | |
| cytochrome p450 71A4-like | |  |  |  |  |  |  |  |  |  |  | |
| btb and taz domain protein 2 isoform 1 | |  |  |  |  |  |  |  |  |  |  | |
| zinc finger protein constans-like 2 | |  |  |  |  |  |  |  |  |  |  | |
| salt tolerance-like protein at1g78600-like | |  |  |  |  |  |  |  |  |  |  | |
| dof zinc finger | |  |  |  |  |  |  |  |  |  |  | |
| granule-bound starch synthase chloroplastic amyloplastic-like | |  |  |  |  |  |  |  |  |  |  | |
| apoptosis-enhancing nuclease-like | |  |  |  |  |  |  |  |  |  |  | |
| phosphoglucan phosphatase chloroplastic-like isoform x2 | |  |  |  |  |  |  |  |  |  |  | |
| phosphoenolpyruvate phosphate translocator chloroplastic-like | |  |  |  |  |  |  |  |  |  |  | |
| cytochrome p450 family protein | |  |  |  |  |  |  |  |  |  |  | |
| major pollen allergen ory s 1 | |  |  |  |  |  |  |  |  |  |  | |
| primary amine oxidase-like | |  |  |  |  |  |  |  |  |  |  | |
| small heat shock class IV chloroplastic-like | |  |  |  |  |  |  |  |  |  |  | |
| pectinesterase 68-like | |  |  |  |  |  |  |  |  |  |  | |
| myb-like hth transcriptional regulator family | |  |  |  |  |  |  |  |  |  |  | |
| Polygalacturonase precursor | |  |  |  |  |  |  |  |  |  |  | |
| delta-cadinene synthase isozyme | |  |  |  |  |  |  |  |  |  |  | |
| (-)-germacrene d synthase-like | |  |  |  |  |  |  |  |  |  |  | |
| luminal-binding protein 5-like | |  |  |  |  |  |  |  |  |  |  | |
| 70 KDa class I heat shock protein | |  |  |  |  |  |  |  |  |  |  | |
| v-type proton atpase subunit e | |  |  |  |  |  |  |  |  |  |  | |
| cytochrome p450 83B1-like | |  |  |  |  |  |  |  |  |  |  | |
| isoamylase chloroplastic-like | |  |  |  |  |  |  |  |  |  |  | |
| cytochrome p450 71A2-like | |  |  |  |  |  |  |  |  |  |  | |
| purine permease 3-like | |  |  |  |  |  |  |  |  |  |  | |
| cytochrome p450 81D1-like | |  |  |  |  |  |  |  |  |  |  | |
| patatin group a-3 | |  |  |  |  |  |  |  |  |  |  | |
| citrate-binding protein-like | |  |  |  |  |  |  |  |  |  |  | |
| gigantea protein isoform 1 | |  |  |  |  |  |  |  |  |  |  | |
| ammonium transporter 1 member 2-like | |  |  |  |  |  |  |  |  |  |  | |
| nodulin 21 -like transporter family protein isoform 1 | |  |  |  |  |  |  |  |  |  |  | |
| non-symbiotic hemoglobin 2 | |  |  |  |  |  |  |  |  |  |  | |
| bidirectional sugar transporter sweet14 | |  |  |  |  |  |  |  |  |  |  | |
| aluminum-activated malate transporter 8 | |  |  |  |  |  |  |  |  |  |  | |
| extra-large gtp-binding protein 3 | |  |  |  |  |  |  |  |  |  |  | |
| cyanidin-3-o-glucoside 2-o-glucuronosyltransferase-like | |  |  |  |  |  |  |  |  |  |  | |
| udp-glycosyltransferase 87a2-like | |  |  |  |  |  |  |  |  |  |  | |
| maternal effect embryo arrest 59 | |  |  |  |  |  |  |  |  |  |  | |
| zinc finger protein constans-like 4-like | |  |  |  |  |  |  |  |  |  |  | |
| thiazole biosynthetic chloroplastic | |  |  |  |  |  |  |  |  |  |  | |
| ( )-nerolidol synthase 1- partial | |  |  |  |  |  |  |  |  |  |  | |
| epoxide hydrolase 2-like | |  |  |  |  |  |  |  |  |  |  | |
| class I heat shock protein 83-like class I isoform 1 | |  |  |  |  |  |  |  |  |  |  | |
| duf21 domain-containing protein at5g52790-like | |  |  |  |  |  |  |  |  |  |  | |
| kdel-tailed cysteine endopeptidase cep1-like | |  |  |  |  |  |  |  |  |  |  | |
| u-box domain-containing protein 35-like | |  |  |  |  |  |  |  |  |  |  | |
| receptor protein | |  |  |  |  |  |  |  |  |  |  | |
| u-box domain-containing protein kinase family protein isoform 2 | |  |  |  |  |  |  |  |  |  |  | |
| wrky transcription factor 27-like | |  |  |  |  |  |  |  |  |  |  | |
| phosphatidylinositide phosphatase sac1-like | |  |  |  |  |  |  |  |  |  |  | |
| casp-like protein poptrdraft_798217-like | |  |  |  |  |  |  |  |  |  |  | |
| (-)-germacrene d synthase | |  |  |  |  |  |  |  |  |  |  | |
| methyl esterase isoform 1 | |  |  |  |  |  |  |  |  |  |  | |
| hsp20 heat shock protein-α crystallin | |  |  |  |  |  |  |  |  |  |  | |
| nodulin 21 -like transporter family protein isoform partial | |  |  |  |  |  |  |  |  |  |  | |
| u-box domain-containing protein 15-like | |  |  |  |  |  |  |  |  |  |  | |
| inorganic phosphate transporter 1-9-like | |  |  |  |  |  |  |  |  |  |  | |
| f-box protein at2g02240-like | |  |  |  |  |  |  |  |  |  |  | |
| methyl esterase | |  |  |  |  |  |  |  |  |  |  | |
| pathogenesis-related protein sth-2-like | |  |  |  |  |  |  |  |  |  |  | |
| phospholipase a1- chloroplastic-like | |  |  |  |  |  |  |  |  |  |  | |
| phospholipase a1- chloroplastic-like | |  |  |  |  |  |  |  |  |  |  | |
| cytochrome family subfamily polypeptide | |  |  |  |  |  |  |  |  |  |  | |
| cytosolic class II small heat shock protein hct2 | |  |  |  |  |  |  |  |  |  |  | |
| histidine-containing phosphotransfer protein 4-like | |  |  |  |  |  |  |  |  |  |  | |
| dehydration-responsive element-binding protein 1b-like | |  |  |  |  |  |  |  |  |  |  | |
| ribulose bisphosphate carboxylase oxygenase chloroplastic-like | |  |  |  |  |  |  |  |  |  |  | |
| regulator of chrom. condensation repeat-containing protein isoform 1 | |  |  |  |  |  |  |  |  |  |  | |
| late blight resistance protein homolog r1a-10-like | |  |  |  |  |  |  |  |  |  |  | |
| u-box domain-containing protein 43-like | |  |  |  |  |  |  |  |  |  |  | |
| serine-threonine protein plant- | |  |  |  |  |  |  |  |  |  |  | |
| monoacylglycerol lipase abhd6-a-like | |  |  |  |  |  |  |  |  |  |  | |
| brassinosteroid-regulated protein bru1-like | |  |  |  |  |  |  |  |  |  |  | |
| late embryogenesis abundant group 3-like | |  |  |  |  |  |  |  |  |  |  | |
| pectin lyase-like superfamily protein isoform 1 | |  |  |  |  |  |  |  |  |  |  | |
| chaperone protein dnaj 6-like | |  |  |  |  |  |  |  |  |  |  | |
| cytochrome p450 71B1-like | |  |  |  |  |  |  |  |  |  |  | |
| xyloglucan endotransglucosylase hydrolase protein 31 precursor | |  |  |  |  |  |  |  |  |  |  | |
| subtilisin-like protease-like | |  |  |  |  |  |  |  |  |  |  | |
| protein lhy-like | |  |  |  |  |  |  |  |  |  |  | |
| major allergen pru ar 1-like | |  |  |  |  |  |  |  |  |  |  | |
| dnaj homolog subfamily b member 3-like | |  |  |  |  |  |  |  |  |  |  | |
| pleiotropic drug resistance 9 | |  |  |  |  |  |  |  |  |  |  | |
| udp-glucosyl transferase 85a2 | |  |  |  |  |  |  |  |  |  |  | |
| nac domain-containing protein 94 | |  |  |  |  |  |  |  |  |  |  | |
| expansin beta | |  |  |  |  |  |  |  |  |  |  | |
| transcription factor asg4-like | |  |  |  |  |  |  |  |  |  |  | |
| ribulose bisphosphate carboxylase oxygenase chloroplastic-like | |  |  |  |  |  |  |  |  |  |  | |
| rcc1 domain-containing protein ddb_g0279253-like isoform x1 | |  |  |  |  |  |  |  |  |  |  | |
| haloacid dehalogenase-like hydrolase domain-containing protein 3-like | |  |  |  |  |  |  |  |  |  |  | |
| cytochrome p450 76C4-like | |  |  |  |  |  |  |  |  |  |  | |
| secologanin synthase-like | |  |  |  |  |  |  |  |  |  |  | |
| glutathione s-transferase | |  |  |  |  |  |  |  |  |  |  | |
| xyloglucan endotransglucosylase hydrolase protein 16-like | |  |  |  |  |  |  |  |  |  |  | |
| xyloglucan endotransglucosylase hydrolase protein 16-like | |  |  |  |  |  |  |  |  |  |  | |
| xyloglucan endotransglucosylase hydrolase protein 16-like | |  |  |  |  |  |  |  |  |  |  | |
| lrr receptor-like serine threonine-protein kinase fls2-like | |  |  |  |  |  |  |  |  |  |  | |
| beta-galactosidase 7-like | |  |  |  |  |  |  |  |  |  |  | |
| nac domain containing protein 90 | |  |  |  |  |  |  |  |  |  |  | |
| beta-galactosidase 7-like | |  |  |  |  |  |  |  |  |  |  | |
| subtilisin-like protease-like | |  |  |  |  |  |  |  |  |  |  | |
| hxxxd-type acyl-transferase family | |  |  |  |  |  |  |  |  |  |  | |
| wat1-related protein at4g30420-like | |  |  |  |  |  |  |  |  |  |  | |
| epidermis-specific secreted glycoprotein ep1-like | |  |  |  |  |  |  |  |  |  |  | |
| calcium-binding protein cml44-like | |  |  |  |  |  |  |  |  |  |  | |
| ethylene-responsive transcription factor erf017-like | |  |  |  |  |  |  |  |  |  |  | |
| auxin-induced protein 10a5-like | |  |  |  |  |  |  |  |  |  |  | |
| dna binding | |  |  |  |  |  |  |  |  |  |  | |
| histidine phosphotransfer protein | |  |  |  |  |  |  |  |  |  |  | |
| udp-glycosyltransferase 85a2-like | |  |  |  |  |  |  |  |  |  |  | |
| udp-glycosyltransferase 90a1-like | |  |  |  |  |  |  |  |  |  |  | |
| auxin-binding protein abp19a-like | |  |  |  |  |  |  |  |  |  |  | |
| ethylene-responsive transcription factor erf109 | |  |  |  |  |  |  |  |  |  |  | |
| pentatricopeptide repeat-containing protein at5g27110-like | |  |  |  |  |  |  |  |  |  |  | |
| clathrin assembly protein at4g40080-like | |  |  |  |  |  |  |  |  |  |  | |
| udp-glycosyltransferase 91c1-like | |  |  |  |  |  |  |  |  |  |  | |
| 70 KDa class I heat shock protein | |  |  |  |  |  |  |  |  |  |  | |
| non-symbiotic hemoglobin 2 ) | |  |  |  |  |  |  |  |  |  |  | |
| germin-like protein 9-3-like | |  |  |  |  |  |  |  |  |  |  | |
| cytochrome p450 86B1-like | |  |  |  |  |  |  |  |  |  |  | |
| aromatic-l-amino-acid decarboxylase-like | |  |  |  |  |  |  |  |  |  |  | |
| dnaj homolog subfamily b member 6-like | |  |  |  |  |  |  |  |  |  |  | |
| heat stress transcription factor a-2 isoform 1 | |  |  |  |  |  |  |  |  |  |  | |
| udp-glycosyltransferase 87a1 | |  |  |  |  |  |  |  |  |  |  | |
| 70 KDa class I heat shock protein | |  |  |  |  |  |  |  |  |  |  | |
| Hsp10-like chaperonin protein | |  |  |  |  |  |  |  |  |  |  | |
| acid invertase | |  |  |  |  |  |  |  |  |  |  | |
| clathrin coat assembly protein ap180-like | |  |  |  |  |  |  |  |  |  |  | |
| cellulose synthase-like protein h1-like isoform x3 | |  |  |  |  |  |  |  |  |  |  | |
| molybdate transporter 1-like | |  |  |  |  |  |  |  |  |  |  | |
| ap2-like ethylene-responsive transcription factor at2g41710-like | |  |  |  |  |  |  |  |  |  |  | |
| oleosin 5-like | |  |  |  |  |  |  |  |  |  |  | |
| adagio protein 3-like | |  |  |  |  |  |  |  |  |  |  | |
| receptor-like protein kinase at3g47110-like | |  |  |  |  |  |  |  |  |  |  | |
| 2-hydroxyisoflavanone dehydratase-like | |  |  |  |  |  |  |  |  |  |  | |
| heat stress transcription factor a-4-like | |  |  |  |  |  |  |  |  |  |  | |
| pleiotropic drug resistance protein 2-like | |  |  |  |  |  |  |  |  |  |  | |
| flavonol 4 –sulfotransferase | |  |  |  |  |  |  |  |  |  |  | |
| tropinone reductase homolog | |  |  |  |  |  |  |  |  |  |  | |
| 90 KDa class I heat shock endoplasmin | |  |  |  |  |  |  |  |  |  |  | |
| abc transporter g family member 23 | |  |  |  |  |  |  |  |  |  |  | |
| low quality protein: cytochrome p450 76C4-like | |  |  |  |  |  |  |  |  |  |  | |
| aquaporin pip2-1 | |  |  |  |  |  |  |  |  |  |  | |
| eg45-like domain containing | |  |  |  |  |  |  |  |  |  |  | |
| spotted leaf | |  |  |  |  |  |  |  |  |  |  | |

| (b) Mature leaves | | | | | | | | | | | |
| --- | --- | --- | --- | --- | --- | --- | --- | --- | --- | --- | --- |
| Description | Time point with fold change of ≥ 5 | F | G | H | L | F | | G | F | G | H |
|  | Time point in comparison | A | | | | G | H | | L | | |
| flavanoid 3',5'-hydroxylase | |  |  |  |  |  |  |  |  |  |  |
| equilibrative nucleoside transporter 4 | |  |  |  |  |  |  |  |  |  |  |
| alpha-crystallin domain isoform 1 | |  |  |  |  |  |  |  |  |  |  |
| 11-oxo-beta-amyrin 30-oxidase-like isoform x1 | |  |  |  |  |  |  |  |  |  |  |
| lrr receptor-like serine threonine-protein kinase at3g47570-like | |  |  |  |  |  |  |  |  |  |  |
| homeodomain-liksuperfamily isoform 1 | |  |  |  |  |  |  |  |  |  |  |
| wall-associated receptor kinase-like 1-like | |  |  |  |  |  |  |  |  |  |  |
| lrr receptor-like serine threonine-protein kinase at3g47570-like | |  |  |  |  |  |  |  |  |  |  |
| sequence-specific dna binding transcription | |  |  |  |  |  |  |  |  |  |  |
| late embryogenesis abundant protein d-34-like | |  |  |  |  |  |  |  |  |  |  |
| nadph--cytochrome p450 reductase-like | |  |  |  |  |  |  |  |  |  |  |
| xyloglucan endotransglucosylase hydrolase protein 23-like | |  |  |  |  |  |  |  |  |  |  |
| kaempferol 3-o-beta-d-galactosyltransferase-like | |  |  |  |  |  |  |  |  |  |  |
| nadp-dependent glyceraldehyde-3-phosphate dehydrogenase-like | |  |  |  |  |  |  |  |  |  |  |
| 90 KDa class I heat shock family protein | |  |  |  |  |  |  |  |  |  |  |
| ammonium transporter 3 member 1-like | |  |  |  |  |  |  |  |  |  |  |
| gamma-tocopherol methyltransferase | |  |  |  |  |  |  |  |  |  |  |
| cyclin A isoform 1 | |  |  |  |  |  |  |  |  |  |  |
| phospholipase a1-iigamma-like | |  |  |  |  |  |  |  |  |  |  |
| zinc finger family protein | |  |  |  |  |  |  |  |  |  |  |
| cytochrome p450 71A1-like | |  |  |  |  |  |  |  |  |  |  |
| cytochrome p450 | |  |  |  |  |  |  |  |  |  |  |
| alpha-expansin 1 family protein | |  |  |  |  |  |  |  |  |  |  |
| dicer-like protein isoform 3 | |  |  |  |  |  |  |  |  |  |  |
| heat shock transcription factor a-3 isoform 1 | |  |  |  |  |  |  |  |  |  |  |
| inositol-3-phosphate synthase | |  |  |  |  |  |  |  |  |  |  |
| protein transparent testa 12-like isoform 2 | |  |  |  |  |  |  |  |  |  |  |
| protein transparent testa 12-like isoform 3 | |  |  |  |  |  |  |  |  |  |  |
| ethylene-responsive transcription factor abr1-like | |  |  |  |  |  |  |  |  |  |  |
| u-box domain-containing protein 26-like | |  |  |  |  |  |  |  |  |  |  |
| monothiol glutaredoxin-s1-like | |  |  |  |  |  |  |  |  |  |  |
| isoflavone 2 -hydroxylase-like | |  |  |  |  |  |  |  |  |  |  |
| protein yls7-like | |  |  |  |  |  |  |  |  |  |  |
| high affinity nitrate transporter –like | |  |  |  |  |  |  |  |  |  |  |
| phosphate transporter pho1 homolog 1 isoform 2 | |  |  |  |  |  |  |  |  |  |  |
| nudix hydrolase 1-like | |  |  |  |  |  |  |  |  |  |  |
| xyloglucan endotransglucosylase hydrolase protein 33-like | |  |  |  |  |  |  |  |  |  |  |
| auxin-induced protein 5ng4-like | |  |  |  |  |  |  |  |  |  |  |
| two-component response regulator arr3-like | |  |  |  |  |  |  |  |  |  |  |
| cucumisin-like | |  |  |  |  |  |  |  |  |  |  |
| phosphate transporter pho1 homolog 10-like | |  |  |  |  |  |  |  |  |  |  |
| alpha- glucan phosphorylase l-2 chloroplastic amyloplastic-like | |  |  |  |  |  |  |  |  |  |  |
| ninja-family protein afp2-like | |  |  |  |  |  |  |  |  |  |  |
| protein transparent testa 12 isoform 4 | |  |  |  |  |  |  |  |  |  |  |
| protein transparent testa 12-like isoform 5 | |  |  |  |  |  |  |  |  |  |  |
| inorganic phosphate transporter 2- chloroplastic-like | |  |  |  |  |  |  |  |  |  |  |
| gdsl esterase lipase at3g26430-like | |  |  |  |  |  |  |  |  |  |  |
| 70 KDa class I heat shock protein | |  |  |  |  |  |  |  |  |  |  |
| hsp20 heat shock protein-α crystalline | |  |  |  |  |  |  |  |  |  |  |
| protein aspartic protease in guard cell 2-like | |  |  |  |  |  |  |  |  |  |  |
| intraflagellar transport particle protein ift140 | |  |  |  |  |  |  |  |  |  |  |
| glutamate receptor | |  |  |  |  |  |  |  |  |  |  |
| receptor-like serine threonine-protein kinase at5g57670-like | |  |  |  |  |  |  |  |  |  |  |
| zinc finger ccch domain-containing protein 48-like | |  |  |  |  |  |  |  |  |  |  |
| shikimate o-hydroxycinnamoyltransferase-like | |  |  |  |  |  |  |  |  |  |  |
| 70 KDa class I heat shock protein | |  |  |  |  |  |  |  |  |  |  |
| cysteine-rich repeat secretory protein 12-like isoform 2 | |  |  |  |  |  |  |  |  |  |  |
| upf0497 membrane protein 8 | |  |  |  |  |  |  |  |  |  |  |
| photosystem ii protein vi | |  |  |  |  |  |  |  |  |  |  |
| strictosidine synthase 1-like | |  |  |  |  |  |  |  |  |  |  |
| mitochondrial import inner membrane translocase subunit tim14-1-like | |  |  |  |  |  |  |  |  |  |  |
| caffeoyl- o-methyltransferase at4g26220-like | |  |  |  |  |  |  |  |  |  |  |
| pectin lyase-like superfamily protein isoform 1 | |  |  |  |  |  |  |  |  |  |  |
| multidrug and toxin extrusion protein 1-like | |  |  |  |  |  |  |  |  |  |  |
| rho guanine nucleotide exchange factor 8-like | |  |  |  |  |  |  |  |  |  |  |
| lysine decarboxylase family protein isoform 2 | |  |  |  |  |  |  |  |  |  |  |
| wuschel-related homeobox 1 | |  |  |  |  |  |  |  |  |  |  |
| pathogenesis-related thaumatin superfamily protein | |  |  |  |  |  |  |  |  |  |  |
| wd repeat and hmg-box dna-binding protein 1-like | |  |  |  |  |  |  |  |  |  |  |
| flavonol 4 -sulfotransferase-like | |  |  |  |  |  |  |  |  |  |  |
| two-component response regulator-like aprr1-like | |  |  |  |  |  |  |  |  |  |  |
| l-type lectin-domain containing receptor kinase –like | |  |  |  |  |  |  |  |  |  |  |
| wrky transcription factor 50-like | |  |  |  |  |  |  |  |  |  |  |
| aquaporin tip2-1 | |  |  |  |  |  |  |  |  |  |  |
| dna photolyase family protein isoform 3 | |  |  |  |  |  |  |  |  |  |  |
| ethylene-responsive transcription factor win1-like | |  |  |  |  |  |  |  |  |  |  |
| adenine phosphoribosyltransferase 2 family protein | |  |  |  |  |  |  |  |  |  |  |
| amino acid binding | |  |  |  |  |  |  |  |  |  |  |
| light-regulated protein | |  |  |  |  |  |  |  |  |  |  |
| late embryogenesis abundant hydroxyproline-rich glycoprotein | |  |  |  |  |  |  |  |  |  |  |
| remorin family protein | |  |  |  |  |  |  |  |  |  |  |
| xylosyltransferase 1-like | |  |  |  |  |  |  |  |  |  |  |
| protein phosphatase | |  |  |  |  |  |  |  |  |  |  |
| cytochrome p450 71A2-like | |  |  |  |  |  |  |  |  |  |  |
| low quality protein: cytochrome p450 71A4-like | |  |  |  |  |  |  |  |  |  |  |
| cytochrome p450 71A2-like | |  |  |  |  |  |  |  |  |  |  |
| cytochrome p450 71A4-like | |  |  |  |  |  |  |  |  |  |  |
| btb and taz domain protein 2 isoform 1 | |  |  |  |  |  |  |  |  |  |  |
| alanyl-trna synthetase | |  |  |  |  |  |  |  |  |  |  |
| zinc finger protein constans-like 2 | |  |  |  |  |  |  |  |  |  |  |
| protein da1-related 1-like | |  |  |  |  |  |  |  |  |  |  |
| salicylic acid-binding protein 2-like | |  |  |  |  |  |  |  |  |  |  |
| salicylic acid-binding protein 2-like | |  |  |  |  |  |  |  |  |  |  |
| salt tolerance-like protein at1g78600-like | |  |  |  |  |  |  |  |  |  |  |
| 1-aminocyclopropane-1-carboxylate oxidase 5-like | |  |  |  |  |  |  |  |  |  |  |
| 3-epi-6-deoxocathasterone 23-monooxygenase-like isoform x2 | |  |  |  |  |  |  |  |  |  |  |
| mitochondrial chaperone bcs1-like isoform 1 | |  |  |  |  |  |  |  |  |  |  |
| glycosyl hydrolase superfamily protein isoform 1 | |  |  |  |  |  |  |  |  |  |  |
| cytochrome p450 90B1-like | |  |  |  |  |  |  |  |  |  |  |
| tetrahydrocannabinolic acid synthase-like | |  |  |  |  |  |  |  |  |  |  |
| lob domain-containing protein 41 | |  |  |  |  |  |  |  |  |  |  |
| scarecrow-like protein 32 | |  |  |  |  |  |  |  |  |  |  |
| alpha- -glucan-protein synthase | |  |  |  |  |  |  |  |  |  |  |
| upf0301 protein cpha266_0885-like isoform 2 | |  |  |  |  |  |  |  |  |  |  |
| expansin alpha isoform 1 | |  |  |  |  |  |  |  |  |  |  |
| dof zinc finger | |  |  |  |  |  |  |  |  |  |  |
| granule-bound starch synthase chloroplastic amyloplastic-like | |  |  |  |  |  |  |  |  |  |  |
| myb-related protein 306 isoform 1 | |  |  |  |  |  |  |  |  |  |  |
| e2f transcription factor-like e2fe | |  |  |  |  |  |  |  |  |  |  |
| serine threonine-protein kinase ht1-like | |  |  |  |  |  |  |  |  |  |  |
| quinone oxidoreductase-like protein chloroplastic-like | |  |  |  |  |  |  |  |  |  |  |
| apoptosis-enhancing nuclease-like | |  |  |  |  |  |  |  |  |  |  |
| l-aspartate oxidase 1-like | |  |  |  |  |  |  |  |  |  |  |
| phosphoglucan phosphatase chloroplastic-like isoform x2 | |  |  |  |  |  |  |  |  |  |  |
| phosphoenolpyruvate phosphate translocator chloroplastic-like | |  |  |  |  |  |  |  |  |  |  |
| cytochrome p450 family protein | |  |  |  |  |  |  |  |  |  |  |
| major pollen allergen ory s 1 | |  |  |  |  |  |  |  |  |  |  |
| h+-atpase family protein | |  |  |  |  |  |  |  |  |  |  |
| c4-dicarboxylate transporter malic acid transport protein isoform 1 | |  |  |  |  |  |  |  |  |  |  |
| copper amine oxidase family protein | |  |  |  |  |  |  |  |  |  |  |
| copper amine oxidase family protein | |  |  |  |  |  |  |  |  |  |  |
| acid beta-fructofuranosidase-like | |  |  |  |  |  |  |  |  |  |  |
| small class III heat shock chloroplastic-like | |  |  |  |  |  |  |  |  |  |  |
| pectinesterase 68-like | |  |  |  |  |  |  |  |  |  |  |
| delta-cadinene synthase isozyme | |  |  |  |  |  |  |  |  |  |  |
| (-)-germacrene d synthase | |  |  |  |  |  |  |  |  |  |  |
| (-)-germacrene d synthase-like | |  |  |  |  |  |  |  |  |  |  |
| luminal-binding protein 5-like | |  |  |  |  |  |  |  |  |  |  |
| late embryogenesis abundant | |  |  |  |  |  |  |  |  |  |  |
| phosphatidylethanolamine binding | |  |  |  |  |  |  |  |  |  |  |
| sec14 cytosolic factor family protein | |  |  |  |  |  |  |  |  |  |  |
| v-type proton atpase subunit e | |  |  |  |  |  |  |  |  |  |  |
| cytochrome p450 83B1-like | |  |  |  |  |  |  |  |  |  |  |
| transferring glycosyl | |  |  |  |  |  |  |  |  |  |  |
| pxmp2 4 family protein 4-like | |  |  |  |  |  |  |  |  |  |  |
| sterol regulatory element-binding protein site 2 | |  |  |  |  |  |  |  |  |  |  |
| histidine-containing phosphotransfer protein 1 | |  |  |  |  |  |  |  |  |  |  |
| receptor-like serine threonine-protein kinase ale2-like | |  |  |  |  |  |  |  |  |  |  |
| cyclin D6 isoform 2 | |  |  |  |  |  |  |  |  |  |  |
| luminal-binding protein 5-like | |  |  |  |  |  |  |  |  |  |  |
| Lipoxygenase | |  |  |  |  |  |  |  |  |  |  |
| 50s ribosomal protein l15 | |  |  |  |  |  |  |  |  |  |  |
| cytokinin oxidase 5 | |  |  |  |  |  |  |  |  |  |  |
| udp-glycosyltransferase 85a1 isoform 1 | |  |  |  |  |  |  |  |  |  |  |
| isoflavone 2 -hydroxylase-like | |  |  |  |  |  |  |  |  |  |  |
| mads-box transcription factor 26 | |  |  |  |  |  |  |  |  |  |  |
| transcription factor myb39-like | |  |  |  |  |  |  |  |  |  |  |
| purine permease 3 | |  |  |  |  |  |  |  |  |  |  |
| folic acid and derivative biosynthetic process | |  |  |  |  |  |  |  |  |  |  |
| kinase interacting family | |  |  |  |  |  |  |  |  |  |  |
| oligopeptide transporter 4-like | |  |  |  |  |  |  |  |  |  |  |
| cinnamyl alcohol dehydrogenase 9 | |  |  |  |  |  |  |  |  |  |  |
| protein eceriferum 1-like | |  |  |  |  |  |  |  |  |  |  |
| cyclin A3-like isoform 1 | |  |  |  |  |  |  |  |  |  |  |
| abc transporter b family member 13-like | |  |  |  |  |  |  |  |  |  |  |
| cytochrome p450 81D1-like | |  |  |  |  |  |  |  |  |  |  |
| isoaspartyl peptidase l-asparaginase 2-like | |  |  |  |  |  |  |  |  |  |  |
| patatin group a-3 | |  |  |  |  |  |  |  |  |  |  |
| b3 domain-containing protein at3g19184-like | |  |  |  |  |  |  |  |  |  |  |
| serrate rna effector molecule homolog | |  |  |  |  |  |  |  |  |  |  |
| atp sulfurylase 2 | |  |  |  |  |  |  |  |  |  |  |
| gigantea protein isoform 1 | |  |  |  |  |  |  |  |  |  |  |
| inositol- -triphosphate-5-phosphatase | |  |  |  |  |  |  |  |  |  |  |
| inositol transporter 2 isoform 1 | |  |  |  |  |  |  |  |  |  |  |
| ammonium transporter 1 member 2-like | |  |  |  |  |  |  |  |  |  |  |
| tyrosine-specific transport | |  |  |  |  |  |  |  |  |  |  |
| chlorophyll a-b binding protein chloroplastic-like | |  |  |  |  |  |  |  |  |  |  |
| aromatic-l-amino-acid decarboxylase-like | |  |  |  |  |  |  |  |  |  |  |
| pollen ole e 1 allergen and extensin family protein | |  |  |  |  |  |  |  |  |  |  |
| galactinol synthase family protein | |  |  |  |  |  |  |  |  |  |  |
| lob domain-containing | |  |  |  |  |  |  |  |  |  |  |
| myb-related protein myb4-like | |  |  |  |  |  |  |  |  |  |  |
| lysine-specific demethylase 8-like | |  |  |  |  |  |  |  |  |  |  |
| epidermal patterning factor-like protein 9-like | |  |  |  |  |  |  |  |  |  |  |
| extra-large gtp-binding protein 3 | |  |  |  |  |  |  |  |  |  |  |
| cyanidin-3-o-glucoside 2-o-glucuronosyltransferase-like | |  |  |  |  |  |  |  |  |  |  |
| nodulin-related family protein | |  |  |  |  |  |  |  |  |  |  |
| udp-glycosyltransferase 87a2-like | |  |  |  |  |  |  |  |  |  |  |
| acyl- n-acyltransferases superfamily protein | |  |  |  |  |  |  |  |  |  |  |
| maternal effect embryo arrest 59 | |  |  |  |  |  |  |  |  |  |  |
| zinc finger protein constans-like 4-like | |  |  |  |  |  |  |  |  |  |  |
| adp glucose pyrophosphorylase 1 | |  |  |  |  |  |  |  |  |  |  |
| pectinesterase inhibitor 18-like | |  |  |  |  |  |  |  |  |  |  |
| thiazole biosynthetic chloroplastic | |  |  |  |  |  |  |  |  |  |  |
| ( )-nerolidol synthase chloroplastic-like | |  |  |  |  |  |  |  |  |  |  |
| quinone reductase family protein | |  |  |  |  |  |  |  |  |  |  |
| myosin 2 isoform 1 | |  |  |  |  |  |  |  |  |  |  |
| serine carboxypeptidase-like 31-like | |  |  |  |  |  |  |  |  |  |  |
| epoxide hydrolase 2-like | |  |  |  |  |  |  |  |  |  |  |
| beta-carotene hydroxylase chloroplastic isoform 1 | |  |  |  |  |  |  |  |  |  |  |
| class I heat shock protein 83-like isoform 1 | |  |  |  |  |  |  |  |  |  |  |
| duf21 domain-containing protein at5g52790-like | |  |  |  |  |  |  |  |  |  |  |
| bidirectional sugar transporter sweet3 | |  |  |  |  |  |  |  |  |  |  |
| probable aquaporin pip1-2 | |  |  |  |  |  |  |  |  |  |  |
| oxoglutarate 3-dioxygenase-like | |  |  |  |  |  |  |  |  |  |  |
| tetratricopeptide repeat-like superfamily protein isoform 1 | |  |  |  |  |  |  |  |  |  |  |
| kdel-tailed cysteine endopeptidase cep1-like | |  |  |  |  |  |  |  |  |  |  |
| kdel-tailed cysteine endopeptidase cep1-like | |  |  |  |  |  |  |  |  |  |  |
| transcription factor laf1-like | |  |  |  |  |  |  |  |  |  |  |
| pectin methylesterase family protein | |  |  |  |  |  |  |  |  |  |  |
| trehalose-phosphate phosphatase a-like isoform x5 | |  |  |  |  |  |  |  |  |  |  |
| u-box domain-containing protein 35-like | |  |  |  |  |  |  |  |  |  |  |
| receptor protein | |  |  |  |  |  |  |  |  |  |  |
| u-box domain-containing protein kinase family protein isoform 2 | |  |  |  |  |  |  |  |  |  |  |
| cyclin-dependent protein kinase class f4-like | |  |  |  |  |  |  |  |  |  |  |
| anaphase-promoting complex subunit cdc20-like | |  |  |  |  |  |  |  |  |  |  |
| protein yls7-like | |  |  |  |  |  |  |  |  |  |  |
| gdsl esterase lipase at1g29670-like | |  |  |  |  |  |  |  |  |  |  |
| phosphatidylinositide phosphatase sac1-like | |  |  |  |  |  |  |  |  |  |  |
| 10 KDa chaperonin-like protein isoform 1 | |  |  |  |  |  |  |  |  |  |  |
| pplz02 family protein | |  |  |  |  |  |  |  |  |  |  |
| molybdate transporter 1-like | |  |  |  |  |  |  |  |  |  |  |
| methyl esterase isoform 1 | |  |  |  |  |  |  |  |  |  |  |
| boron transporter 4 | |  |  |  |  |  |  |  |  |  |  |
| inositol transporter 1-like | |  |  |  |  |  |  |  |  |  |  |
| auxin efflux carrier family protein isoform 1 | |  |  |  |  |  |  |  |  |  |  |
| glutamate receptor –like | |  |  |  |  |  |  |  |  |  |  |
| catalase isozyme 1 isoform 1 | |  |  |  |  |  |  |  |  |  |  |
| butyrate-- ligase peroxisomal-like | |  |  |  |  |  |  |  |  |  |  |
| cyclic dof factor 3-like | |  |  |  |  |  |  |  |  |  |  |
| heat shock transcription factor b-1 isoform 1 | |  |  |  |  |  |  |  |  |  |  |
| inorganic pyrophosphatase 1-like | |  |  |  |  |  |  |  |  |  |  |
| nodulin 21 -like transporter family protein isoform partial | |  |  |  |  |  |  |  |  |  |  |
| mlo protein homolog 1-like | |  |  |  |  |  |  |  |  |  |  |
| protein cup-shaped cotyledon 3-like | |  |  |  |  |  |  |  |  |  |  |
| f-box protein at2g02240-like | |  |  |  |  |  |  |  |  |  |  |
| early light-induced | |  |  |  |  |  |  |  |  |  |  |
| methyl esterase | |  |  |  |  |  |  |  |  |  |  |
| pathogenesis-related protein sth-2-like | |  |  |  |  |  |  |  |  |  |  |
| calcium-transporting atpase plasma membrane-type-like | |  |  |  |  |  |  |  |  |  |  |
| protein wvd2-like 1-like isoform x2 | |  |  |  |  |  |  |  |  |  |  |
| ethylene receptor 2-like | |  |  |  |  |  |  |  |  |  |  |
| cytochrome family subfamily polypeptide | |  |  |  |  |  |  |  |  |  |  |
| cytochrome family subfamily polypeptide | |  |  |  |  |  |  |  |  |  |  |
| cytosolic class II small heat shock protein hct2 | |  |  |  |  |  |  |  |  |  |  |
| wrky transcription factor 27-like isoform x1 | |  |  |  |  |  |  |  |  |  |  |
| heat shock transcription factor a-2 isoform 1 | |  |  |  |  |  |  |  |  |  |  |
| histidine-containing phosphotransfer protein 4-like | |  |  |  |  |  |  |  |  |  |  |
| mitochondrial carnitine acylcarnitine carrier-like family protein | |  |  |  |  |  |  |  |  |  |  |
| proton-dependent oligopeptide transport family protein | |  |  |  |  |  |  |  |  |  |  |
| drug metabolite transporter superfamily protein | |  |  |  |  |  |  |  |  |  |  |
| cation h(+) antiporter 18-like | |  |  |  |  |  |  |  |  |  |  |
| 1-aminocyclopropane-1-carboxylate synthase-like | |  |  |  |  |  |  |  |  |  |  |
| dehydration-responsive element-binding protein 1b-like | |  |  |  |  |  |  |  |  |  |  |
| cytochrome family subfamily polypeptide 6 | |  |  |  |  |  |  |  |  |  |  |
| endoglucanase 6-like | |  |  |  |  |  |  |  |  |  |  |
| l-type lectin-domain containing receptor kinase –like | |  |  |  |  |  |  |  |  |  |  |
| ribulose bisphosphate carboxylase oxygenase chloroplastic-like | |  |  |  |  |  |  |  |  |  |  |
| regulator of chrom. condensation repeat-containing protein isoform 1 | |  |  |  |  |  |  |  |  |  |  |
| serine carboxypeptidase ii-3 | |  |  |  |  |  |  |  |  |  |  |
| rho gdp-dissociation inhibitor 1-like | |  |  |  |  |  |  |  |  |  |  |
| zinc finger protein constans-like 16 | |  |  |  |  |  |  |  |  |  |  |
| late blight resistance protein homolog r1a-10-like | |  |  |  |  |  |  |  |  |  |  |
| myb-related protein myb4-like | |  |  |  |  |  |  |  |  |  |  |
| u-box domain-containing protein 43-like | |  |  |  |  |  |  |  |  |  |  |
| monoacylglycerol lipase abhd6-a-like | |  |  |  |  |  |  |  |  |  |  |
| pectinesterase 67-like | |  |  |  |  |  |  |  |  |  |  |
| outer envelope pore protein 16- chloroplastic-like | |  |  |  |  |  |  |  |  |  |  |
| brassinosteroid-regulated protein bru1-like | |  |  |  |  |  |  |  |  |  |  |
| peptidyl-prolyl cis-trans isomerase fkbp65-like | |  |  |  |  |  |  |  |  |  |  |
| long chain acyl- synthetase peroxisomal | |  |  |  |  |  |  |  |  |  |  |
| glutathione s-transferase | |  |  |  |  |  |  |  |  |  |  |
| sugar transporter erd6-like 16-like | |  |  |  |  |  |  |  |  |  |  |
| late embryogenesis abundant group 3-like | |  |  |  |  |  |  |  |  |  |  |
| ring-h2 zinc finger protein rha4a | |  |  |  |  |  |  |  |  |  |  |
| serine carboxypeptidase s10 family protein | |  |  |  |  |  |  |  |  |  |  |
| chaperone protein dnaj 6-like | |  |  |  |  |  |  |  |  |  |  |
| phenylalanine ammonia-lyase- partial | |  |  |  |  |  |  |  |  |  |  |
| phenylalanine ammonia-lyase | |  |  |  |  |  |  |  |  |  |  |
| aluminum-activated malate transporter 2 | |  |  |  |  |  |  |  |  |  |  |
| tonoplast intrinsic protein | |  |  |  |  |  |  |  |  |  |  |
| subtilisin-like protease-like | |  |  |  |  |  |  |  |  |  |  |
| protein lhy-like | |  |  |  |  |  |  |  |  |  |  |
| glycine-rich rna-binding protein 7-like isoform x2 | |  |  |  |  |  |  |  |  |  |  |
| 4-alpha-glucanotransferase -related family protein | |  |  |  |  |  |  |  |  |  |  |
| protein transparent testa 12-like isoform 1 | |  |  |  |  |  |  |  |  |  |  |
| dnaj homolog subfamily b member 3-like | |  |  |  |  |  |  |  |  |  |  |
| (-)-alpha-terpineol synthase | |  |  |  |  |  |  |  |  |  |  |
| pleiotropic drug resistance 9 | |  |  |  |  |  |  |  |  |  |  |
| ferric reduction oxidase chloroplastic-like | |  |  |  |  |  |  |  |  |  |  |
| sodium metabolite cotransporter chloroplastic-like | |  |  |  |  |  |  |  |  |  |  |
| chloroplast-targeted copper chaperone protein | |  |  |  |  |  |  |  |  |  |  |
| beta-xylosidase alpha-l-arabinofuranosidase 2-like | |  |  |  |  |  |  |  |  |  |  |
| dentin sialophospho isoform x2 | |  |  |  |  |  |  |  |  |  |  |
| lysine histidine transporter-like 8-like | |  |  |  |  |  |  |  |  |  |  |
| transporter mch1-like | |  |  |  |  |  |  |  |  |  |  |
| abc transporter c family member 9-like | |  |  |  |  |  |  |  |  |  |  |
| dna replication atp-dependent helicase nuclease dna2-like | |  |  |  |  |  |  |  |  |  |  |
| expansin beta | |  |  |  |  |  |  |  |  |  |  |
| gata transcription factor 4-like | |  |  |  |  |  |  |  |  |  |  |
| nad -binding rossmann-fold superfamily protein | |  |  |  |  |  |  |  |  |  |  |
| b-box type zinc finger family protein | |  |  |  |  |  |  |  |  |  |  |
| polyol monosaccharide transporter 5 | |  |  |  |  |  |  |  |  |  |  |
| g2 mitotic-specific cyclin A isoform 1 | |  |  |  |  |  |  |  |  |  |  |
| tubulin beta-5 chain-like | |  |  |  |  |  |  |  |  |  |  |
| transcription factor asg4-like | |  |  |  |  |  |  |  |  |  |  |
| wd repeat-containing protein 44-like | |  |  |  |  |  |  |  |  |  |  |
| stabilizer of iron transporter polynucleotidyl transferase isoform 1 | |  |  |  |  |  |  |  |  |  |  |
| ribulose bisphosphate carboxylase oxygenase chloroplastic-like | |  |  |  |  |  |  |  |  |  |  |
| rcc1 domain-containing protein ddb_g0279253-like isoform x1 | |  |  |  |  |  |  |  |  |  |  |
| centromere-associated protein e-like isoform x1 | |  |  |  |  |  |  |  |  |  |  |
| kinesin motor family protein | |  |  |  |  |  |  |  |  |  |  |
| haloacid dehalogenase-like hydrolase domain-containing protein 3-like | |  |  |  |  |  |  |  |  |  |  |
| condensin complex subunit 1-like | |  |  |  |  |  |  |  |  |  |  |
| protein gast1-like | |  |  |  |  |  |  |  |  |  |  |
| heat stress transcription factor a-4-like | |  |  |  |  |  |  |  |  |  |  |
| polyol transporter 5-like | |  |  |  |  |  |  |  |  |  |  |
| cytochrome p450 93A1-like | |  |  |  |  |  |  |  |  |  |  |
| cytochrome p450 76C4-like | |  |  |  |  |  |  |  |  |  |  |
| soybean seed maturation polypeptides | |  |  |  |  |  |  |  |  |  |  |
| alpha carbonic anhydrase | |  |  |  |  |  |  |  |  |  |  |
| aquaporin pip2-1 isoform 1 | |  |  |  |  |  |  |  |  |  |  |
| casp-like protein vit_19s0090g00570-like | |  |  |  |  |  |  |  |  |  |  |
| transcription factor, putative | |  |  |  |  |  |  |  |  |  |  |
| beta-glucosidase 18-like | |  |  |  |  |  |  |  |  |  |  |
| protochlorophyllide chloroplastic-like | |  |  |  |  |  |  |  |  |  |  |
| tmv resistance protein n-like | |  |  |  |  |  |  |  |  |  |  |
| protein spa1-related 3-like isoform x1 | |  |  |  |  |  |  |  |  |  |  |
| early nodulin-like protein 2-like isoform x2 | |  |  |  |  |  |  |  |  |  |  |
| basic-leucine zipper transcription factor family protein | |  |  |  |  |  |  |  |  |  |  |
| atp binding | |  |  |  |  |  |  |  |  |  |  |
| lrr receptor-like serine threonine-protein kinase at1g56140-like | |  |  |  |  |  |  |  |  |  |  |
| secologanin synthase-like (cyp71A1) | |  |  |  |  |  |  |  |  |  |  |
| glutathione s-transferase para-like | |  |  |  |  |  |  |  |  |  |  |
| glutathione s-transferase | |  |  |  |  |  |  |  |  |  |  |
| protein kinase pinoid 2-like | |  |  |  |  |  |  |  |  |  |  |
| kinesin motor family protein | |  |  |  |  |  |  |  |  |  |  |
| TRNA-methyltransferase | |  |  |  |  |  |  |  |  |  |  |
| xyloglucan endotransglucosylase hydrolase protein 16-like | |  |  |  |  |  |  |  |  |  |  |
| xyloglucan endotransglucosylase hydrolase protein 16-like | |  |  |  |  |  |  |  |  |  |  |
| xyloglucan endotransglucosylase hydrolase protein 16-like | |  |  |  |  |  |  |  |  |  |  |
| xyloglucan endotransglucosylase hydrolase protein 16-like | |  |  |  |  |  |  |  |  |  |  |
| copper ion binding | |  |  |  |  |  |  |  |  |  |  |
| lrr receptor-like serine threonine-protein kinase fls2-like | |  |  |  |  |  |  |  |  |  |  |
| beta-galactosidase 7-like | |  |  |  |  |  |  |  |  |  |  |
| protein heading date 3a-like | |  |  |  |  |  |  |  |  |  |  |
| nac domain containing protein 90 | |  |  |  |  |  |  |  |  |  |  |
| protein lurp-one-related 6-like | |  |  |  |  |  |  |  |  |  |  |
| ethylene-responsive transcription factor erf017-like | |  |  |  |  |  |  |  |  |  |  |
| plant invertase pectin methylesterase inhibitor superfamily | |  |  |  |  |  |  |  |  |  |  |
| hxxxd-type acyl-transferase family | |  |  |  |  |  |  |  |  |  |  |
| embryonic abundant | |  |  |  |  |  |  |  |  |  |  |
| udp-glycosyltransferase 82a1-like | |  |  |  |  |  |  |  |  |  |  |
| transcription factor rf2a-like | |  |  |  |  |  |  |  |  |  |  |
| carboxylesterase 7-like | |  |  |  |  |  |  |  |  |  |  |
| carboxylesterase 12-like | |  |  |  |  |  |  |  |  |  |  |
| wat1-related protein at4g30420-like | |  |  |  |  |  |  |  |  |  |  |
| hydroxycinnamoyl-coenzyme a shikimate quinate hydroxycinnamoyltransferase-like | |  |  |  |  |  |  |  |  |  |  |
| ap2 erf domain-containing transcription | |  |  |  |  |  |  |  |  |  |  |
| 90 KDa class I heat shock protein | |  |  |  |  |  |  |  |  |  |  |
| type i inositol -trisphosphate 5-phosphatase cvp2-like | |  |  |  |  |  |  |  |  |  |  |
| epidermis-specific secreted glycoprotein ep1 | |  |  |  |  |  |  |  |  |  |  |
| polygalacturonase-1 non-catalytic subunit beta-like | |  |  |  |  |  |  |  |  |  |  |
| galactosyl transferase gma12 mnn10 family protein | |  |  |  |  |  |  |  |  |  |  |
| restriction endonuclease | |  |  |  |  |  |  |  |  |  |  |
| 70 KDa class I heat shock protein | |  |  |  |  |  |  |  |  |  |  |
| omega-hydroxypalmitate o-feruloyl transferase-like | |  |  |  |  |  |  |  |  |  |  |
| aromatic-l-amino-acid decarboxylase-like | |  |  |  |  |  |  |  |  |  |  |
| methylecgonone reductase-like | |  |  |  |  |  |  |  |  |  |  |
| e3 ubiquitin-protein ligase atl42 | |  |  |  |  |  |  |  |  |  |  |
| leucine-rich repeat extensin-like protein 3-like | |  |  |  |  |  |  |  |  |  |  |
| ethylene-responsive transcriptional coactivator family protein | |  |  |  |  |  |  |  |  |  |  |
| cobra-like extracellular glycosyl-phosphatidyl inositol-anchored protein family | |  |  |  |  |  |  |  |  |  |  |
| dna binding | |  |  |  |  |  |  |  |  |  |  |
| integrase-type dna-binding superfamily protein | |  |  |  |  |  |  |  |  |  |  |
| calcium-binding protein cml38-like | |  |  |  |  |  |  |  |  |  |  |
| ethylene-responsive transcription factor 4-like | |  |  |  |  |  |  |  |  |  |  |
| short-chain dehydrogenase tic chloroplastic-like | |  |  |  |  |  |  |  |  |  |  |
| high-affinity nitrate transporter | |  |  |  |  |  |  |  |  |  |  |
| udp-glycosyltransferase 90a1-like | |  |  |  |  |  |  |  |  |  |  |
| auxin-binding protein abp19a-like | |  |  |  |  |  |  |  |  |  |  |
| ethylene-responsive transcription factor 5-like | |  |  |  |  |  |  |  |  |  |  |
| alpha-expansin 7 | |  |  |  |  |  |  |  |  |  |  |
| 14 kda proline-rich protein isoform 1 | |  |  |  |  |  |  |  |  |  |  |
| 14 kda proline-rich | |  |  |  |  |  |  |  |  |  |  |
| oleosin 1-like | |  |  |  |  |  |  |  |  |  |  |
| kinase protein with adenine nucleotide alpha hydrolases-like isoform 1 | |  |  |  |  |  |  |  |  |  |  |
| ethylene-responsive transcription factor erf109 | |  |  |  |  |  |  |  |  |  |  |
| udp-glycosyltransferase 85a3-like | |  |  |  |  |  |  |  |  |  |  |
| udp-glycosyltransferase 91c1-like | |  |  |  |  |  |  |  |  |  |  |
| f-box family | |  |  |  |  |  |  |  |  |  |  |
| 90 KDa class I heat shock protein | |  |  |  |  |  |  |  |  |  |  |
| two-pore potassium channel 1-like | |  |  |  |  |  |  |  |  |  |  |
| protein tify 5a | |  |  |  |  |  |  |  |  |  |  |
| germin-like protein 9-3-like | |  |  |  |  |  |  |  |  |  |  |
| cytochrome p450 86B1-like | |  |  |  |  |  |  |  |  |  |  |
| glucose-6-phosphate phosphate translocator chloroplastic-like | |  |  |  |  |  |  |  |  |  |  |
| dnaj homolog subfamily b member 6-like | |  |  |  |  |  |  |  |  |  |  |
| zerumbone synthase-like isoform x2 | |  |  |  |  |  |  |  |  |  |  |
| eid1-like f-box protein 3-like | |  |  |  |  |  |  |  |  |  |  |
| ethylene-responsive transcription factor 1b | |  |  |  |  |  |  |  |  |  |  |
| ethylene-responsive transcription factor erf098-like | |  |  |  |  |  |  |  |  |  |  |
| auxin-induced in root cultures protein 12-like | |  |  |  |  |  |  |  |  |  |  |
| defective in induced resistance 1 protein | |  |  |  |  |  |  |  |  |  |  |
| ring-h2 finger protein | |  |  |  |  |  |  |  |  |  |  |
| sos3-interacting protein 4 | |  |  |  |  |  |  |  |  |  |  |
| udp-glycosyltransferase 87a1 | |  |  |  |  |  |  |  |  |  |  |
| peptide transporter ptr1-like | |  |  |  |  |  |  |  |  |  |  |
| 90 KDa class I heat shock protein | |  |  |  |  |  |  |  |  |  |  |
| sulfite exporter family | |  |  |  |  |  |  |  |  |  |  |
| rna polymerase sigma factor chloroplastic mitochondrial-like | |  |  |  |  |  |  |  |  |  |  |
| hsp20-like chaperones superfamily protein | |  |  |  |  |  |  |  |  |  |  |
| 90 KDa class I heat shock protein | |  |  |  |  |  |  |  |  |  |  |
| cbl-interacting protein kinase 5-like | |  |  |  |  |  |  |  |  |  |  |
| gdsl-motif lipase hydrolase family protein | |  |  |  |  |  |  |  |  |  |  |
| ethylene-responsive transcription factor 12-like | |  |  |  |  |  |  |  |  |  |  |
| transmembrane protein 161a-like | |  |  |  |  |  |  |  |  |  |  |
| acid invertase | |  |  |  |  |  |  |  |  |  |  |
| wrky dna-binding protein isoform 2 | |  |  |  |  |  |  |  |  |  |  |
| thymidine kinase-like | |  |  |  |  |  |  |  |  |  |  |
| molybdate transporter 1-like | |  |  |  |  |  |  |  |  |  |  |
| probable peptide transporter at1g52190-like | |  |  |  |  |  |  |  |  |  |  |
| probable peptide nitrate transporter at3g16180-like isoform x2 | |  |  |  |  |  |  |  |  |  |  |
| ap2-like ethylene-responsive transcription factor at2g41710-like | |  |  |  |  |  |  |  |  |  |  |
| acyl-activating enzyme 6-like | |  |  |  |  |  |  |  |  |  |  |
| oleosin 5-like | |  |  |  |  |  |  |  |  |  |  |
| momilactone a synthase | |  |  |  |  |  |  |  |  |  |  |
| ring u-box superfamily | |  |  |  |  |  |  |  |  |  |  |
| wat1-related protein at3g18200-like | |  |  |  |  |  |  |  |  |  |  |
| ethylene-responsive transcription factor erf098-like | |  |  |  |  |  |  |  |  |  |  |
| thaumatin-like protein | |  |  |  |  |  |  |  |  |  |  |
| protein fizzy-related 3 | |  |  |  |  |  |  |  |  |  |  |
| adagio protein 3-like | |  |  |  |  |  |  |  |  |  |  |
| 3-ketoacyl- synthase 6 | |  |  |  |  |  |  |  |  |  |  |
| ent-kaurenoic acid oxidase 2-like | |  |  |  |  |  |  |  |  |  |  |
| peroxidase 29 | |  |  |  |  |  |  |  |  |  |  |
| receptor-like protein kinase anxur1-like | |  |  |  |  |  |  |  |  |  |  |
| immunoglobulin g-binding protein h | |  |  |  |  |  |  |  |  |  |  |
| serine carboxypeptidase 3-like | |  |  |  |  |  |  |  |  |  |  |
| zinc finger protein zat5 | |  |  |  |  |  |  |  |  |  |  |
| pleiotropic drug resistance protein 2-like | |  |  |  |  |  |  |  |  |  |  |
| flavonol 4 –sulfotransferase | |  |  |  |  |  |  |  |  |  |  |
| ccr4-associated factor 1 homolog 11-like | |  |  |  |  |  |  |  |  |  |  |
| cyanidin-3-o-glucoside 2-o-glucuronosyltransferase-like | |  |  |  |  |  |  |  |  |  |  |
| 90 KDa class I heat shock endoplasmin | |  |  |  |  |  |  |  |  |  |  |
| cyclin-dependent kinase class f4-like | |  |  |  |  |  |  |  |  |  |  |
| ethylene-responsive transcription factor 13-like | |  |  |  |  |  |  |  |  |  |  |
| abc transporter g family member 23 | |  |  |  |  |  |  |  |  |  |  |
| ethylene-responsive transcription factor erf034-like | |  |  |  |  |  |  |  |  |  |  |
| lipid-binding protein at4g00165-like | |  |  |  |  |  |  |  |  |  |  |
| 3-ketoacyl- synthase | |  |  |  |  |  |  |  |  |  |  |
| strictosidine synthase 1 isoform 1 | |  |  |  |  |  |  |  |  |  |  |
| zinc finger protein zat5 | |  |  |  |  |  |  |  |  |  |  |
| low quality protein: cytochrome p450 76C4-like | |  |  |  |  |  |  |  |  |  |  |
| 31 kda chloroplastic isoform 1 | |  |  |  |  |  |  |  |  |  |  |
| aquaporin pip2-1 isoform 2 | |  |  |  |  |  |  |  |  |  |  |
| calcium-binding protein pbp1-like | |  |  |  |  |  |  |  |  |  |  |
| 9-cis-epoxy-carotenoid dioxygenase 1 | |  |  |  |  |  |  |  |  |  |  |
| 9-cis-epoxy-carotenoid dioxygenase 1 | |  |  |  |  |  |  |  |  |  |  |
| glutathione s-transferase-like | |  |  |  |  |  |  |  |  |  |  |
| eg45-like domain containing | |  |  |  |  |  |  |  |  |  |  |
| spotted leaf | |  |  |  |  |  |  |  |  |  |  |
| beta- insoluble isoenzyme 1-like | |  |  |  |  |  |  |  |  |  |  |
| ethylene-responsive transcription factor erf017 | |  |  |  |  |  |  |  |  |  |  |
